# MACSima imaging cyclic staining (MICS) technology reveals combinatorial target pairs for CAR T cell treatment of solid tumors

**DOI:** 10.1038/s41598-022-05841-4

**Published:** 2022-02-03

**Authors:** Ali Kinkhabwala, Christoph Herbel, Jennifer Pankratz, Dmytro A. Yushchenko, Silvia Rüberg, Paurush Praveen, Sandy Reiß, Federico Carlos Rodriguez, Daniel Schäfer, Jutta Kollet, Vera Dittmer, Manuel Martinez-Osuna, Lara Minnerup, Claudia Reinhard, Andrzej Dzionek, Thomas Dino Rockel, Stefan Borbe, Martin Büscher, Jürgen Krieg, Michel Nederlof, Melanie Jungblut, Dominik Eckardt, Olaf Hardt, Christian Dose, Eik Schumann, Ralf-Peter Peters, Stefan Miltenyi, Jürgen Schmitz, Werner Müller, Andreas Bosio

**Affiliations:** 1 Miltenyi Biotec B.V. & Co. KG, Bergisch Gladbach, Germany; 2 Qi Biotech, Inc., 12261 Beestone Lane, Raleigh, NC 27614 USA; 3 Miltenyi Imaging GmbH, Radolfzell am Bodensee, Germany; 4 Division of Infection, Immunity and Respiratory Medicine, University of Manchester, Manchester, UK

**Keywords:** Biological techniques, Oncology

## Abstract

Many critical advances in research utilize techniques that combine high-resolution with high-content characterization at the single cell level. We introduce the MICS (MACSima Imaging Cyclic Staining) technology, which enables the immunofluorescent imaging of hundreds of protein targets across a single specimen at subcellular resolution. MICS is based on cycles of staining, imaging, and erasure, using photobleaching of fluorescent labels of recombinant antibodies (REAfinity Antibodies), or release of antibodies (REAlease Antibodies) or their labels (REAdye_lease Antibodies). Multimarker analysis can identify potential targets for immune therapy against solid tumors. With MICS we analysed human glioblastoma, ovarian and pancreatic carcinoma, and 16 healthy tissues, identifying the pair EPCAM/THY1 as a potential target for chimeric antigen receptor (CAR) T cell therapy for ovarian carcinoma. Using an Adapter CAR T cell approach, we show selective killing of cells only if both markers are expressed. MICS represents a new high-content microscopy methodology widely applicable for personalized medicine.

## Introduction

Analysis of cancer cell diversity and immune contexture is of high relevance for tumor subclassification and the development of novel targeted immunotherapies. The analysis can be improved by new multiplexing technologies using either optical or mass spectrometric readouts such as multi-epitope-ligand cartography (MELC)^1^, ChipCytometry^2^, mass cytometry^3^, multiplexed ion beam imaging (MIBI)^4^, cyclic immunofluorescence (CycIF)^5^, multiplex immunohistochemistry^6^, co-detection by indexing (CODEX)^7^, or InSituPlex^8^. However, there are still limitations in spatial resolution, degree of binder-based multiplexing, and tissue integrity. Transition element isotopes chelated to antibodies as used in mass spectrometric readouts are confined to about 40 unique labels^3^. Antibody-oligonucleotide conjugates used for cyclic immunofluorescence analysis need to be carefully selected, limiting these technologies to roughly 60 analytes^9^.

CAR T cell–based therapies have resulted in a remarkable success in the treatment of hematopoietic malignancies^10^, but have not yet led to a breakthrough in solid tumors. Besides inherent obstacles presented by the tissue microenvironment that can hamper T cell infiltration, there is also a lack of cell surface target molecules that are suitable for CAR T cells, i.e. high coverage of tumor cells and low on-target/off-tumor toxicity. A number of cell surface markers are currently being investigated for CAR T cell therapy in solid tumors^11^ and strategies have been outlined to combine different markers to circumvent tumor escape mechanisms (“OR” gated combinatorial CARs) or to reduce off-tumor toxicity (“AND” gated CARs)^12^.

We describe a novel imaging system for fully automated cyclic immunofluorescence analysis including a mechanism for most gentle erasure of signal with the potential to apply hundreds of binders to a single specimen. Based on a new image processing pipeline for robust removal of imaging artifacts, we demonstrate highly sensitive detection of cellular proteins at subcellular resolution, allowing deep insight into basic scientific questions such as the comprehensive identification and quantification of cellular subtypes of the spleen or visualization of tumor heterogeneity. Applied to the screening of glioblastoma multiforme (GBM), high-grade serous ovarian carcinoma (HGSOC), and pancreatic ductal adenocarcinoma (PDAC), MICS-based screening reveals marker combinations with a preferable on-target/on-tumor vs. on-target/off-tumor profile suitable for CAR T cell development. We validate this screening approach using Adapter CAR T cells for an AND gated combinatorial targeting of cancer cells co-expressing EPCAM and THY1.

## Results

### Cyclic immunofluorescence staining with the MACSima™ Imaging Platform

**Figure 1:**
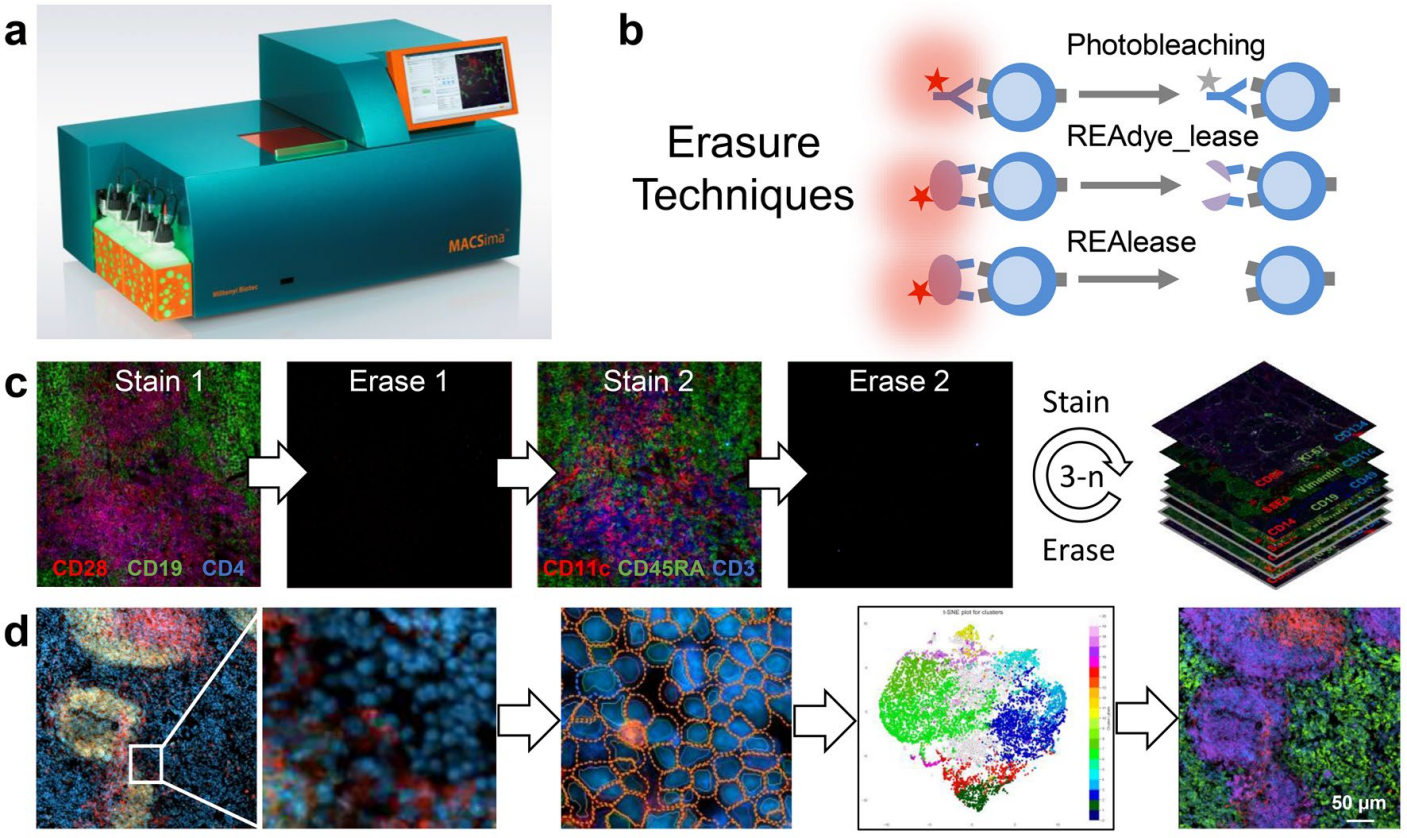
Cyclic imaging with the MACSima Imaging Platform. (**a**) MACSima System with fully automated robotic liquid handling and image acquisition. (**b**) Erasure techniques based either on photobleaching of the dye, or disruption of the labeling conjugate by release reagent. The release reagent leads to a rapid detachment of the fluorescent dye only (REAdye_lease Antibodies) or disruption of the labeling conjugate with a spontaneous dissociation of the monomerized antibody fragments and the fluorescent dye (REAlease Antibodies) from their target epitopes. (**c**) Cyclic imaging. (**d**) Image analysis with the MACS iQ View Software consisting of cellular segmentation, clustering, and visualization of clustered cells across the original image.

The MACSima™ Imaging System (Fig. 1a) operates by iterative immunofluorescent staining, sample washing, multi-field imaging, and signal erasure (Fig. 1b), using three fluorochrome-conjugated antibodies per cycle (Figs. 1c, S1). Standard and novel image processing algorithms were employed to remove imaging artifacts and to maximize the signal-to-noise ratio (Fig. S2). An in-depth analysis of the image stacks generated on the instrument was achieved using a novel software (MACS iQ View, Miltenyi Biotec) that allowed us to navigate through the image stack, segment the tissue images into single cells and cluster cells according to their expression profiles (Fig. 1d). A liquid handling system was programmed to prepare the antibody conjugate staining solution, apply it to the biological sample, and wash the sample after the staining or enzymatic release incubation step. Images were obtained by a widefield microscope in an epifluorescence setup with a 20 × or 2 × objective using infrared, red, green, blue, and UV LEDs and filters. For signal erasure we first tested photobleaching of FITC-, PE-, and APC-conjugated hybridoma antibodies or recombinantly engineered REAfinity Antibodies consisting of a mutated form of the human IgG1 backbone that obviates the need for FcR blocking (Figs. 1b and S1a). Remaining signals were imaged separately and subtracted from the subsequent image (Figs. S3, S4). While photobleaching was efficient, it also prolonged the overall processing time, limited cyclic immunofluorescence to the use of photobleachable dyes and showed a tendency to slightly disrupt non-crosslinked specimens such as acetone-fixed samples. Therefore, we developed a new type of binder-fluorochrome conjugates. Recombinantly engineered antibody fragments were generated, multimerized, fluorescently labeled and optimized to bind to epitopes on sections with high avidity. Disruption of the complex led either to the release of the fluorescent dye only (“REA_dyelease”) (Fig. S1b) or to the release of dye and binder fragment in case low epitope binding affinities were selected (“REAlease”) (Fig. S1c,d). Furthermore, the three methods (photobleaching, REAdyelease, REAlease) for signal erasure can be used in combination for the same cycle of imaging using binders conjugated to different fluorescent dyes. No deleterious effects on the efficiency of erasure were observed upon combined application of these three methods in the same cycle. With the antibodies currently available for our experiments, roughly 120 for formaldehyde-fixed paraffin-embedded (FFPE) material and 327 for paraformaldehyde (PFA)-fixed material, we did not observe deterioration of the material, and therefore we did not notice a limit on the number of immunofluorescent cycles that could be applied to a single sample. For example, using PFA-fixed material, more than 300 unique antibodies could be applied to the same specimen (Fig. S5). Based on systematic testing of antibody conjugates, we so far generated a library of about 2035 fluorochrome conjugates, covering roughly 554 distinct epitopes of biomolecules and qualified them for the analysis of human or mouse acetone-, PFA- or FFPE-fixed samples.

### MICS analysis reveals a highly linear dynamic range over five orders of magnitude with a sensitivity down to a few proteins per cell

**Figure 2:**
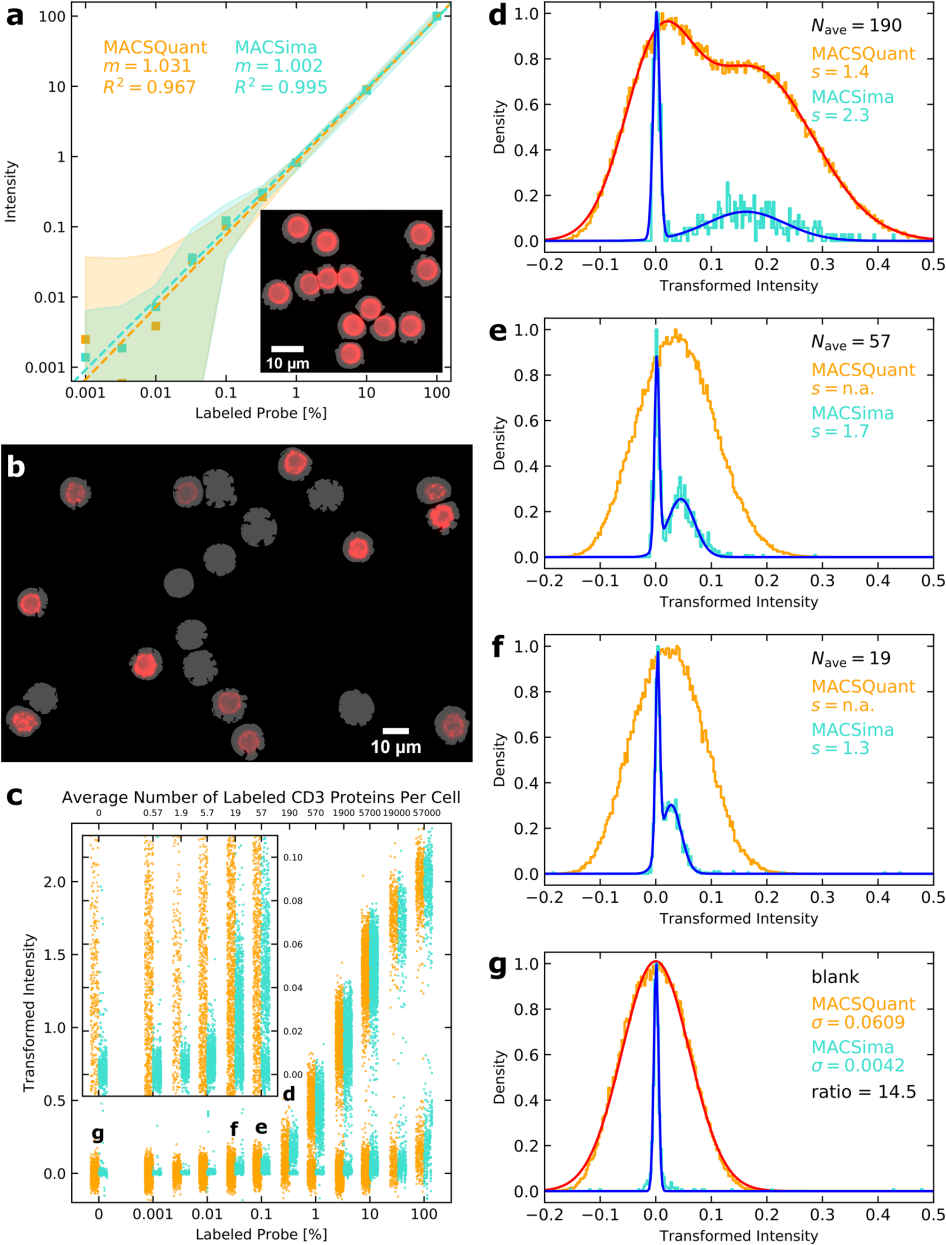
Comparison of linearity and sensitivity of imaging-based cytometry on the MACSima System with flow cytometry on the MACSQuant Analyzer. (**a**) Means (square symbols) and ± 1*σ* ranges (shaded regions) describing the population distributions for beads incubated with different percentages of APC-labeled vs. unlabeled antibodies measured by the MACSQuant Analyzer (orange) and MACSima Imaging System (cyan). For the images (see inset), bead autofluorescence in the DAPI channel was used to create individual bead masks (gray) over which the total background-subtracted APC signal could be determined. Least-squares line fits on the log–log axes are shown (dashed lines), with the optimal slopes, *m*, and corresponding *R*^2^ values listed for each dataset. (**b**) Image of PFA-fixed PBMCs labeled with a CD3-APC antibody (red) and a CD45-VioBlue antibody, with the latter staining used to generate the displayed masks (gray) for each CD45^+^ cell. (**c**) CD45^+^ PBMCs were assessed for their CD3-APC co-staining for different stoichiometric amounts of an APC-labeled vs. unlabeled CD3 antibody on the MACSQuant Analyzer (orange) and MACSima Imaging System (cyan). The upper *x*-axis displays the approximate average number of labeled CD3 proteins per CD3^+^ cell for each assayed percentage, based on the previously measured^26^ average copy number of CD3 per cell amounting to 57,000. Only a small representative subset of the full MACSQuant Analyzer data (the latter ranging from 54,581 to 167,637 cells) is displayed for each assayed percentage of labeled probe to match the respective number of cells measured by the MACSima Imaging System (ranging from 417 to 2384 cells). The inset gives a zoomed-in view of the *y*-axis for better visualization of the scatter plot distributions. (**d–g**) Histograms for specific assayed percentages of labeled probe are shown based on the complete datasets from both instruments. For *N*_ave_ = 190 (**d**), the CD3^+^ cell population is recognizable in both datasets as a second peak, permitting a double Gaussian fit and consequent determination of the separation parameter, $s$. For *N*_ave_ = 57 (**e**) and *N*_ave_ = 19 (**f**), a second peak is only distinguishable in the MACSima Imaging System’s dataset. The width of the distribution for the blank (**g**) provides a useful estimate of the measurement error expected for weak signals on both instruments.

To determine the linearity and sensitivity of MICS and for comparison with flow cytometry on a MACSQuant Analyzer, we generated fluorescently labeled beads (Fig. 2a, inset) with distinct brightnesses by conjugation with different stoichiometries of fluorescent (APC) to dark (biotin) labels. Image cytometry with the MACSima Imaging System was significantly more sensitive for lower intensity beads (narrower shaded area in Fig. 2a), approaching 9 × higher sensitivity (9 × smaller standard deviation) for completely unlabeled beads. To compare sensitivities for cell-based targets, single-donor peripheral blood mononuclear cells (PBMCs) were measured. PBMCs were stained by different stoichiometric ratios of APC- and biotin- labeled CD3 antibodies (Fig. 2b). The intensity distribution of the stained vs. unstained cell subpopulations is plotted with estimated numbers of stained epitopes per cell (Fig. 2c) and individual histograms are depicted (Fig. 2d–g) (note the reference labels to these subpanels in Fig. 2c). To illustrate the discrimination power of the MACSima Imaging System and MACSQuant Analyzer, we report the separation parameter, $s$, for a double Gaussian fit where possible (Table 1). This parameter is defined as the difference in means of two populations divided by the square root of the sum of their individual variances^13^ and is therefore a quantitative readout of the ability to resolve two different populations^14^. For the MACSQuant Analyzer, a double-peaked population was still detectable for an average number of labeled epitopes of roughly 190 (*s* = 1.4, Fig. 2d). A second peak was still detectable (Fig. 2e,f) for the MACSima Imaging System even down to roughly 19 labeled epitopes (*s* = 1.3, Fig. 2f), indicating roughly 10 × more sensitivity than the MACSQuant Analyzer. In addition, the distribution for the blank control was much narrower for the MACSima System. Based on the ratio of the widths of the blank distributions, the MACSima Imaging System was found to be 14.5 × more sensitive than the MACSQuant Analyzer (Fig. 2g), agreeing well with the factor of 10 × obtained above from the separation parameter.

**Table 1:** Stoichiometric labeling percentages, average expected number of labeled CD3 proteins per cell^26^, and separation parameters, *s*, for the MACSQuant Analyzer and MACSima Imaging System.

Labeling percentage	Labeled CD3 Proteins per cell	MACSQuant separation	MACSima separation
100	57,000	9.77	8.41
33.0	19,000	9.19	9.62
10.0	5700	7.56	7.52
3.30	1900	5.11	4.78
1.00	570	2.87	2.76
0.330	190	1.41	2.29
0.100	57	n.a	1.73
0.0330	19	n.a	1.33
0.0100	5.7	n.a	n.a
0.00330	1.9	n.a	n.a
0.00100	0.57	n.a	n.a

The results shown in Fig. 2 for the linearity and sensitivity of the MACSima Imaging System as compared to the MACSQuant Flow Cytometer were acquired on the same reference sample to enable direct, unbiased comparison of both instruments, thereby providing a useful performance baseline for the MACSima System. However, for a standard MICS run, additional corrections are applied in the full image processing pipeline (Fig. S2). These further improvements come primarily from the photobleaching of initial autofluorescence, the subtraction of remaining autofluorescence or residual staining from each new stained image (Figs. S3 and S4), and the statistically optimal inference of individual pixel intensities for the generation of a high dynamic range (HDR) image (further details will be published separately).

### In-depth analysis of mouse spleen tissue validates the performance of MICS and identifies at least 20 distinct cell types including rare cells with a frequency of 30–40 in about 10,000 cells

**Figure 3:**
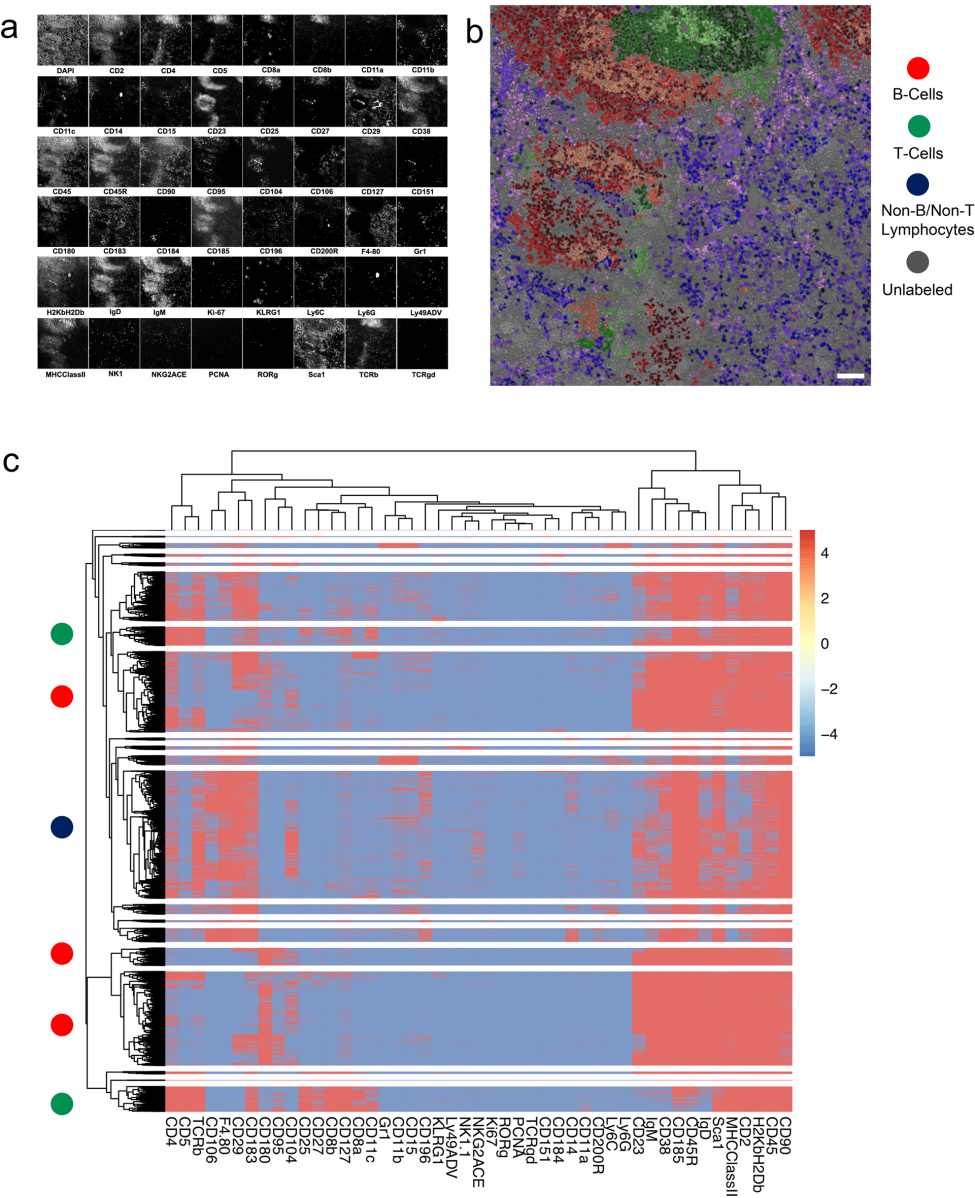
Multi-channel analysis of a mouse spleen section on the MACSima Imaging System. A mouse spleen section was fixed by acetone and subsequently stained by directly fluorescently labeled antibodies in subsequent cycles. DAPI was used to stain the nuclei in the section, and it was used to register the images across the cycles and to segment the nuclei of the cells in the section. (**a**) Small images of each of the channels used to generate the more complex data visualizations in the subsequent panels. In total, 47 antibody staining images and one DAPI image are shown. (**b**) Result of an extensive k-means (40 clusters) clustering of the staining intensity values of cells after segmentation is shown, in which four cell populations are colored, with B cells in red, T cells in green, Non-B/Non-T cells in blue, and cells not recognized by the antibody panel in gray (17 clusters). The different color changes within the red (9 clusters), green (7 clusters) and blue (17 clusters) represent distinct subpopulations within the respective groups. (**c**) Results of hierarchical clustering (20 clusters) based on the mean staining intensity values of individual cells and a marker expression heat map of the various subpopulations. On the left side a colored dot inticates if a cluster consists mainly of cell types as classified in **b**, namely B or T cells or non-B/non-T cells.

To demonstrate the power of the technology applied to tissue, we performed a MICS analysis on a frozen section of a mouse spleen using 47 antibody markers spanning as many types of immune cells as possible. For this tissue, expression of key proteins and the cellular composition has been extensively characterized before^15^. Figure 3a shows the antibody stainings used for subsequent analyses. Higher resolution images are shown in the supplement (Fig. S6). To generate the image in Fig. 3b, expression levels of individual markers at the single-cell level obtained after segmentation were grouped into 40 clusters using k-means clustering. Then the clusters were subdivded into four main groups of cell types: B cells, staining with anti IgM or IgD antibodies; T cells, staining with anti T cell receptor antibodies; Non-B/Non-T lymphocytes, giving signals in at least one other marker stained; and very low stained cells with the selected markers, representing most likely stroma cells. Within each group, the clusters were stained using a color gradient per cell type. By a sequential gating strategy we were able to identify rare cell types. For example, 40 IgM plasma cells and 38 CD8 alpha/alpha-positive T cells were found in the section consisting of 11,996 segmented cells (Fig. S7). Both cell types can be found in the red pulp of the spleen next to the white pulp. To detect such rare cell populations from two separate cell lineages in a complex cell population would be almost impossible in a single flow cytometry experiment. In Fig. 3c, we show a heatmap based on hierachical clustering with 20 clusters. Violin plots for the different markers for each cluster are displayed in Fig. S8. In conclusion, high-content staining using MICS technology can differentiate multiple cell populations in higher resolution and with additional spacial information in a single experiment that may be impossible with flow, or at minimum require extensive flow cytometric analysis employing multiple complex antibody marker mixtures and gating strategies. MICS technology can also be used for the analysis of cell suspensions based on many more markers and with a better discrimination of cell clusters compared to multicolor flow cytometry.

### MICS reveals novel pairs of surface proteins as candidates for CAR T cell-based immunotherapies

**Figure 4:**
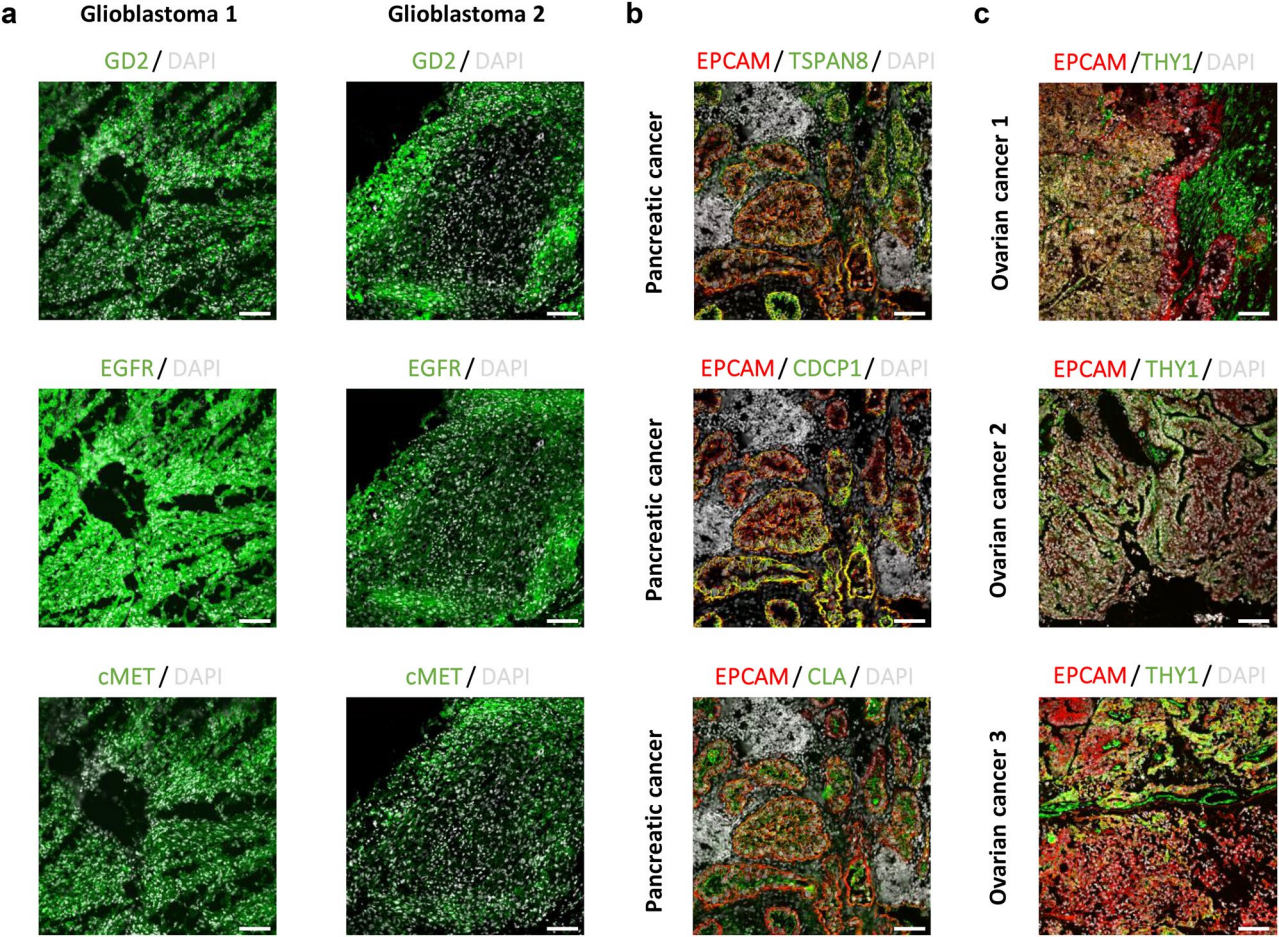
Ultrahigh-content imaging enables the discovery of novel targets and target pairs in different cancer indications which can be targeted and lysed in vitro by Adapter CAR T cells. (**a–c**) Fresh-frozen human GBM, PDAC, and HGSOC samples were sliced and analyzed by MICS. DAPI is shown in white and the indicated markers of interest are shown in green or red. Scale bar represents 100 µm. (**a**) Examples of MICS analysis of two GBM patient samples showing the expression of GD2, EGFR and cMET. (**b**) Example of a PDAC patient sample analysis displaying the co-expression of EPCAM (red) as a general epithelial and tumor cell marker with the potential PDAC marker candidates (green) TSPAN, CDCP1 and CLA. (**c**) Examples of three HGSOC samples. Sample 1 and 2 co-express EPCAM (red) and THY1 (green), while sample 3 expresses EPCAM but not THY1.

**Figure 5:**
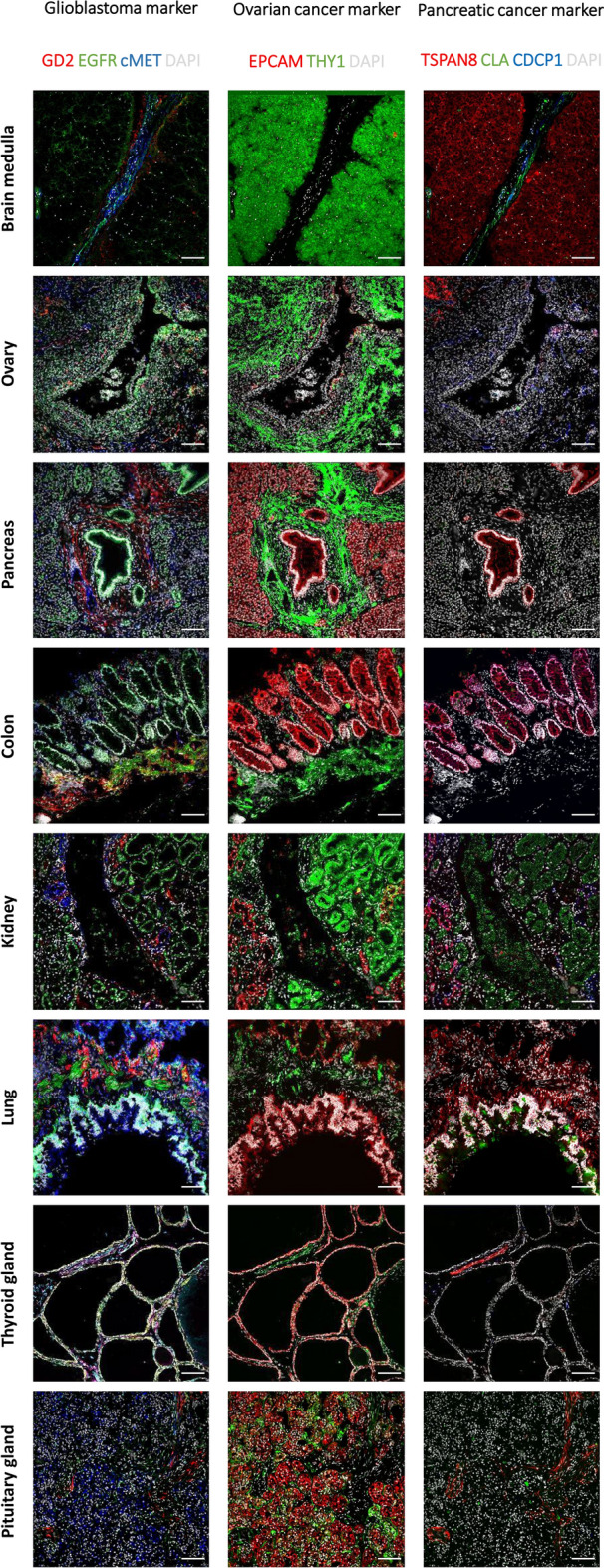
Ultrahigh-content imaging validates expression of target candidates on healthy human tissues to predict safety and toxicity of target candidates. Fresh-frozen human tissues were sliced and fixed with acetone. The subsequent screening was performed on the MACSima Imaging Platform by employing a sequential staining of antibodies. Healthy human tissues, i.e. medulla oblongata, ovary, pancreas, colon, kidney, lung, thyroid gland, and pituitary gland, were analyzed for the expression of glioblastoma target candidates (left panel), GD2 is shown in red, EGFR in green, cMET in blue, and DAPI in white; ovarian cancer target candidates (middle panel), EPCAM is shown in red, THY1 in green, and DAPI in white; pancreatic cancer target candidates (right panel), TSPAN8 is shown in red, CLA in green, CDCP1 in blue, and DAPI in white. Scale bar represents 100 µm.

**Figure 6:**
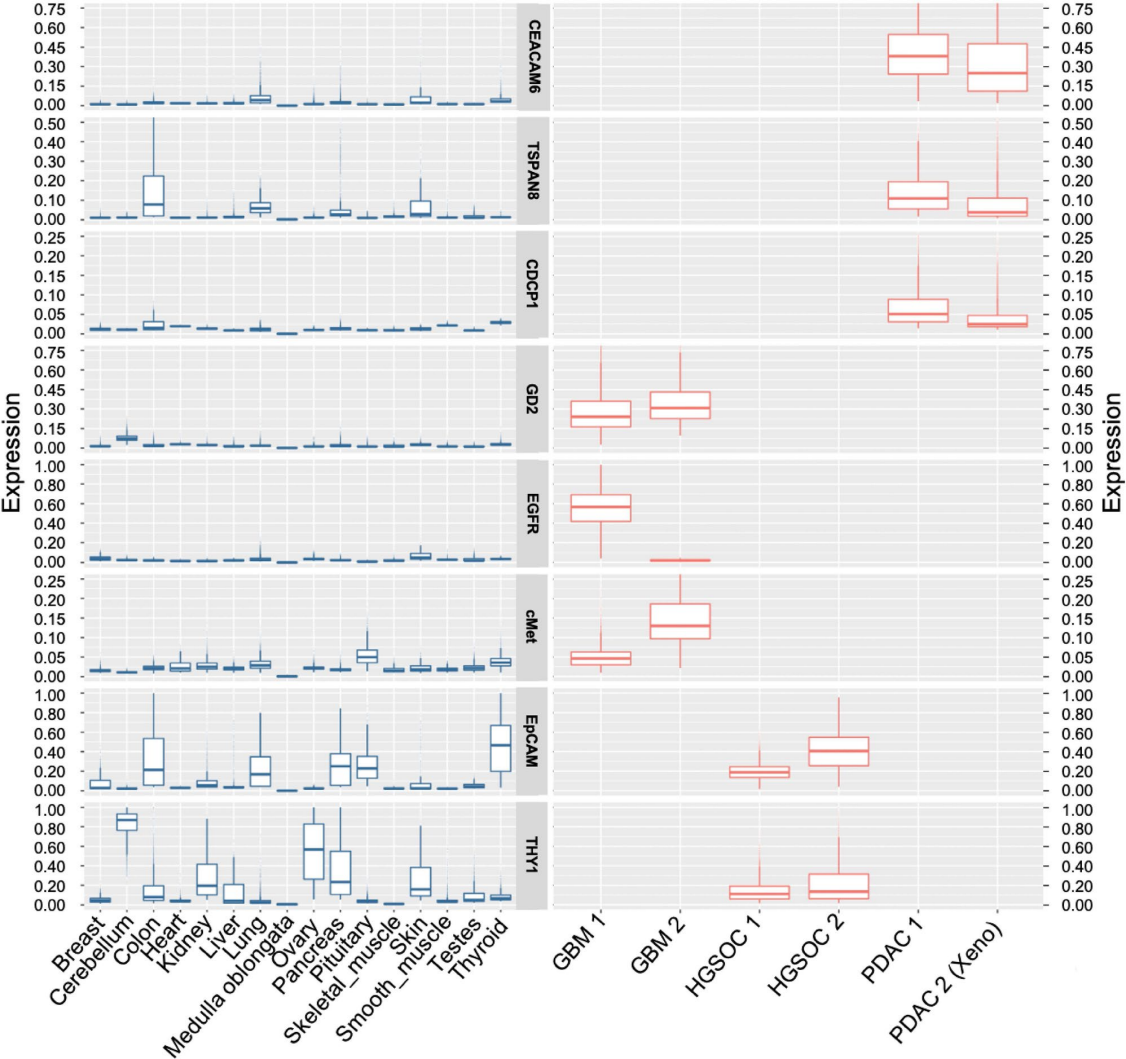
Comparison of marker expression in healthy and cancer samples. Tumor marker expression was quantified on a single-cell level for healthy (left panel) and tumor (right panel) tissue. Image data sets were segmented using MACS iQ View Software based on nuclei and cell membrane markers identifying individual cells. Background-subtracted mean fluorescent intensities (MFI) were computed for each cell and scaled between 0 and 1 for visualization and comparability. Values for all cells detected in one tissue image are displayed as box plots. Note that the scale of expression is kept constant for each marker across all tissues but varies between the markers for optimal representation. The ovarian cancer samples showed a weak but significant positive correlation of 0.35 and 0.20 (significant at α = 0.01; *P* value = 1.64e−311 and 5.63e−49, respectively) while healthy ovary tissue showed a weak but significant (α = 0.01; *P* value = 3.89e−107) negative correlation of − 0.23. The marker pair showed comparatively higher positive correlation of 0.58 in healthy breast tissue, but an overall very low expression level. Please note that expression values are only presented for tissues of interest.

A second application of MICS technology aims to find markers selectively expressed on solid tumor cells but not in healthy tissue to develop cell-based immunotherapies. In general, the identification of suitable targets for CAR T cell therapy of solid tumors has been difficult as most targets either do not cover a large enough percentage of tumor cells within a tumor and across different patients, or are not exclusively expressed on tumor material but also present on healthy cells. Therefore, we have followed the strategy to first screen for targets which are broadly expressed on tumor material to allow for highest efficacy when targeted by CAR T cells. We have then selected from the first screen those targets that show less expression in healthy tissue either as a single marker or as a combination of two markers. For this, we performed high-content expression analysis of proteins across several different tumor entities, specifically GBM, HGSOC and PDAC. First, GBM xenografts derived from primary tumors were analyzed for 371 cell surface markers by flow cytometry. 96 markers were selected according to the percentage of positive cells and their stain index. Re-analysis of eight primary GBM by MICS showed the broadest and/or strongest expression for GD2, EGFR and cMET on tumor cells, but also a broad inter- and intratumor diversity (Figs. 4a and 6). To assess the respective toxicity profile, expression in over 16 different human tissue samples (Figs. 5, 6, S9) was measured. We observed expression of GD2 in the brain (cerebellum, medulla oblongata), and a weak expression in healthy lung, pancreas, liver, ovary, skin, thyroid and testes tissues. EGFR was expressed in most healthy tissues and cMET primarily in pituitary glands, thyroid, lung, kidney, and heart. We compared the Pearson correlation coefficients for healthy and GBM samples with three marker pairs, namely, GD2-EGFR, GD2-cMet and EGFR-cMet. The marker pair GD2-EGFR showed a stronger and significant correlation of up to 0.70 in GBM tissue as compared to the healthy brain tissue samples. For GD2-cMET and EGFR-cMET we found a higher correlation in non-GBM than GBM tissue. In conclusion, an AND gated combination of GD2 and EGFR appears to be most promising for a CAR T cell based immunotherapy for GBM of the classical/mixed subtype^16^.

Using a similar approach as described above, we previously reported the analysis of PDAC^17^. There we identified CDCP1 (CD318), tetraspanin-8 (TSPAN8), cutaneous lymphocyte-associated antigen (CLA) and CEACAM6 (CD66c) as target candidates for CAR T cell-based immunotherapy. CDCP1 showed the most favorable pattern with almost no detectable protein expression in healthy tissues, while e.g. TSPAN8 and CEACAM6 raised safety concerns with respect to their expression in gastrointestinal and hematopoietic tissues, respectively. We re-evaluated and extended the data by MICS, specifically aiming at identifying promising target pairs (Figs. 4b, 5, 6, and S9). After re-analyzing the co-expression of these markers, we concluded that an AND gated target combination of CDCP1 with TSPAN8 or CEACAM6 could be promising from a safety perspective of a CAR T cell therapy (Fig. 6), while development of CLA-specific CAR T cells requires further technical solutions as it is expressed on activated CAR T cells^17^.

Finally, we applied our screening approach to HGSOC. We found that EPCAM was reliably expressed on all ovarian cancers, showing a high coverage of cells in individual ovarian cancer samples (Figs. 4c, 6). As EPCAM is expressed on most epithelial cells, we searched for a second marker co-expressed on ovarian cancer cells but not on non-cancerous epithelial cells. We identified THY1 with a characteristic expression on non-epithelial cells like fibroblasts (Fig. 4c) and co-expression with EPCAM on ovarian cancer cells (Fig. 4c). Over all tissues analyzed, we saw minor co-expression of EPCAM with THY1 on structures within kidney and breast tissue (Fig. S9). The HGSOC samples showed a weak but significant correlation for cellular co-expression of EpCAM and THY1, while on healthy tissue we observed only for breast tissue a high correlation, however, on a very low expression level (Fig. 6). In summary, MICS screening across multiple markers allowed us to identify marker pairs that could potentially be used to specifically eradicate tumor cells while not harming non-tumor cells in an AND gated application of CAR T cells.

### Adapter CAR T cells allow the combination of two tumor markers for an AND gated specific killing of tumor cells

**Figure 7:**
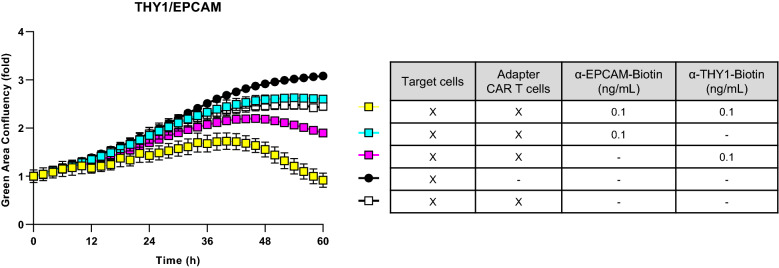
Adapter CAR T cells targeting THY1 and EPCAM are selectively cytolytic against ovarian cancer cell line co-expressing THY1 and EPCAM. Primary human T cells were isolated and transduced with a CAR construct against biotin. Anti-biotin CAR T cells were co-cultured with THY1-, EPCAM-, and GFP-expressing target cells for 72 h in the presence of suboptimal single adapter doses or with the combinatorial use of suboptimal adapter doses. GFP-fluorescence was measured over time. CAR T cell-mediated lysis of target cells resulted in decreased GFP-fluorescence. Combined treatment with THY1- and EPCAM=reactive adapter antibodies triggered target cell lysis at concentrations which were too low to cause CAR T cell-mediated target cell lysis when applied separately. Each data point represents mean of a technical replicate ± SEM. Representative graph from three experiments shown.

After having identified marker pairs for GBM, PDAC, and HGSOC, we considered Adapter CAR T cells as an attractive system for multi-targeting of cancer cells. Here, CAR T cells are directed against a tag moiety, i.e. biotin. Consequently, the targeting of the CAR T cells to the respective cancer cells is achieved via the addition of adapter molecules such as biotinylated antibodies, Fab fragments, or other antibody fragments. Adapter molecules can be quickly exchanged, differentially dosed, and removed from the system allowing dynamic interactions^18,19^. To test one of the identified candidate pairs, we generated ovarian cancer cells co-expressing EPCAM and THY1 in combination with eGFP. We performed in vitro CAR T cell functionality testing on these cells by titrating varying doses of biotinylated anti-EPCAM (Fig. S10a) and anti-THY1 (Fig. S10b) antibodies in the presence of Adapter CAR T cells. These co-culture assays revealed defined ranges of antibody concentrations that initiate CAR T cell activation indicated by target cell lysis. Notably, the combination of antibodies can cause CAR T cell–mediated target cell lysis at concentrations that are ineffective when using only a single antibody (Figs. 7d, S10c). Thus, adapter molecules at suboptimal doses for single targeting approaches could be used in combination to lyse tumor cells and prevent potential off-tumor toxicity.

## Discussion

Here we describe the MICS system, which has the potential to overcome limitations of current multiparametric technologies and to complement single-cell experiments. As the tissue samples are left intact, MICS is compatible with other technologies and thereby provides a first step for high-content, single-cell analyses for basic research as well as a basis for similar high-content, single-cell analyses for pathology in a regulated environment. Using fluorescently labeled binders and a gentle signal erasure process, we combine the advantage of direct, highly sensitive fluorescent imaging with subcellular resolution and the ability to apply more than 300 binders to the same specimen.

Mass spectrometry is limited by the number of transition element isotopes^3^ and existing cyclic immunofluorescent approaches are limited as they either apply all antibodies at once and have a secondary oligonucleotide-based readout and/or use harsh and complex chemistry, i.e. peroxides for signal bleaching, antibody crosslinking chemistry, or oligonucleotide hybridization^5,9^. Our directly conjugated binders circumvent the need to control and quantify a secondary binding step (secondary antibodies or oligonucleotides) and our antibody conjugate panels can also be arranged in a much more flexible way. We report a sensitivity equivalent to about 19 proteins per cell, approaching the sensitivity of super-resolution microscopy^20^, as well as a high degree of linearity over five orders of magnitude. Both sensitivity and specificity to detect rare cells are also increased by multiplexing, with successful detection of rare cells limited only by the total number of imaged cells. The high reproducibility of our approach is reflected by the possibility to restain the same epitope (Fig. S1).

Common problems with multiparametric analysis are the complexity of handling hundreds of binders and the duration of the experiment. We address these problems by automating the process and by reducing the processing time by selecting for binders that are sensitive, specific and efficient in binding within the 10 min incubation time. Image acquisition requires roughly 10 min, with signal erasure also requiring approximately 10 min. This sums up to roughly 20 h for a typical experiment consisting of 34 cycles and a total of 100 antibody markers. This can be further reduced in the future by increasing the image acquisition speed and the number of fluorescence channels.

Targeted immunotherapy has achieved clinical benefits for patients in recent years^21,22^. A potential threat is still on-target/off-tumor toxicity^23,24^. Currently, most prediction methods for on-target/off-tumor expression are based on mRNA expression data. These prediction models, however, are limited by poor correlation between RNA and protein expression levels. Simultaneous targeting of different antigens should reduce the risk of on-target/off-tumor toxicity in cell therapies like CAR T cells^25^. We introduce MICS as a pre-clinical assay and suggest target pairs for GBM, HGSOC, and PDAC based on their expression on tumor cells and lack of expression on healthy tissue. One of the target pairs consists of THY1 and EPCAM, which were co-expressed on ovarian cancer cells in a subset of HGSOC but not on healthy tissue. In vitro validation with Adapter CAR T cells for an AND gated targeting shows efficient killing of double-positive cells only. While this is a promising approach to reduce potential toxicity of CAR T cells, the killing of only the double positive cells may increase the risk of tumor escape. However, Adapter CAR technology opens the possibility to tackle multiple targets and target pairs sequentially and in a controllable fashion. MICS, as an efficient method for multiparametric analysis of human tumor samples, will be instrumental in guiding the choice of appropriate targets.

## Methods

### Tissues, PBMCs, cell lines and culture conditions

High-grade serous ovarian cancer patient samples were provided by Prof. Dr. Peter Mallmann (Department of Obstetrics and Gynecology, Faculty of Medicine, University of Cologne), pancreatic ductal adenocarcinoma were provided by Prof. Dr. Philipp Stroebel (Institute of Pathology, University Medical Center Göttingen), and glioblastoma samples were provided by Prof. Dr. med. Wolfgang Brück (Institute for Neuropathology, University Medical Center Göttingen) or purchased from BioIVT and used with informed consent by the patients. Fresh-frozen healthy organ tissue samples were purchased from BioIVT and ProteoGenex. All pancreatic ductal adenocarcinoma (PDAC) patient**-**derived xenografts (PDX) were obtained from Charles River Discovery Research Services Germany GmbH.

The mouse frozen embedded tissue section was derived from a frozen C57BL/6 mouse spleen provided by the Czech Centre for Phenogenomcis by PD Dr. rer. nat. Radislav Sedlacek and is part of a control group of a bigger experiment characterizing mouse mutants using the MICS system. These results will be published separately.

Buffy coats and leukapheresis products were obtained from the University Hospitals in Cologne and Dortmund. Peripheral blood mononuclear cells (PBMCs) were isolated from buffy coats by density gradient centrifugation. Before imaging, PBMCs were washed 3 × in PBS and transferred to multiple wells of a glass-bottomed (170 μm coverslip) 24-well plate (with 1 × 10^6^ PBMCs /well). They were then centrifuged at 1000×*g* for 10 min (RT), fixed for 10 min using 4% PFA, and finally washed (3×) with PBS buffer.

HEK293T and A2780 cells were purchased from American Type Culture Collection (Manassas, VA, USA) and cultured in DMEM (Biochrom, Nuaillé, France) supplemented with 2 mM glutamine (Lonza, Basel, Switzerland) and 10% FCS (Biochrom, Berlin, Germany). Cell confluency ranged typically between 20 and 80% during the culture maintenance phase. All human primary cellular products were derived from healthy donors after informed consent and cultivated in TexMACS medium (Miltenyi Biotec, Bergisch Gladbach, Germany).

### Flow cytometry

Tissues were dissociated using the Tumor Dissociation Kit, human in combination with the gentleMACS™ Octo Dissociator with Heaters (both Miltenyi Biotec). When processing PDX models, mouse cells were depleted using the Mouse Cell Depletion Kit (Miltenyi Biotec). Resulting cell suspensions were analyzed using the MACS Marker Screen, human (Miltenyi Biotec), a monoclonal antibody panel containing 371 pre-titrated antibodies with nine isotype controls, or candidate antibodies selected from this panel for subsequent screening steps. All samples were measured on a MACSQuant Analyzer and analyzed using the MACSQuantify™ Software or FlowJo v10.7.1.

### Tissue processing for microscopy

Frozen embedded tissue specimen were cryosectioned with a CM3050 cryostat (Leica), 8 µm sections were mounted on SuperFrost Plus slides (Menzel). For acetone fixation the sections were fixed in − 20 °C cold acetone for 3 min prior to the slide storage at − 80 °C. On the day of use, the slide was immersed in − 20 °C cold acetone for 10 min for thawing. After short air drying the appropriate MACSwell Imaging Frame was mounted immediately on the slide and the appropriate initial sample volume of MACSima Running Buffer was added (according to the MACSwell Imaging Frames data sheet). For PFA fixation, the cryosectioned slices on slides were directly stored at − 80 °C. On the day of use, the frozen slide was put in a 4% PFA solution and incubated for 10 min at room temperature. The slide was washed three times with MACSima Running Buffer. After washing, the appropriate MACSwell Imaging Frame was mounted immediately on the slide and the appropriate initial sample volume of MACSima Running Buffer was added (according to the MACSwell Imaging Frames data sheet). Right before the start of the MACSima Instrument, a DAPI pre-staining was performed: The MACSima Running Buffer was removed from the sample to be analyzed and stained for 10 min with a 1:10 dilution of a DAPI staining solution (volume depends on working volume for the different formats of the MACSwell Imaging Frames, see data sheet). The DAPI staining solution was removed and three washing steps were performed (MACSima Running Buffer). Finally, the initial sample volume of MACSima Running Buffer was added.

### Antibodies and conjugates for microscopy

The following three types of reagents were used in this work for iterative staining and imaging with the MACSima System: fluorescently labeled antibodies, REAdye_lease and REAlease Reagents. Fluorescent antibodies were prepared by chemical linking of one or more fluorescent dyes to a corresponding recombinant REAfinity Antibody. Recombinant antibodies are derived from a defined set of genes, ensuring the consistency in antibody structure and performance. REAfinity Recombinant Antibodies are constantly validated for reproducibility, specificity, and sensitivity. Purity and reproducibility of production are tested by mass spectrometry analysis of the purified recombinant antibodies. Lot-to-lot consistency is tested at two stages: (1) during the antibody raw material production and (2) the fluorochrome conjugation process. The latter includes purification steps to remove unconjugated fluorochromes and antibodies from the mixture and side-by-side comparisons with previous batches. Specific binding of REAfinity Antibodies to their target antigens is first validated by an epitope competition assay where cells are incubated with an excess of purified unconjugated REAfinity Antibody followed by staining with fluorochrome-conjugated antibodies of other known clones against the same marker. Second, a knockout validation via targeted genome editing or knockdown using RNA interference is performed where the target gene is knocked out in a suitable cell line using site specific nucleases (confirmed by sequencing of the target locus) or the translation of the target RNA is inhibited by transfecting cells with small non-coding RNA oligonucleotides. The antibody is considered to bind specifically to the intended epitope, if no (knockout) or reduced (RNAi) antibody binding to the respective cells can be detected. Finally, the sensitivity of an antibody in an intended application is tested on primary samples including a test to verify that the epitope of an antigen can still be detected after a given fixation process. Further details regarding the antibody conjugations used in this publication can be found here: https://www.miltenyibiotec.com/DE-en/products/macs-antibodies/releasable-fluorochromes.html.

A typical example of recombinant fluorescent antibody used in this work is CD4-FITC (CD4 Antibody, anti-human, FITC, REAfinity™, 130-114-531). REAdye_lease Reagents were prepared by covalently linking the fluorescent label and two or more antibody fragments to a dextran molecule. The dextran linker between the antibody fragments and the fluorescent label can be cleaved by incubating the labeled cells with dextranase for 10 min at 21 °C, which leads to erasure of the fluorescence staining. A typical example of a REAdye_lease Conjugate used in this work is CD11c-PE (CD11c Antibody, anti-human, PE, REAdye_lease 130-121-314). REAlease Reagents were prepared by covalent linking and fluorescent labeling of two or more antibody fragments that are characterized by low epitope binding affinities. Due to multimerization of antibody fragments, REAlease Conjugates possess high avidity and are comparable to conventional antibody-fluorochrome conjugates with respect to their labeling properties. The dextran linker between the antibody fragments can be disrupted in the presence of dextranase as the releasing reagent. This leads to monomerization of the antibody fragments and their dissociation from the epitopes, resulting in both erasure of the fluorescence staining and demasking of the epitope for restaining. Typical examples of REAlease Conjugates used in this work are CD8-FITC (CD8 Antibody, anti-human, FITC, REAlease, 130-112-070) and CD19-FITC (CD19 Antibody, anti-human, FITC, REAlease, 130-112-073). To characterize the expression of proteins in the murine spleen and on cancerous and healthy human tissues, various antibody panels were designed. A list of all antibodies used in the respective experiment is given in Supplementary Table 1.

### Cyclic immunofluorescence staining with the MACSima Imaging Platform

The MACSima Imaging System is a fully automated instrument combining liquid handling with widefield microscopy for cyclic immunofluorescence imaging. In brief, staining cycles consisted of the following automated steps: immunofluorescent staining, sample washing, multi-field imaging, and signal erasure (photobleaching or removal of REAlease Reagents). Cyclic immunofluorescence with the MACSima Imaging System is optimally applied on thin tissue cryosections (few microns thick), cultured cells, or suspension cells (either captured in microcavities or centrifuged onto the glass). We developed MACSwell Sample Carriers specifically to provide the reaction cavities necessary to perform MICS experiments with the MACSima Imaging Platform. To support either tissue sections of varying size or adherent cells we designed different kinds of sample carriers: MACSwell One, Two, and Four Imaging Frames, and MACSwell 24 Imaging Plates.

#### Liquid handling

The liquid handling system comprises a syringe pump, peristaltic pumps, a valve head, tubing, and a robotic needle. The valve head connects the needle with the externally mounted buffer bottles (MACSima Running Buffer, MACSima Storage Solution, MACSima System Buffer) and the waste bottle. The valve head allows for switching of the syringe pump to each of the bottles and the needle. To avoid carryover between pipetting steps, needle washing is performed by flushing the needle from both inside and outside with fresh buffer. Additional peristaltic pumps transport the used fluids to the waste bottle. An internal reagent bank has positions for other reagents that can be accessed with the needle (e.g. DAPI, FcR blocking reagent, REAlease Release Reagent). The liquid handling system automates in particular the following steps: (1) preparation of the antibody conjugate staining solution (e.g. resuspension of dried antibody conjugates, dilution/mixing of conjugates), (2) application of the staining solution or the REAlease Release Reagent to the biological sample residing in a specific well of a disposable sample plate, and (3) sample washing following the staining or release incubation step.

#### Microscope

All microscope images on the MACSima System are obtained using an epifluorescence setup with one of three different objectives: 20 × objective (NA 0.75, 170-micron coverslip glass), long-working-distance 20 × objective (NA 0.45, objective slides 1.0 mm), and 2 × (NA 0.1). Fluorescence excitation is achieved with custom-designed illumination based on a set of LEDs (infrared, red, green, blue, UV) with LED-specific filters to narrow the excitation light spectrum. Additional excitation/dichroic/emission filter sets define the epifluorescence channels optimized for standard dyes (e.g. DAPI, FITC, PE, APC, and Vio 780). Images are captured by a monochromatic scientific CMOS (SCMOS) camera with 106 nm/pixel for the 20 × objectives and 1060 nm/pixel for the 2 × objective. Autofocusing is achieved in two ways. A hardware autofocus measures the position on the glass surface using infrared light to a precision of < 1 µm. Image-based autofocusing is also possible via optimization of the DAPI image.

#### Photobleaching

Photobleaching is achieved by focusing an additional set of red, green, and blue LEDs onto a single square spot (3 mm × 3 mm). Illumination with the blue LED (2 W/cm^2^ at the sample; 2 min), green LED (0.4 W/cm^2^ at the sample; 2 min) or red LED (1 W/cm^2^ at the sample; 6 min) generates a > 90% reduction of the FITC, PE or APC intensity.

#### Stage

Reagent plates and sample holders are mounted on an xy-stage (with positional accuracy on the order of a few µm) to align the biological sample with the optical path of the microscope or with the photobleaching position, as well as to bring the reagents and sample to the needle position.

#### Image acquisition and processing

The image processing pipeline for the MACSima System is displayed in Fig. S2. In the first step, a series of single-channel exposures of a fixed position in the sample are combined into a single, statistically optimal, high dynamic range (HDR) image based on a calibrated Gaussian noise model for the IRIS 15 sCMOS camera. Our use of an HDR representation for the images allows for the removal and replacement of all saturated pixels and additionally boosts the signal-to-noise ratio in the dimmer portions of the image. In the next steps, the HDR image is corrected in each pixel for the sensor flatfield (pixel-to-pixel differences in quantum efficiency), the optical profile (illumination/detection gradient across the field of view), and hot/cold outlier pixels (by median filtering). Non-local corrections are then performed over the image to correct for distortion (including chromatic effects), to register the images over the cycles, to stitch neighboring (overlapping) images together, and to downsample to the Nyquist frequency. Spectral unmixing of the image (along with corresponding images obtained for the other channels) is then performed based on a calibrated crosstalk matrix. A final subtraction of the pre-stain image removes any residual intensities from remaining autofluorescence or incomplete erasure of the previous cycle staining. While not all images presented in this publication were analyzed with the full pipeline as described above, the most critical processing steps corresponding to the removal of saturated pixels, replacement of hot/cold pixels, flatfielding, and subtraction of the pre-image were applied to all datasets presented here. Further details of the image processing pipeline will appear in a separate publication.

### Image analysis

Image datasets (stack of images) for each tissue were imported into the MACS iQ View Software. The software uses nuclei and cell membrane markers to perform image segmentation identifying individual cells. As it uses all of the cell membrane markers known to the MACS iQ View Software simultaneously, it can segment most cell types in a sample. For each tissue similar segmentation parameters were used. Once the cells are identified, the features like mean fluorescent intensities (MFI) are computed for each cell against the background. These intensities are then used for further downstream processing. During the downstream analysis, all computed MFI are scaled between the range 0 and 1 for visualization and comparability.

### Bead experiments

Fluorophore-labeled beads were used to compare the linearity and sensitivity of image cytometry by the MACSima System with flow cytometry by the MACSQuant Analyzer in the following way. Compensation beads from the MACS Compensation Bead Kit, anti-REA (130-104-693) were incubated with different stoichiometric mixtures of CD4-APC (130-113-222) and dark CD4-Biotin (130-113-224) antibodies (clone REA623). The anti-REAfinity Antibody conjugated to the bead surface generically recognizes REAfinity Antibodies through their kappa light chains, with the specific CD4-recognition domain here playing no role. Saturation of CD4-APC antibodies on the beads was determined from a titration series to occur at roughly 10 µg/mL (95% saturated). Compensation beads were diluted (1:2) and incubated in the dark (10 min, RT) in MACSQuant Running Buffer (130-092-747) to a final volume of 400 µL with stoichiometric mixtures of CD4-APC to CD4-Biotin antibodies at overall saturating conditions equivalent to the following percentages of labeled probe: 100, 10, 1, 0.33, 0.10, 0.030, 0.010, 0.0030, 0.0010 (see Fig. 2a). Samples were shaken during incubation and diluted afterwards with 1 mL of the buffer to stop the incubation process. The beads were then centrifuged at 300×*g* for 5 min to remove unbound antibodies and washed 1 × in the original volume of 400 µL. Half of the volume of each of the samples was transferred to a well of a glass-bottomed (170 μm coverslip) 24-well plate (~ 1 × 10^6^ beads/well) for image cytometry with the MACSima System and the other half was used for flow cytometry with the MACSQuant Analyzer.

For flow cytometry by the MACSQuant Analyzer, beads were gated in the MACSQuantify Software v2.13.0 using the following gating strategy. (1) Draw an ellipse gate around the densest region of beads in an FSC-A vs. SCC-A density plot (A = area of detection peak, FSC = forward scatter, SSC = side scatter). (2) Draw a polygon gate around the linear fit events in an FSC-A vs. FSC-H plot (H = height of peak) to purify for singlets. (3) Draw an interval gate around the bead distribution in an APC-A histogram plot.

For image cytometry of the beads by the MACSima System, 15 separate positions in each well were imaged (see cropped example image in Fig. 2a inset) with the images spaced far enough apart (by 1 mm) from one another to avoid inter-image photobleaching during acquisition. Several hundred beads were acquired per position with, consequently, several thousand total beads imaged for each well (each assayed percentage, see Fig. 2a). Imaging consisted of an exposure series (60, 160, 640 ms) in the APC channel (total acquisition photobleaching was < 5%) followed by a single exposure (1500 ms) in the DAPI channel of the weak bead autofluorescence, with the DAPI image used to create segmentation masks around each detected bead (the masks were therefore independent of the degree of APC staining).

The brightest unsaturated image in each APC channel image series was processed in the following way: (1) Subtract camera readout offset. (2) Flatfield by dividing by a readout-offset-subtracted image of APC in solution. (3) Divide by exposure time.

For object mask creation, the bead autofluorescence image was analyzed in ImageJ/FIJI as follows: (1) Subtract camera readout noise. (2) Perform Gaussian blur with σ = 3 pixels. (3) Perform “Auto Local Threshold” with “Median” filter and radius = 15 pixels to obtain binary image. (4) From “Morphological Filters”, dilate as a disk with radius = 2 pixels. (5) Fill holes. (6) Perform watershed. (7) Label all particles with area greater than MIN = 4000 and less than MAX = 12,000 pixels, and with circularity greater than 0.1. (8) Save object masks.

To remove background in the APC channel from the object mask pixels, the background was interpolated in the following way: (9) Return to the mask resulting from “(5) Fill holes” above. From “Morphological Filters”, dilate as a disk with radius = 17 pixels and then invert to create a mask for the background. (10) Mask the processed APC channel image to reveal only pure background regions. (11) Using a custom-written Python routine, place a uniform grid (with non-overlapping quadrants of size 400 × 400 pixels) over the background image (5056 × 2960 pixels) to downsample it by performing a median filter over each quadrant with assignment of the median value to the central position of each quadrant. (12) In Python, interpolate over the median values (quadrant centers) to estimate the background contribution to the object mask pixels using “griddata” (OpenCV) with the method “cubic” for bulk pixels followed by the method “nearest” for boundary pixels. (13) In ImageJ, subtract interpolated background image from the initial processed APC channel image. (14) Integrate background-subtracted bead intensities over each object mask and save the results as a table.

### PBMC experiments

To compare the sensitivity of imaging-based cytometry on the MACSima System with flow cytometry on the MACSQuant Analyzer on a real biological sample, PBMCs were examined as follows. A buffy coat from a healthy anonymous donor was obtained from the German Red Cross Dortmund, and the PBMCs were isolated by density gradient centrifugation. The cells were stained with CD45-VioBlue (130-110-637) as a general marker for PBMCs and were additionally stained with different stoichiometric ratios of a fluorescent CD3-APC antibody (130-113-135) and a non-fluorescent CD3-Biotin antibody (130-113-137). More specifically, a 1.5 mL suspension of fresh PBMCs containing 2 × 10^7^ cells/mL was prepared using autoMACS Running Buffer (130-091-221). Furthermore, buffer was added to separate Eppendorf tubes along with CD45-VioBlue (1:50) and mixtures of the CD3-APC and CD3-Biotin antibodies at the stoichiometries listed in Table 1 and at overall high saturation (the standard dilution of 1:50 for the CD3 antibodies implies a high degree of saturation of the targeted CD3 epitopes), considering a 1:3 cell suspension dilution and a final volume of 300 µL. Then, 100 µL of the prepared PBMC suspension was added to the 200 µL contained in each separate tube. Cells were stained in the dark and shaken during incubation (10 min, RT). Afterwards, samples were diluted with 1 mL of buffer to stop the incubation process. The cells were then centrifuged at 300×*g* for 5 min to remove unbound antibodies, resuspended in 200 µL of 4% PFA and fixed for 10 min in the dark. Subsequently, cells were centrifuged at 600×*g* (fixed cells are lighter) for 5 min to remove PFA, and washed 1 × in 300 µL of buffer, with one half (150 µL) used for the MACSima System and the other half for the MACSQuant Analyzer.

For the MACSQuant Analyzer, CD45^+^ cells were gated using density plots according to the following strategy. (1) Draw a polygon gate around the linear fit events in an FSC-A vs. FSC-H plot. (2) Disregard platelets, red blood cells, and debris (low FSC-A signals) with a rectangular gate drawn in an FSC-A vs. SSC-A plot. (3) Select CD45^+^ cells with a rectangular gate in a CD45-VioBlue-A vs. SSC-A plot. Then, assess the selected cells for their CD3-APC-A (integrated) intensities.

For the MACSima Imaging System, 150 µL of the cell suspension from each tube were transferred to a well of a glass-bottomed (170 μm coverslip) 24-well plate and centrifuged at 100 xg for 10 min to attach them to the bottom of the well. Image acquisition was identical to that used for the beads, with the exception that fewer cells were acquired per field. Image analysis was also largely identical to that used for the beads (see “Bead experiments” above), aside from using the CD45-VioBlue staining to create the cell masks instead of autofluorescence, omission of the watershed, and allowance for objects with areas up to MAX = 15,000 pixels. For both flow cytometric and imaging datasets, the individual integrated cell intensities, *I*, were converted by the following formula to an arcsinh scale that is asymptotically equivalent to log_10_, but importantly also allows for negative values:

$$y=\mathrm{arcsinh}(aI - b)/\mathrm{ln}(10),$$

with the normalization factors *a* and *b* independently chosen for each dataset such that the mean (in arcsinh units) of the blank distribution equals 0 and the mean of the highest stained population equals 2 (see Fig. 2c).

### Gene design and generation of CAR plasmids

Gene synthesis in combination with an optimization algorithm for codon usage in humans (ATUM, Newark, CA) was used to design genes of interest. *E. coli* DH5alpha were used for cloning and plasmid generation. Plasmids were purified using DNA isolation kits (Qiagen).

Plasmids encoding the CAR construct were prepared using standard molecular biology and cloning techniques. The CAR is designed with a single-chain variable fragments (scFvs) specific for Biotin, a IgG4hinge, a CD8α transmembrane domain, CD28 costimulatory and the 4-1BB/CD3ζ-derived intracellular signaling domain. The CAR sequence was linked to a P2A sequence to induce co-expression of truncated low-affinity nerve growth factor receptor (LNGFR).

### Lentiviral vector production

Second-generation, self-inactivating VSV-G-pseudotyped lentiviral vectors were produced by transient transfection into adherent HEK293T cells. One day before transfection, 1.6 × 10^7^ HEK293T cells were seeded per T175 flask. Each T175 flask was then transfected with a total of 35 µg plasmid DNA composed of pMDG2 (encoding VSV-G), pCMVdR8.74 (encoding gag/pol), and the respective transgene-encoding transfer vector using MACSfectin Reagent (Miltenyi Biotec). All transfection reactions were performed with a DNA:MACSfectin Reagent ratio of 1:2. Following overnight incubation, sodium butyrate (Sigma-Aldrich) was supplemented at a final concentration of 10 mM. After 48 h the medium was collected, cleared by centrifugation at 300×*g* and 4 °C for 5 min and filtered through PVDF filters with a pore size of 0.45 µm. Concentration of the viral stock was performed by centrifugation at 4 °C and 4000×*g* for 24 h. Pellets containing lentivirus were air-dried and resuspended at a 100-fold concentration with 4 °C cold PBS. Lentiviral aliquots were stored at − 80 °C. In order to generate EPCAM-positive A2780 cells, human EPCAM lacking intracellular domains was cloned in the transfer vector. To generate eGFP-positive A2780 cells, a cassette containing eGFP was cloned in the transfer vector.

### Generation of knock-out cell lines

A2780 THY1 knockout cells were generated in-house by CRISPR/Cas9-mediated knockout and subcloning. The respective THY1 gRNA used was GACGAAGGCTCTGGTCCACT. Ribonucleoprotein particle complexes were assembled according to the manufacturer’s protocol (IDT). In brief, CRISPR-Cas9 gRNA was incubated with CRISPR-Cas9 crRNA at a 1:1 ratio for 15 min at ambient temperature to form a gRNA complex. In a second step the gRNA complex was incubated with Cas9 nuclease at a 1:1 ratio for 10 min at ambient temperature to form the ribonucleoprotein particle complexes. Subsequently, A2780 cells were resuspended at a density of 10^5^ cells/mL in 40 µL electroporation buffer (Miltenyi Biotec) including 2 µL ribonucleoprotein particle complexes (equals 2.7 µg ribonucleoprotein particle complexes). After mixing the suspension cells were transferred to an electroporation cuvette (Miltenyi Biotec) and electroporated using the CliniMACS Electroporator (Miltenyi Biotec). Thereafter cells were cultured under standard conditions and THY1 knockout cells were sorted with the MACSQuant Tyto Cell Sorter (Miltenyi Biotec). Knockout efficiency was monitored by flow cytometry to assess THY1 surface expression.

### Isolation of T cells and generation of CAR T cells

PBMCs were isolated from buffy coats or leukapheresis products by low-density centrifugation on Pancoll (Pan-Biotech, Aidenbach, Germany) and enriched for Pan T cells by negative magnetic selection (Miltenyi Biotec). The enriched Pan T cells were resuspended at a density of 1 × 10^6^ cells/mL in TexMACS Medium containing 12.5 ng/mL recombinant IL-7 and 12.5 ng/mL recombinant IL-15 and stimulated with T Cell TransAct (all from Miltenyi Biotec). One day after activation, T cells were lentivirally transduced with a multiplicity of infection (MOI) of 1:5 with the respective concentrated vector. Cell numbers were determined every 2–3 days and fresh TexMACS Medium supplemented with 12.5 ng/mL recombinant IL-7 and 12.5 ng/mL recombinant IL- 15 was added to maintain a cell concentration of 1 × 10^6^ cells/mL until day 12. Analysis of CAR-expressing cells was routinely performed on day 6 after activation using flow cytometry, and human low-affinity nerve growth factor receptor (LNGFR) marker expression was detected via anti-human LNGFR antibodies. Afterwards, cells were expanded in the presence of 12.5 ng/mL recombinant IL-7 and 12.5 ng/mL recombinant IL-15 until downstream processing.

### CAR T cell killing assays

All functionality assays were performed with fresh CAR T cell populations on day 12–14 following activation with T Cell TransAct and in TexMACS Medium without additives. The frequency of CAR-expressing T cells was equalized before all functional assays and untransduced T cells served as a control for allogeneic reactivity. Target cells were harvested using trypsin (3 min, 37 °C) and cell numbers as well as target expression (based on THY1 REA897-APC, EPCAM REA764-VioBlue; staining was performed according to the manufacturer’s protocol) were determined on a MACSQuant Analyzer 10. Afterwards, target cells were seeded to ensure proper target cell adherence. To analyze CAR T cell-mediated cytotoxicity by target cell death, real-time monitoring using the IncuCyte S3 system (Essen BioScience, Ann Arbor, MI) was performed.

Assays were set up in duplicates by seeding 25,000 GFP-transgenic target cells per well of flat-bottomed 96 well plates and—following overnight incubation—adding CAR T cells to the cultures at a 1:2 (effector:target) ratio. Subsequently, adapter molecules (THY1 REA897-Biotin, EPCAM REA764-Biotin) were supplemented at varying doses. Cultures of target cells only, target cells with mock-transduced T cells, and target cells with Adapter CAR T cells without adapter molecule or non-specific adapter molecule were used as controls. Cytotoxicity was measured as a decrease in green surface area. The surface area at the start of the experiment was considered 100% and the following decrease or increase in surface area was set into relation. Phase contrast and green fluorescence images were captured with 10 × magnification every two hours for 3–6 days. Analysis of images was performed using the software provided by the manufacturer.

### Ethics

For all studies using human primary tissue from glioblastoma, ovarian or pancreatic cancer, written informed consent was obtained following the guidelines of the approved Universitätsmedizin Göttingen, and Cologne Review Board protocol, respectively.

PBMCs were isolated from buffy coats of healthy anonymous volunteers. Buffy coats were purchased from the German Red Cross Dortmund. All blood samples were handled following the required ethical and safety procedures.

All animal experiments were approved by the Governmental Review Committee on Animal Care in NRW, Germany and performed according to guidelines and regulations. Animals were held under specific pathogen-free conditions according to the recommendations of the Federation of European Laboratory Animal Science Association. All procedures were carried out in accordance with the European Communities Council Directive European Communities Council (86/609/EEC) and (2010/63/EU).

Healthy whole blood samples were taken from volunteers who had given their written consent beforehand.

## Supplementary Information

Supplementary Information.
