# Supplementary material for: MACSima imaging cyclic staining (MICS) technology reveals combinatorial target pairs for CAR T cell treatment of solid tumors
Source: Sci Rep. 2022 Feb 3;12:1911. doi: 10.1038/s41598-022-05841-4 (PMC8813936; doi:10.1038/s41598-022-05841-4)
Supplement: Supplementary file 1 — Supplementary Information. [file 41598_2022_5841_MOESM1_ESM.docx]

# Supplementary Information


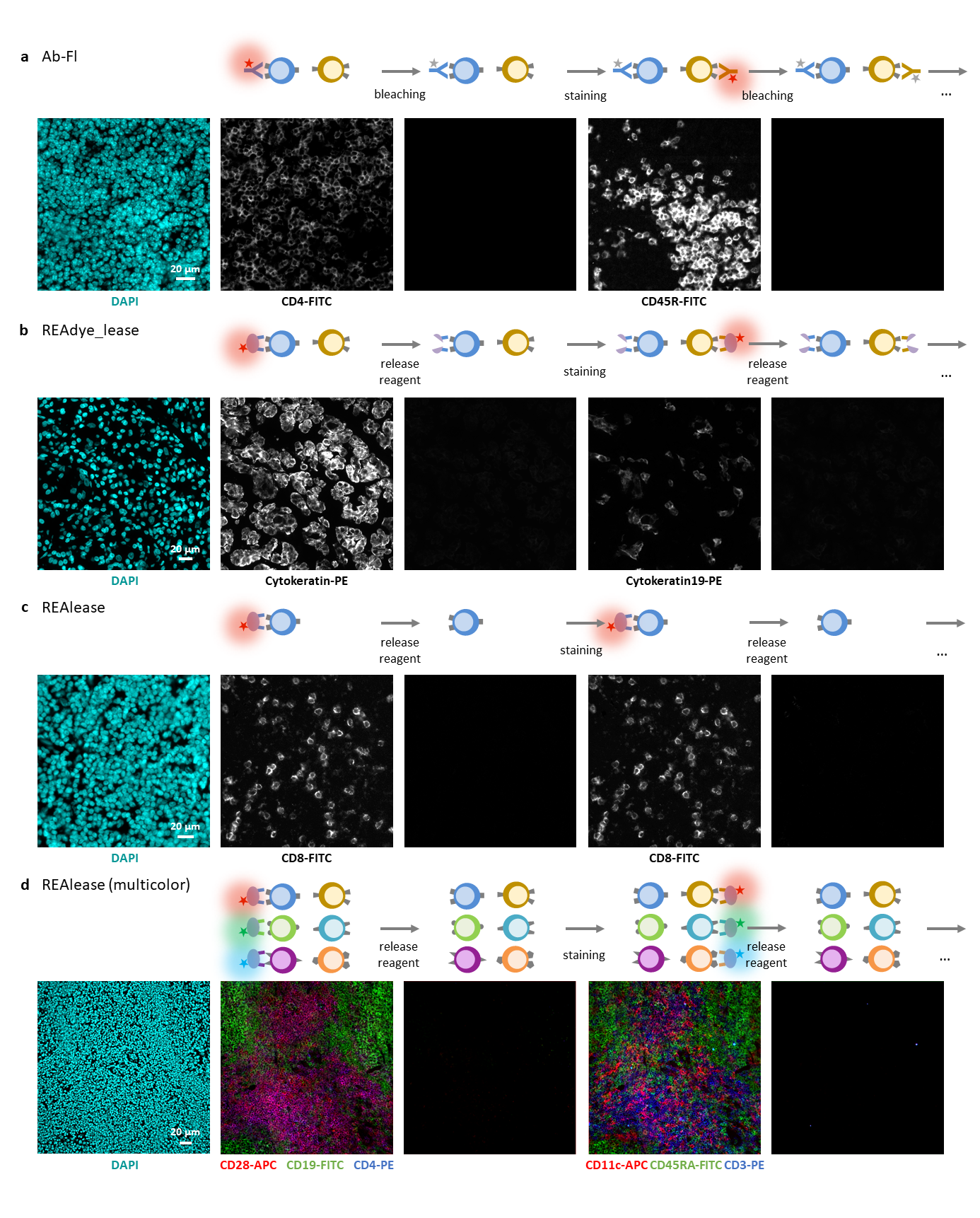


**Supplementary Fig. S1.** **Different erasure processes possible with the MACSima Imaging Platform.**

For each panel, the DAPI image is displayed first (cyan) followed by two consecutive cycles of staining and erasing (with the latter images all displayed at the exact same contrast). (a) Photobleaching. Acetone-fixed mouse lymph node slice stained with CD4-FITC, photobleached, stained with CD45R-FITC, and then photobleached again. (b) REAdye_lease. PFA-fixed human pancreas slice was stained with Cytokeratin-PE and then treated enzymatically to release the fluorophore PE from the antibody-like REAdye_lease Probes. Sample was then stained for cytokeratin19-PE, and the fluorescence erased in the same fashion. (c) REAlease. Acetone-fixed human tonsil slice was stained with REAlease probe CD8-FITC and then treated enzymatically, which disassembled the probe, this time removing not only the fluorophore but also the binding regions. To demonstrate the complete removal of the probe, the sample was restained with the same CD8-FITC probe in the next cycle, yielding a stained image with comparable intensity. The probe was then removed again in the same fashion. (d) REAlease (multicolor). Acetone-fixed human tonsil slice was stained with a multicolor panel of REAlease Probes (CD28-APC, CD19-FITC, CD4-PE) in the first round of staining. All probes were then completely released enzymatically (as in c). The sample was then stained with a new multicolor panel (CD11c-APC, CD45RA-FITC, CD3-PE), which was then released identically.


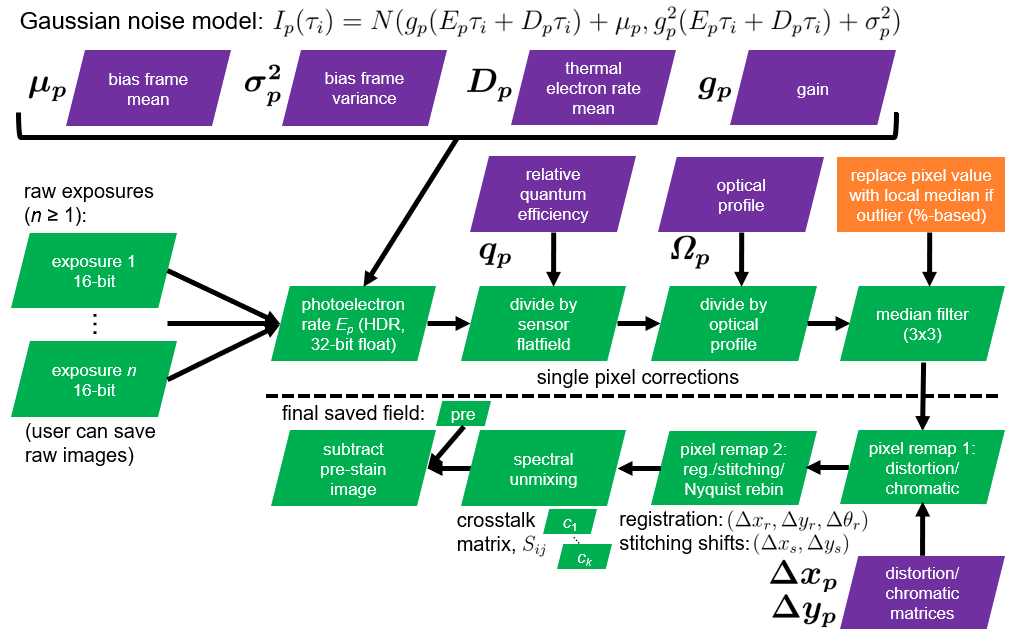


**Supplementary Fig. S2. Image processing pipeline for the MACSima Imaging Platform.**

The image processing pipeline combines multiple single-channel input images (with different exposure times) of a sample field to construct a high-dynamic range (HDR) image, with single-pixel fitting based on a calibrated Gaussian noise model for the IRIS 15 s-CMOS camera. Individual pixels are additionally flatfield corrected (sensor flatfield, optical profile) and median filtered (hot and cold pixel removal). An initial pixel remapping is then performed to correct for distortion and chromatic effects. A second pixel remapping combines corrections for registration (to the initial cycle DAPI image), stitching of neighboring fields, and Nyquist rebinning. Spectral unmixing and subtraction of the pre-stain image yield the final, fully processed and stitched image for each staining.


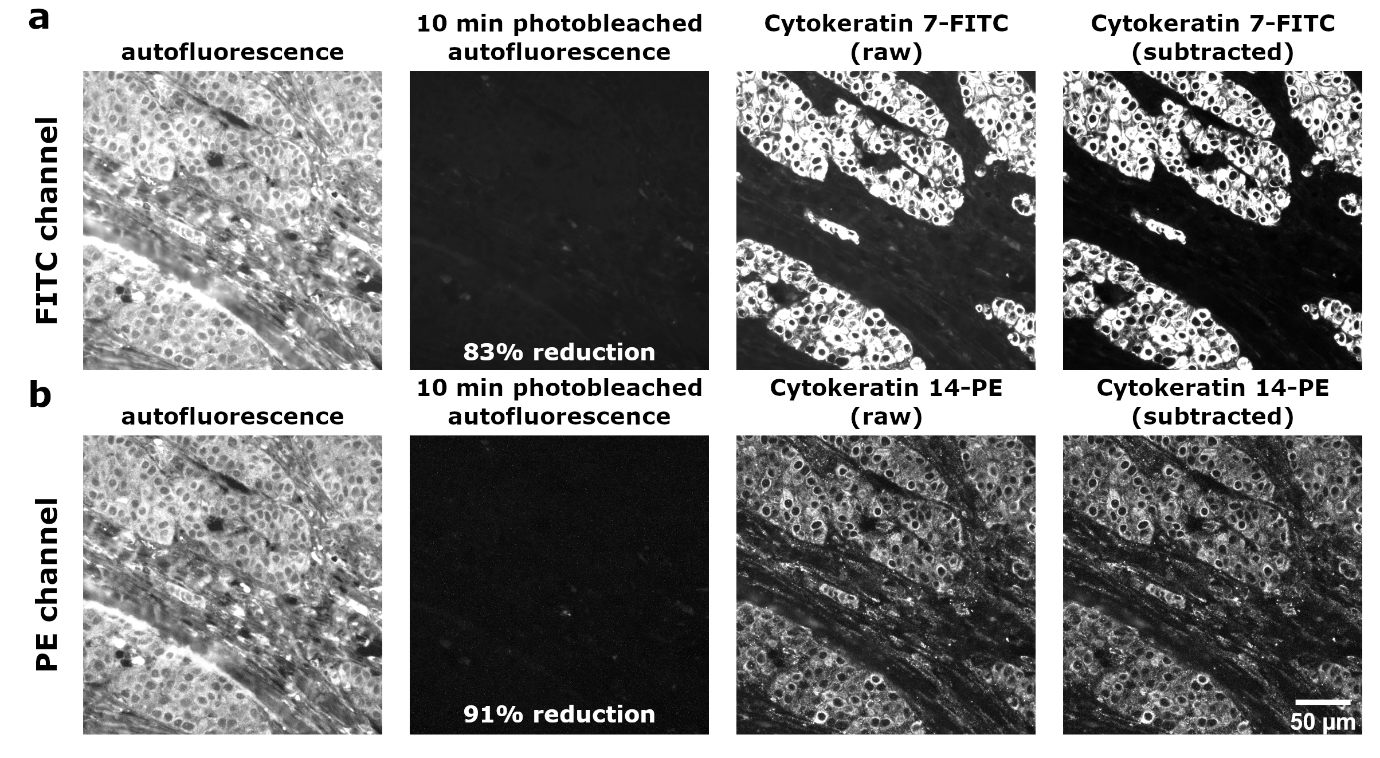
**Supplementary Fig. S3. Photobleaching and subtraction of residual autofluorescence for an FFPE tissue slice.**

(a) In the first image, the initial autofluorescence in the FITC channel for an FFPE slice of human breast cancer (3 µm section) is shown. In the second image, the residual autofluorescence is shown after 10 min of photobleaching, resulting in a reduction of autofluorescence by 83%. In the third image, the first staining image (raw data) in the FITC channel for the MICS run is shown. In the final image, the second image is subtracted from the third image. Note the removal of autofluorescence residuals in the unstained portion of the tissue. All images were contrasted identically with some saturation allowed in order to better display the residual autofluorescence following photobleaching and its removal. (b) The same as in a, but for the PE channel. Photobleaching for 10 min resulted in reduction of autofluorescence by 91%, after which the autofluorescence is barely detectable. All images were contrasted identically.


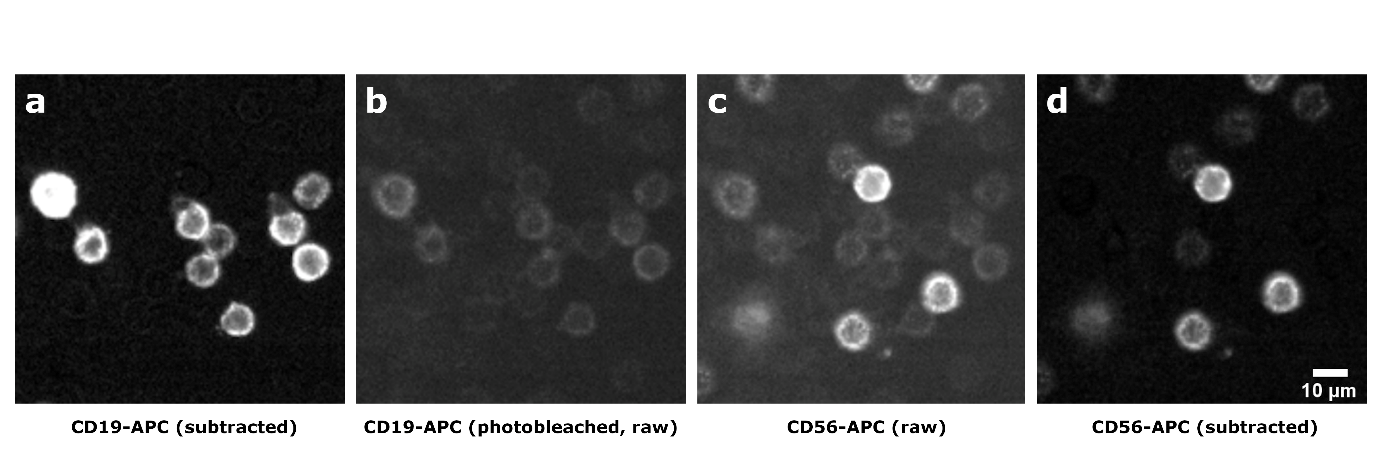
**Supplementary Fig. S4. Efficient subtraction of residual signal due to incomplete erasure of the previous staining.**

(a) PBMCs stained with CD19-APC. Displayed image is subtracted from the previous staining. (b) After photobleaching of the cells shown in a, the specific signal was reduced to a few percent. Displayed image is the raw image. (c) Next cycle staining with CD56-APC resulted in the displayed raw (unsubtracted) image. (d) Subtraction of the photobleached image in b from the next stained image in c efficiently removes the residual staining from the previous cycle, leaving only the specific CD56-APC staining signal. All images are displayed with the same contrast settings.


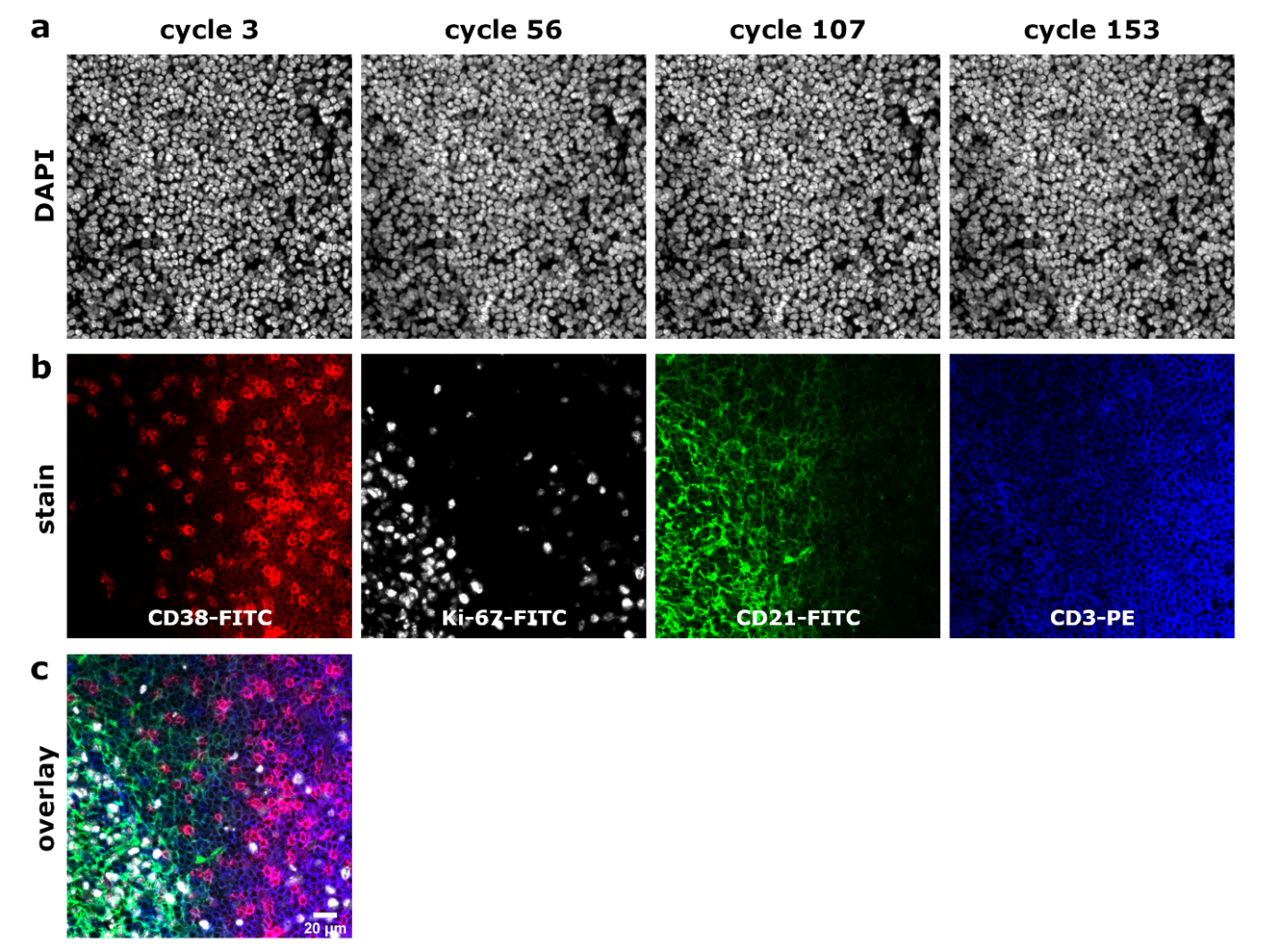
**Supplementary Fig. S5. Example of a MICS run using a total of 327 markers on a PFA-fixed human tonsil slice.**

(a) Preservation of overall tissue morphology (8 µm section) throughout the MICS run is demonstrated by the highly similar DAPI images obtained at cycles 3, 56, 107, and 153 (last cycle) for a three-color MICS run (FITC, PE, and APC) consisting of a total of 327 antibody markers. (b) Representative stain images corresponding to the DAPI images in a. (c) Overlay image of all four images in b.


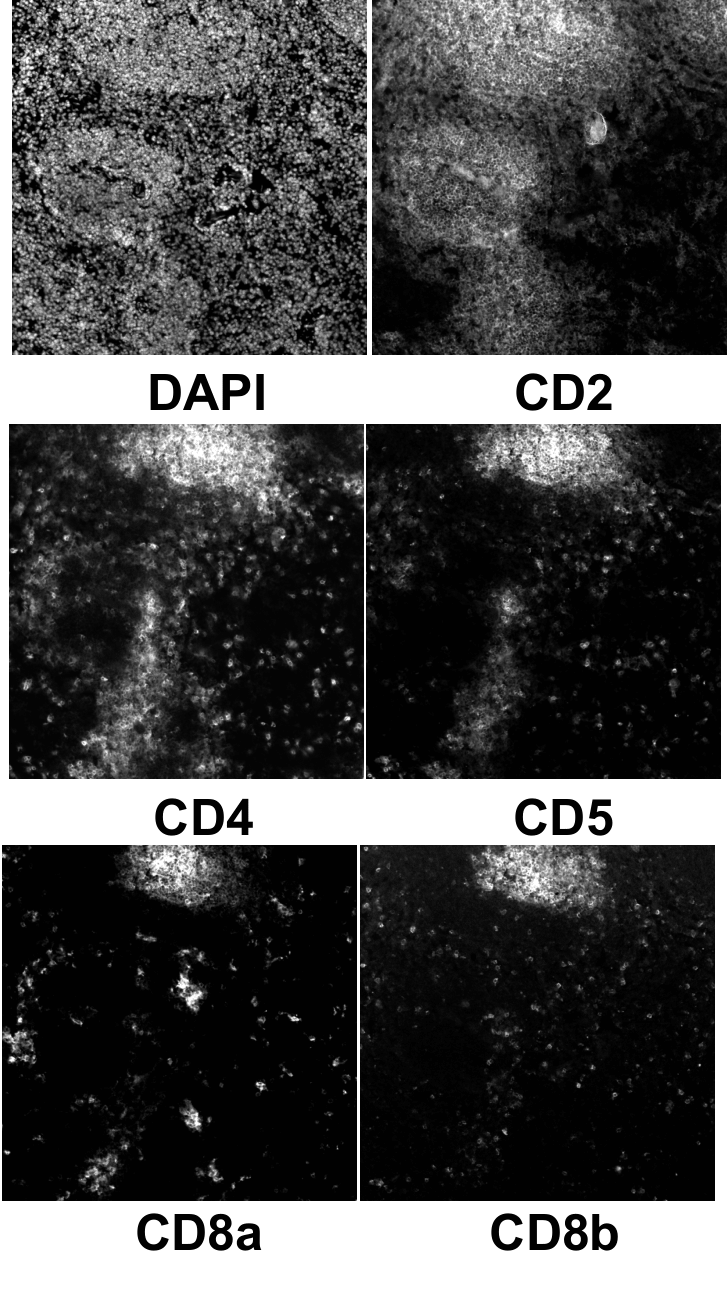

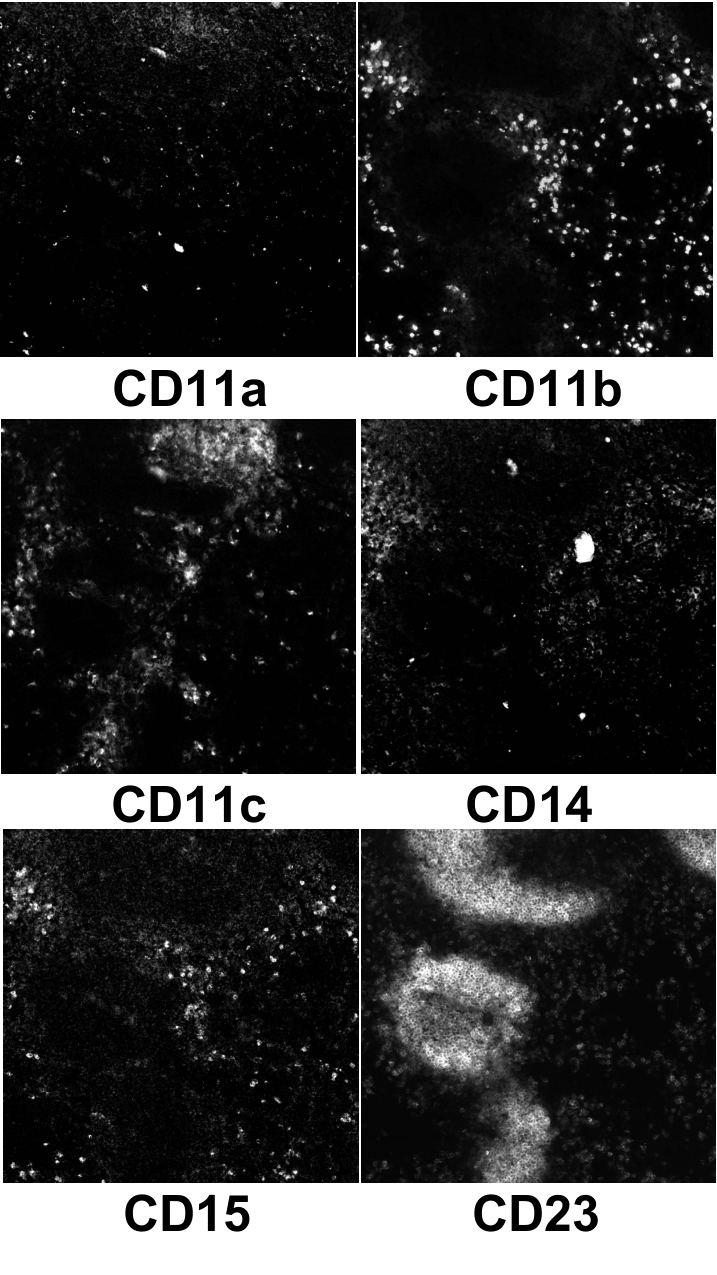


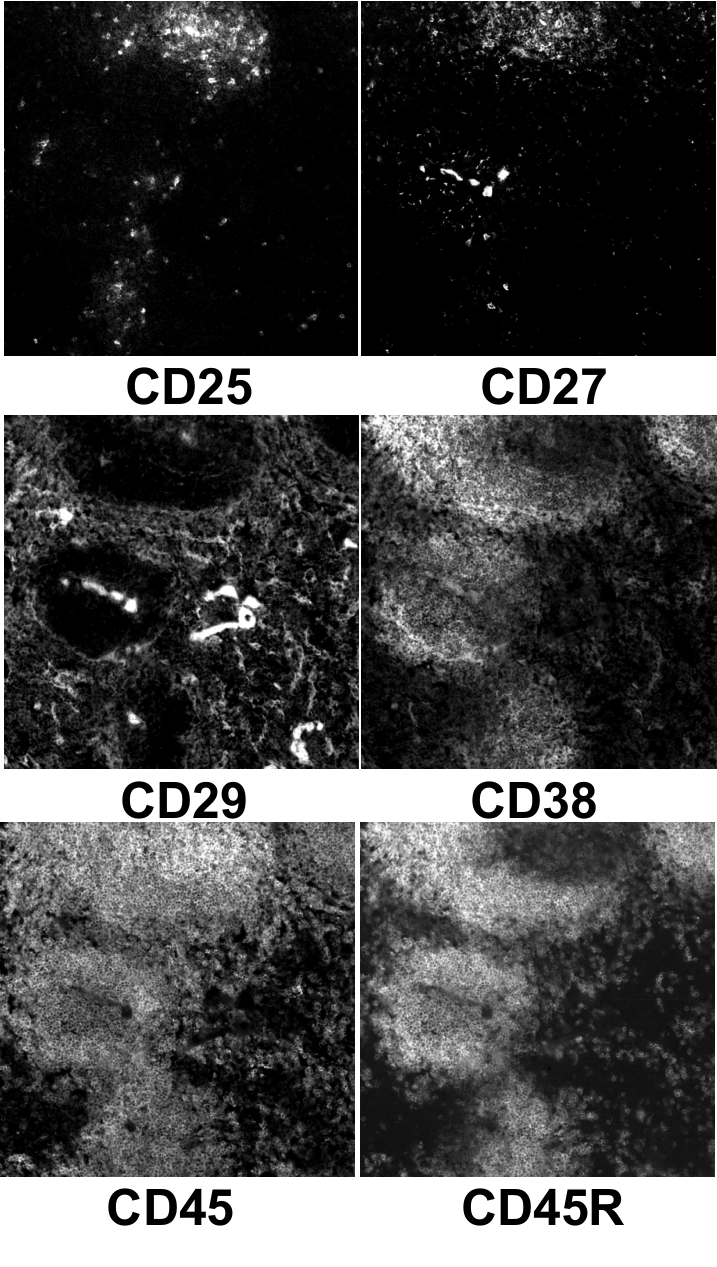

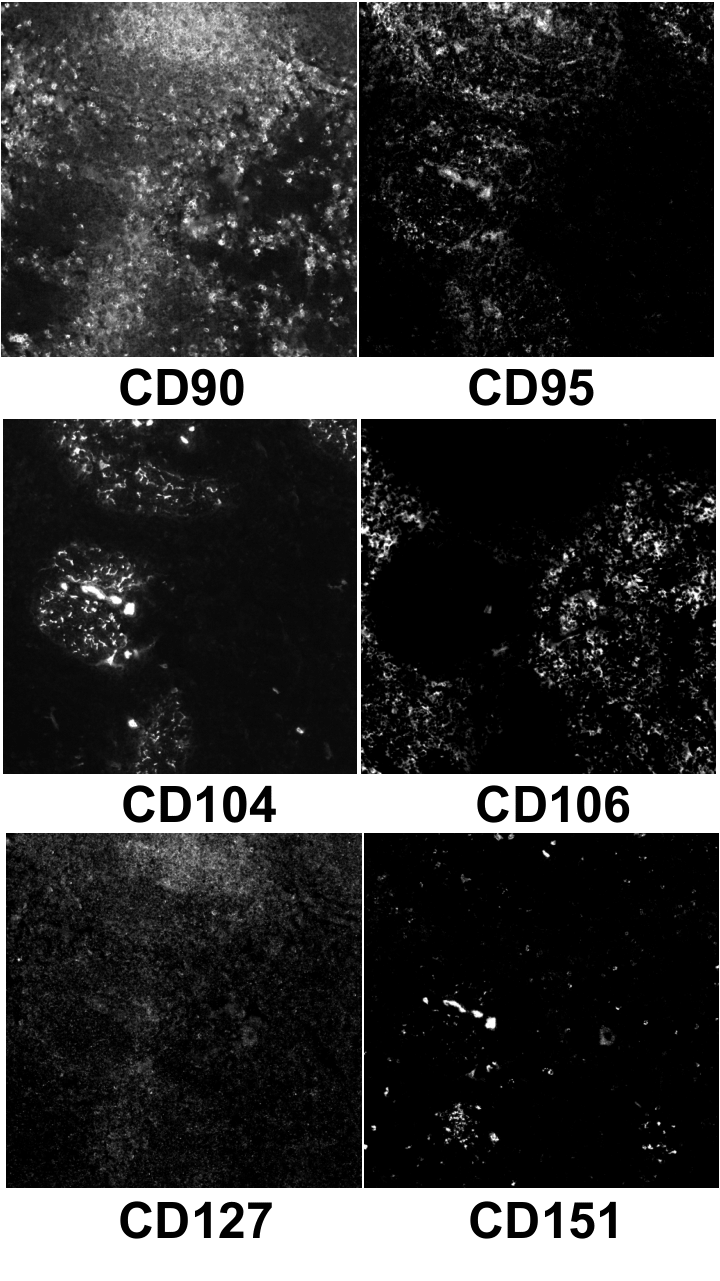


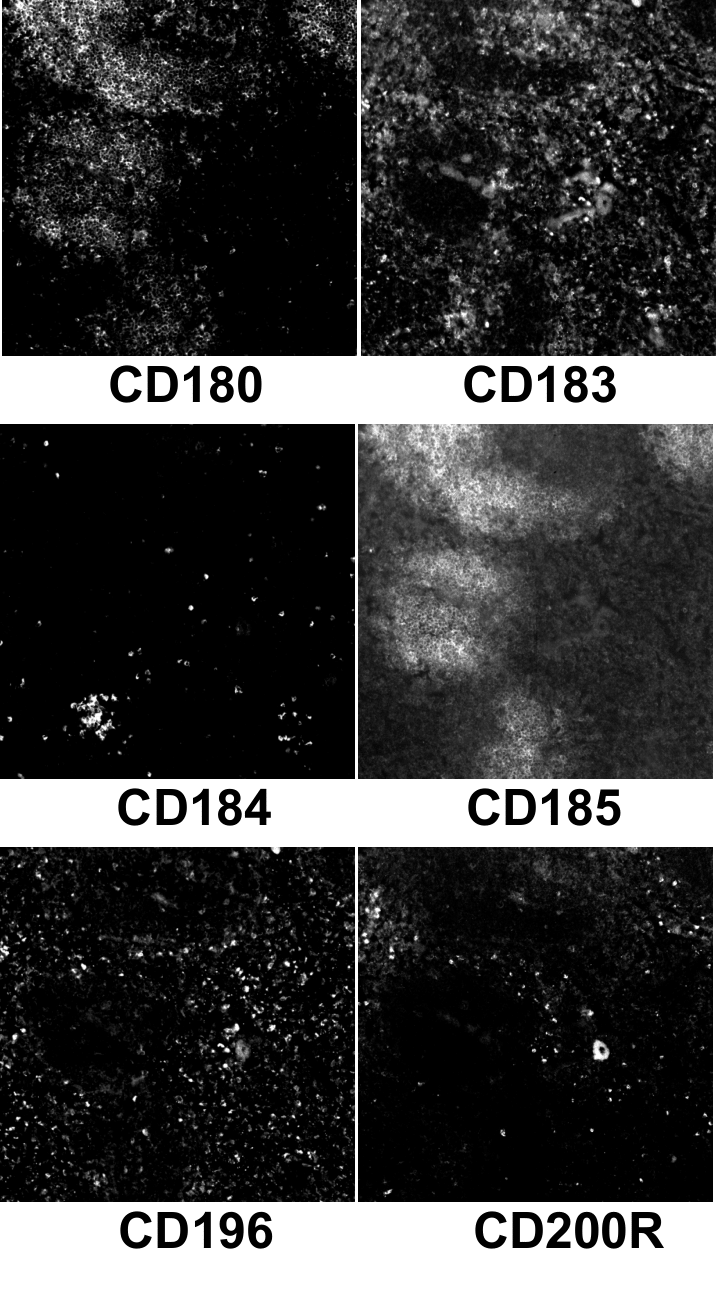


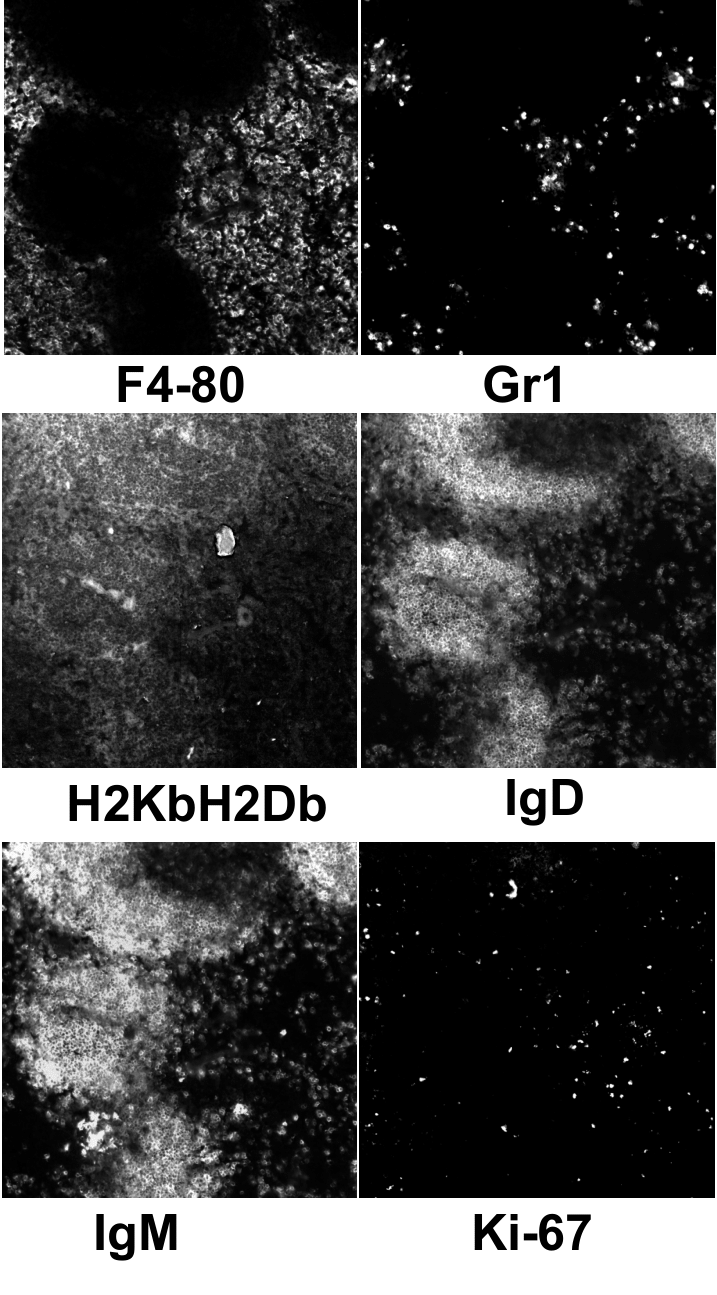

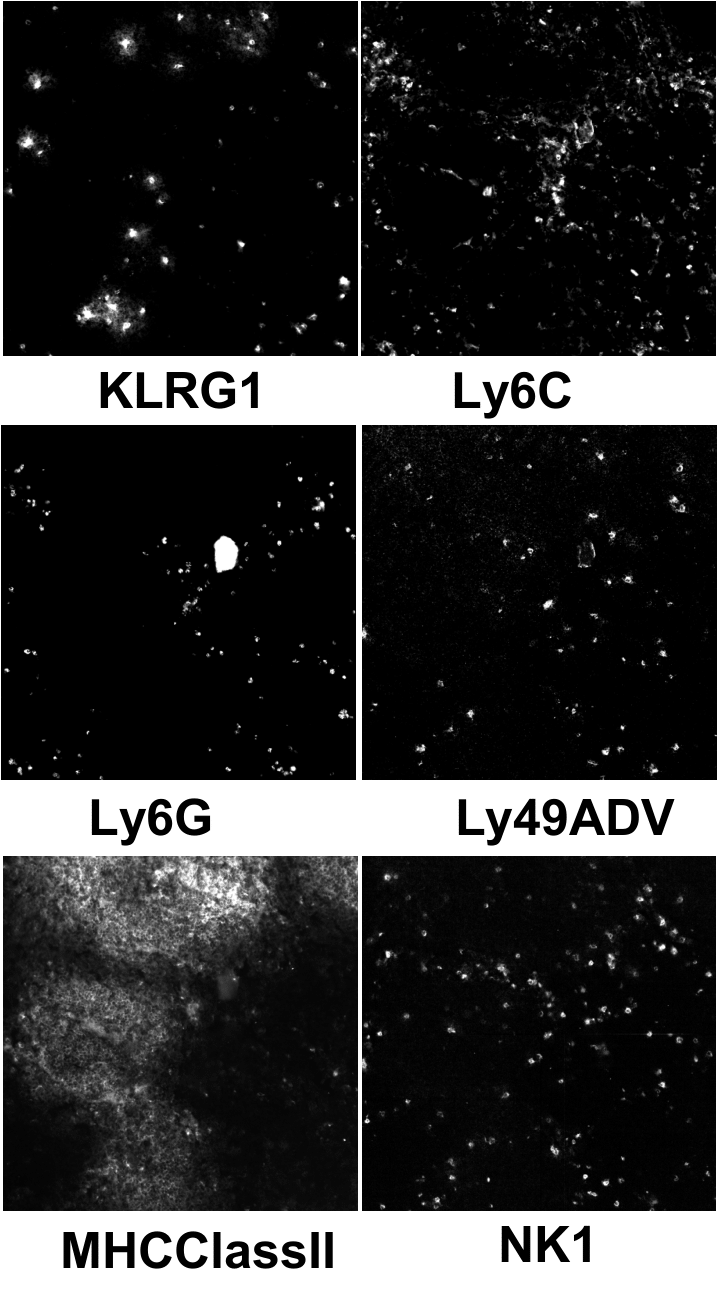

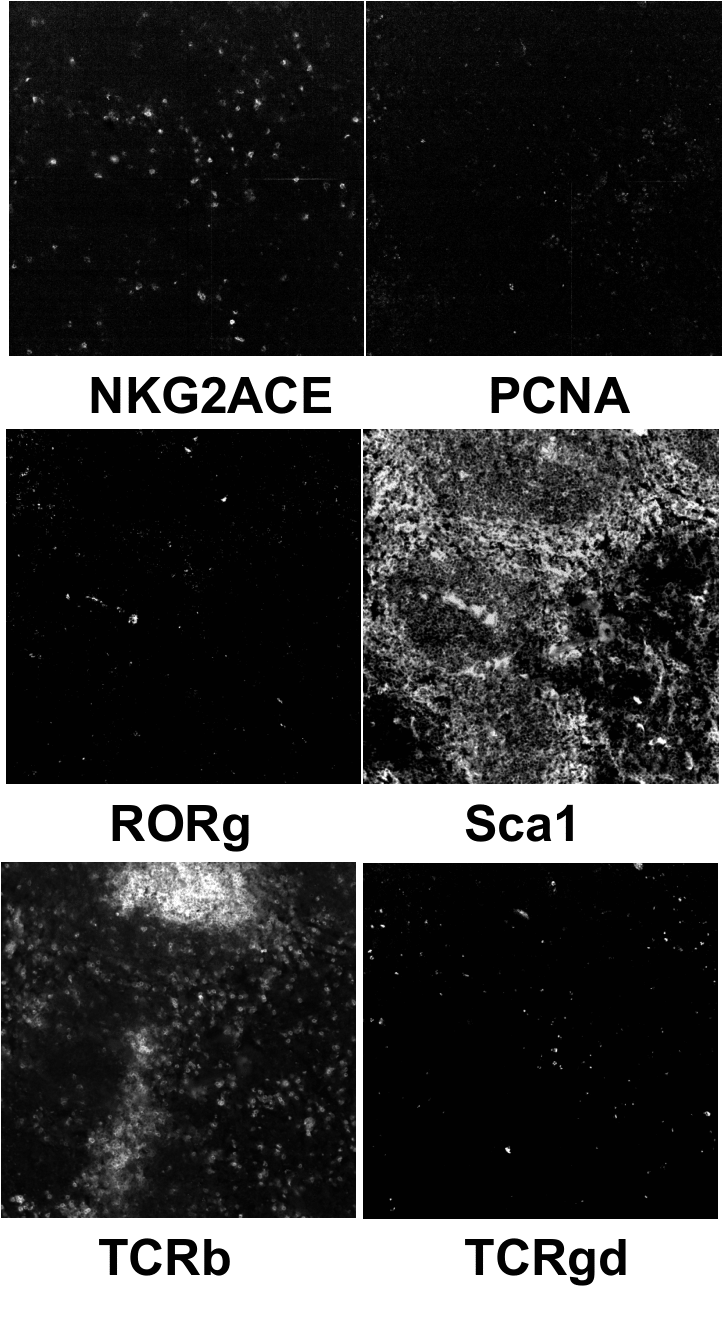


**Supplementary Fig. S6. Higher resolution of the spleen images shown in Figure 3a.**

As it is hard to see details in the images of Figure 3a, a higher resolution set of the images is shown in this supplement. The original images have a dimension of 2024×2024 pixels each.


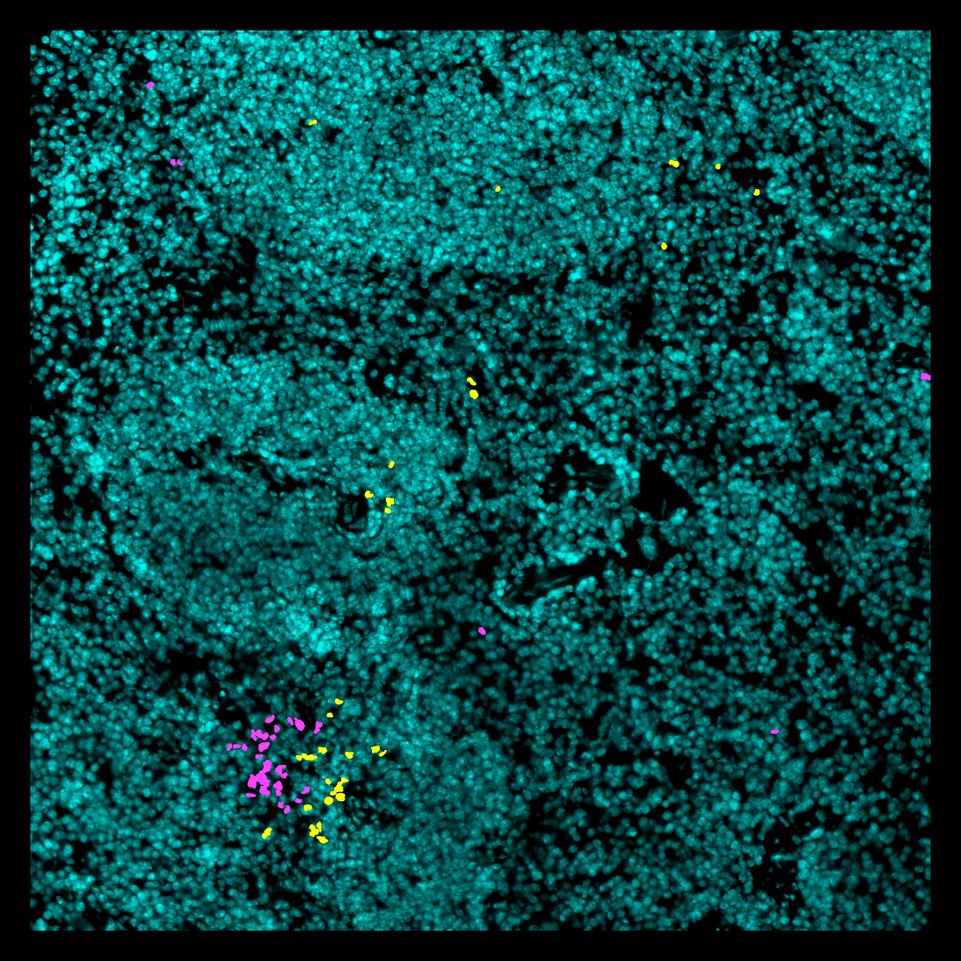


**Supplementary Fig. S7. Rare-cell detection using the MACSima Imaging Platform.**

The blue staining shows the nuclei of the spleen section stained with DAPI. The yellow dots depict a rare subset of T cells that express CD8 alpha but not CD8 beta chains. In this cell population of 11996 segmented cells, 38 rare T cells were identified. The pink dots represent IgM plasma cells. In this section, we found 40 plasma cells.


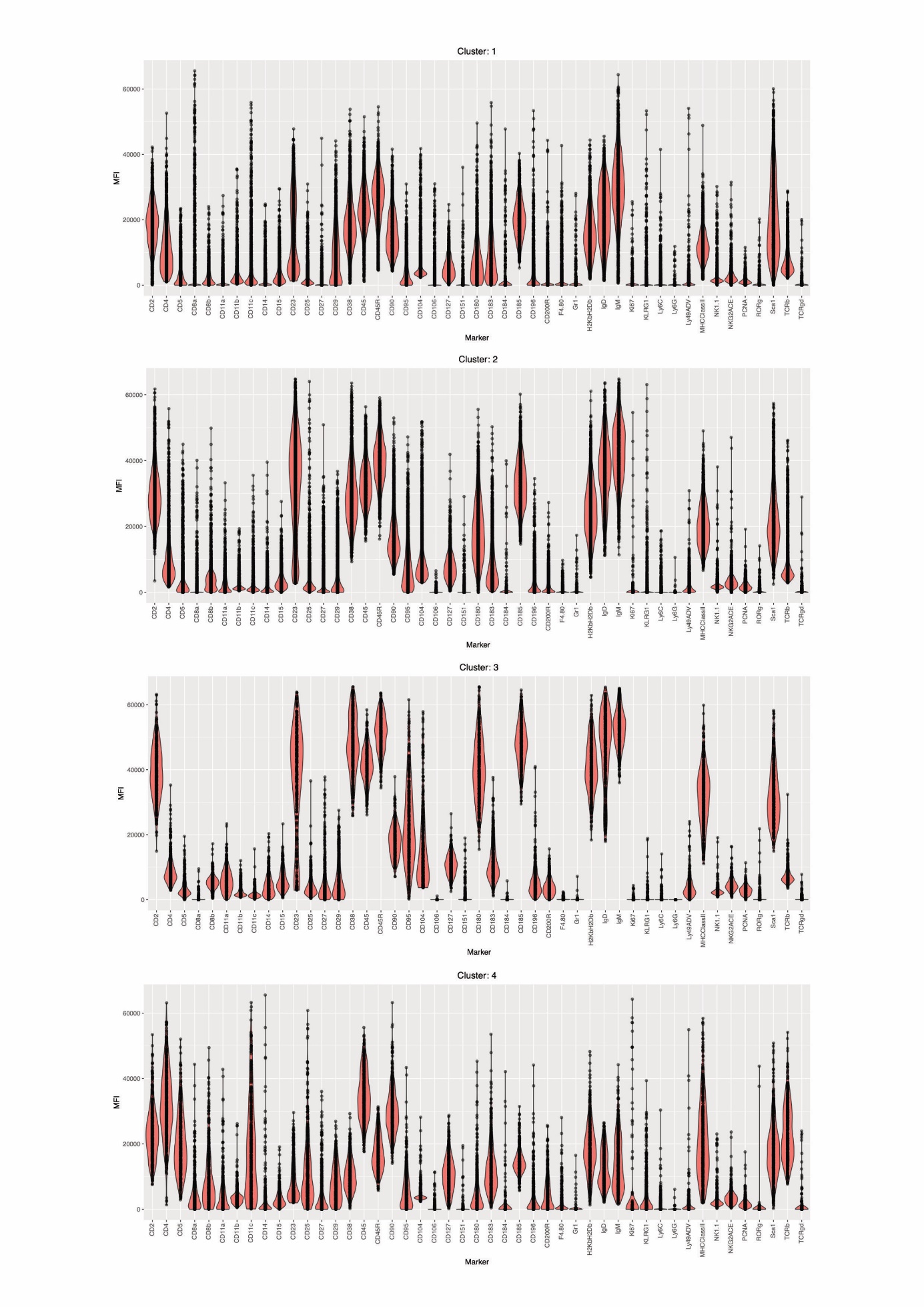


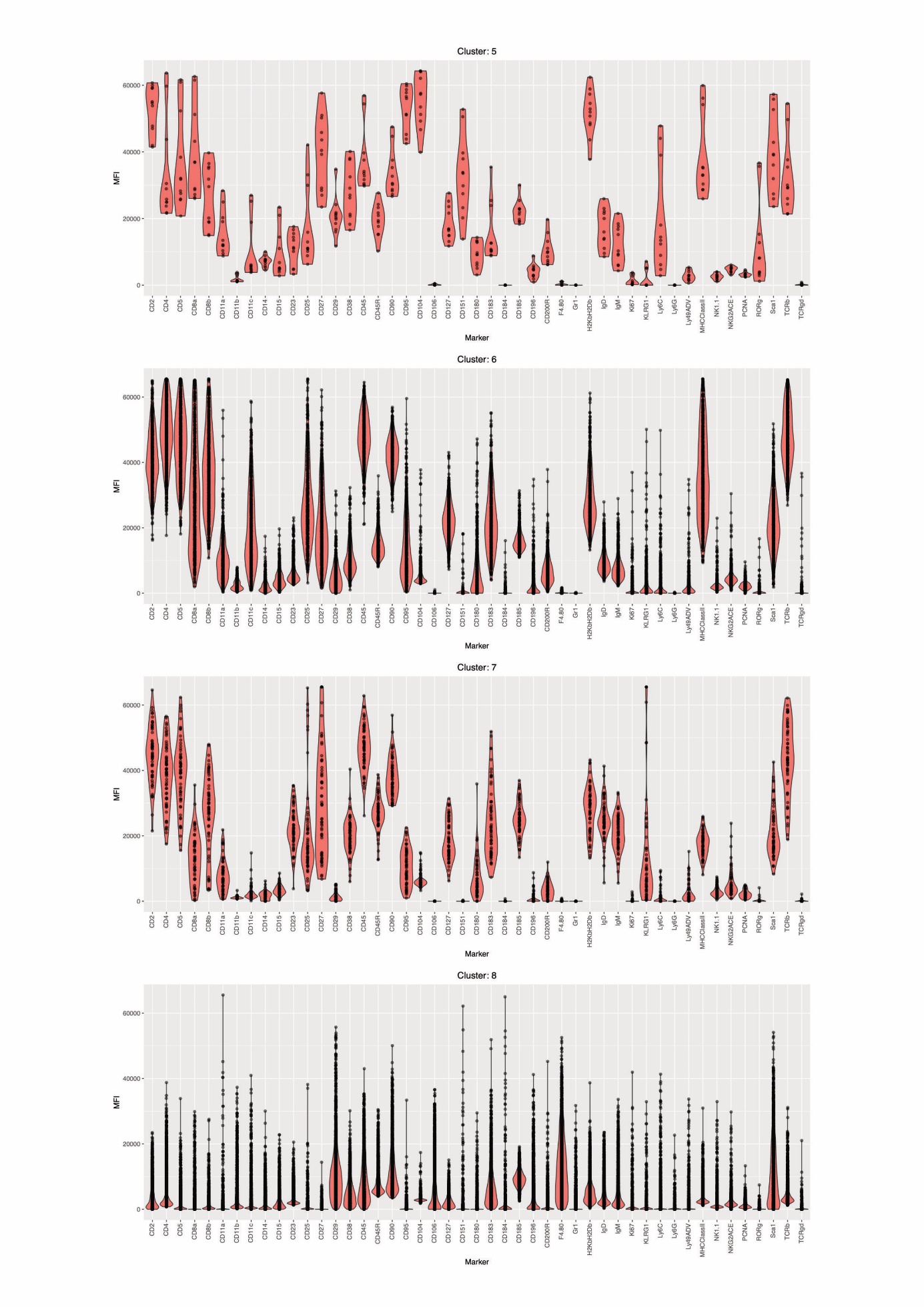


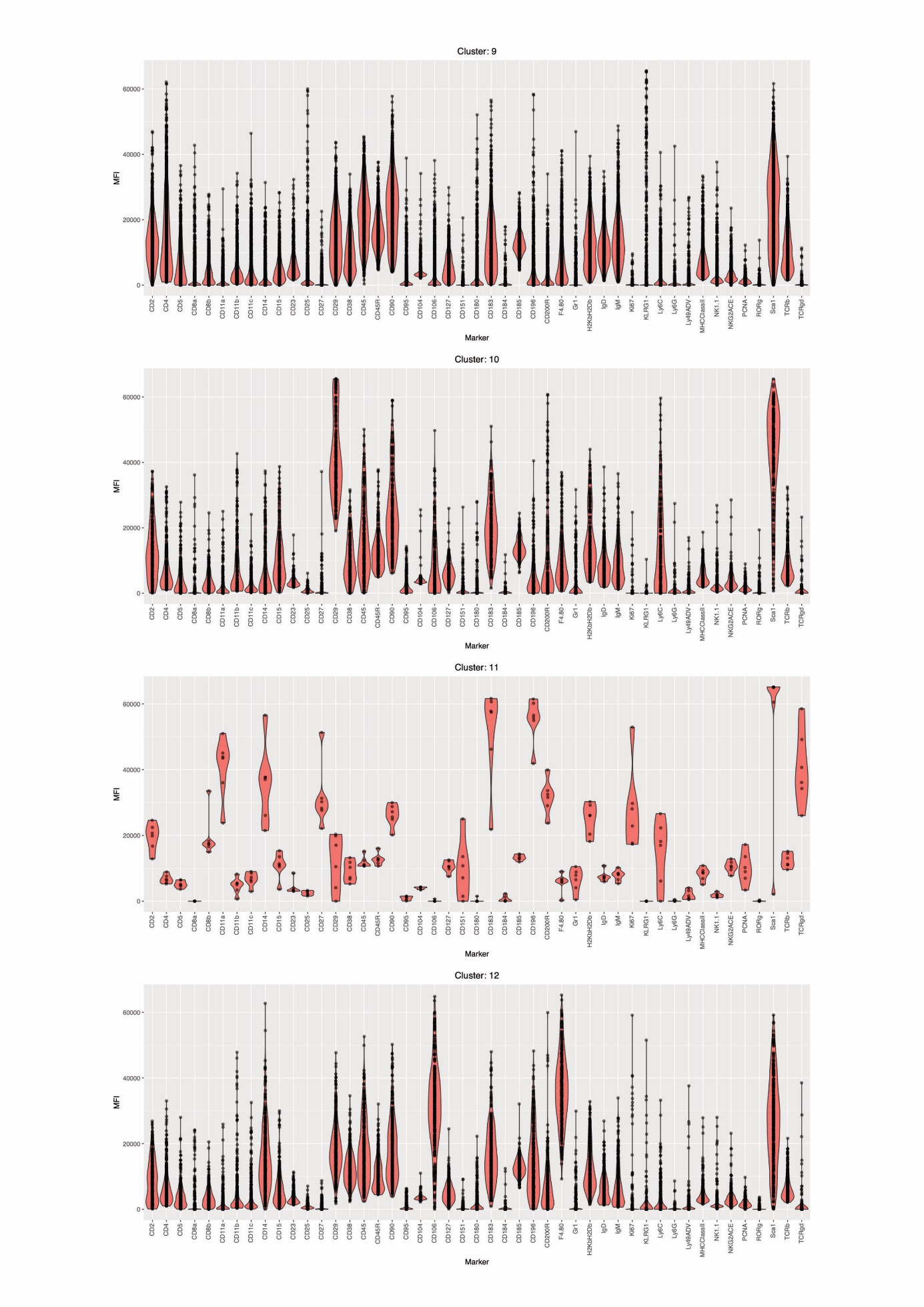


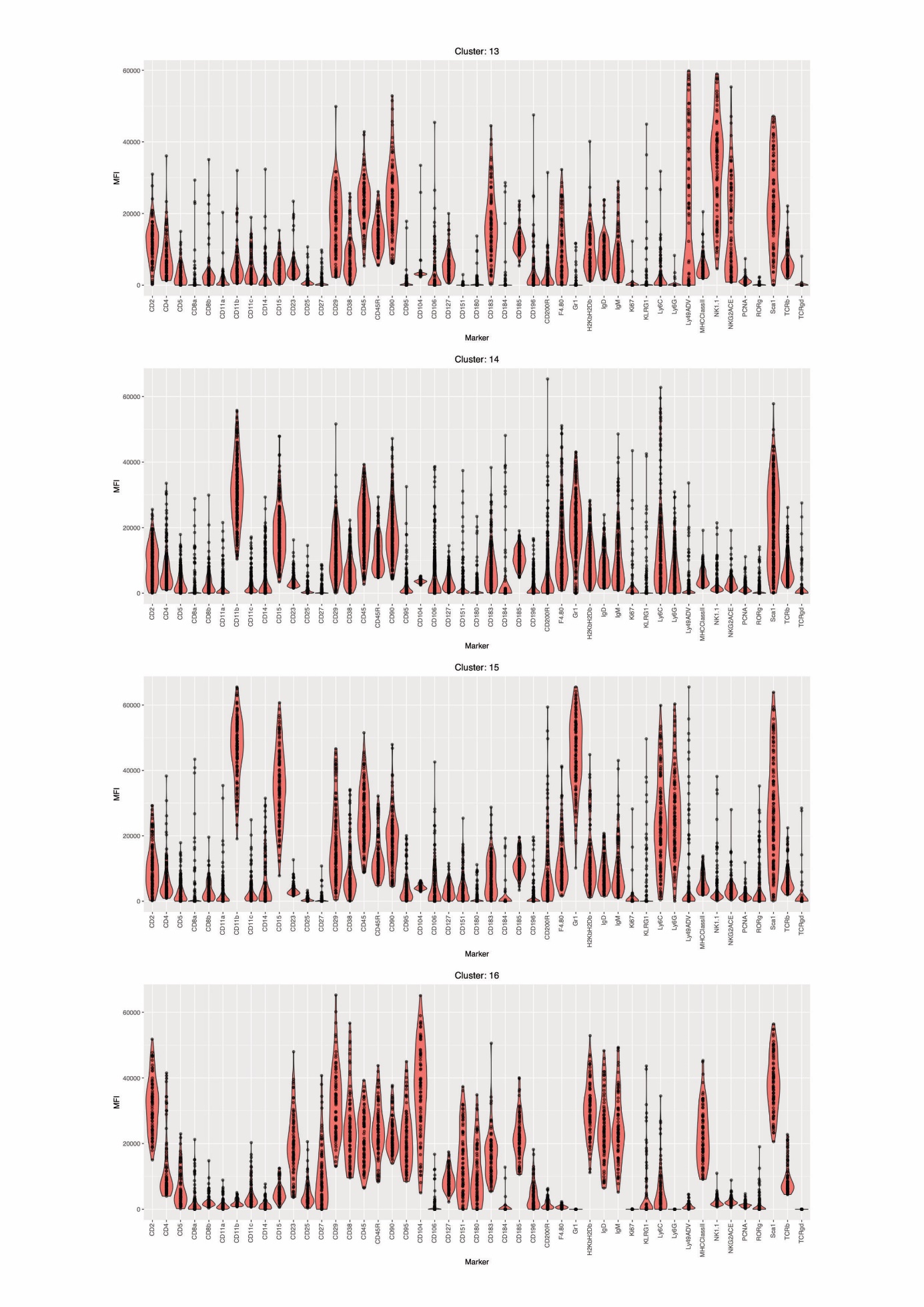


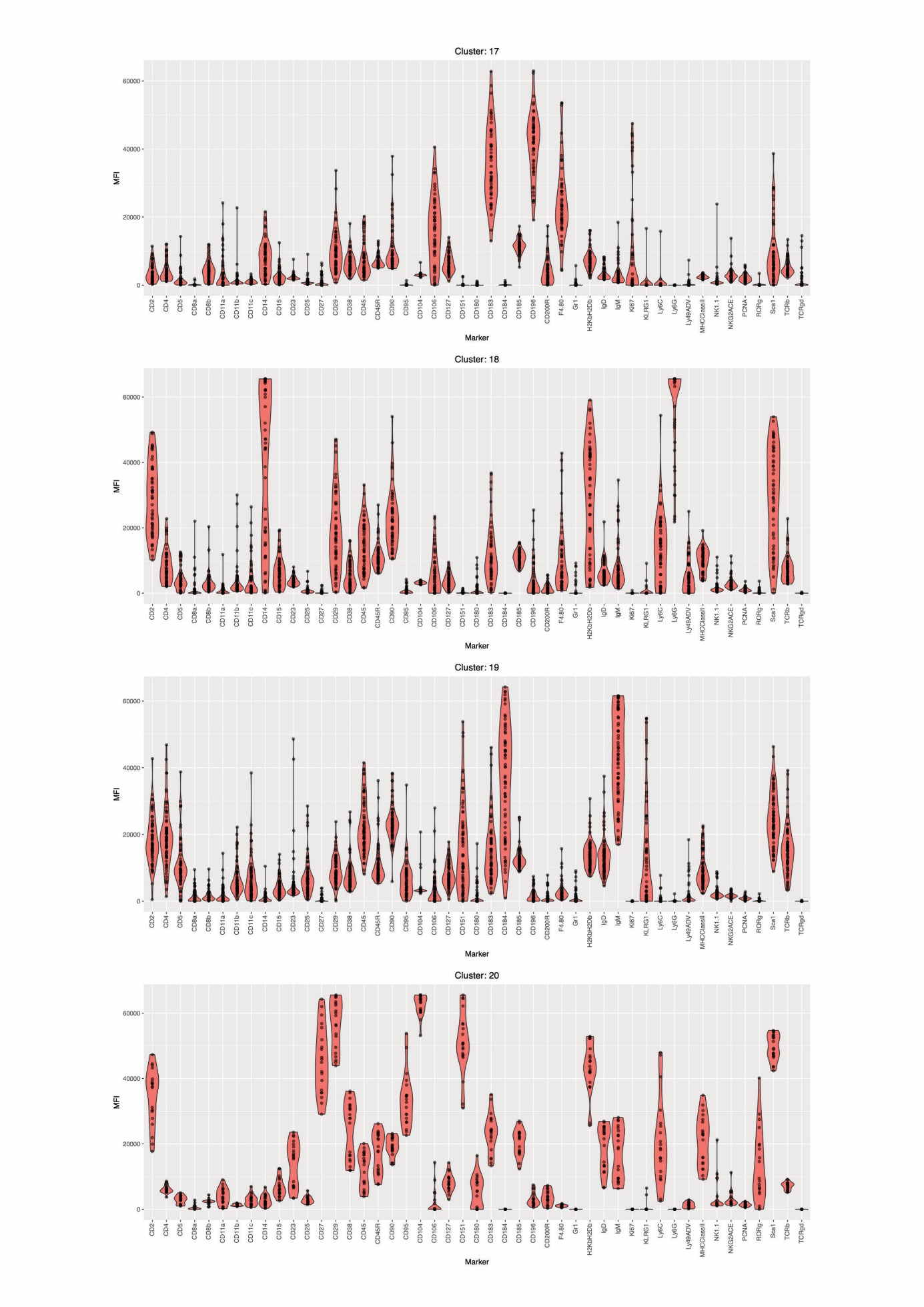


**Supplementary Fig. S8. Marker distributions for the hierarchical clusters identified in the murine spleen section in Fig. 3c.**

Individual violin plots for each cluster identified in Fig. 3c.


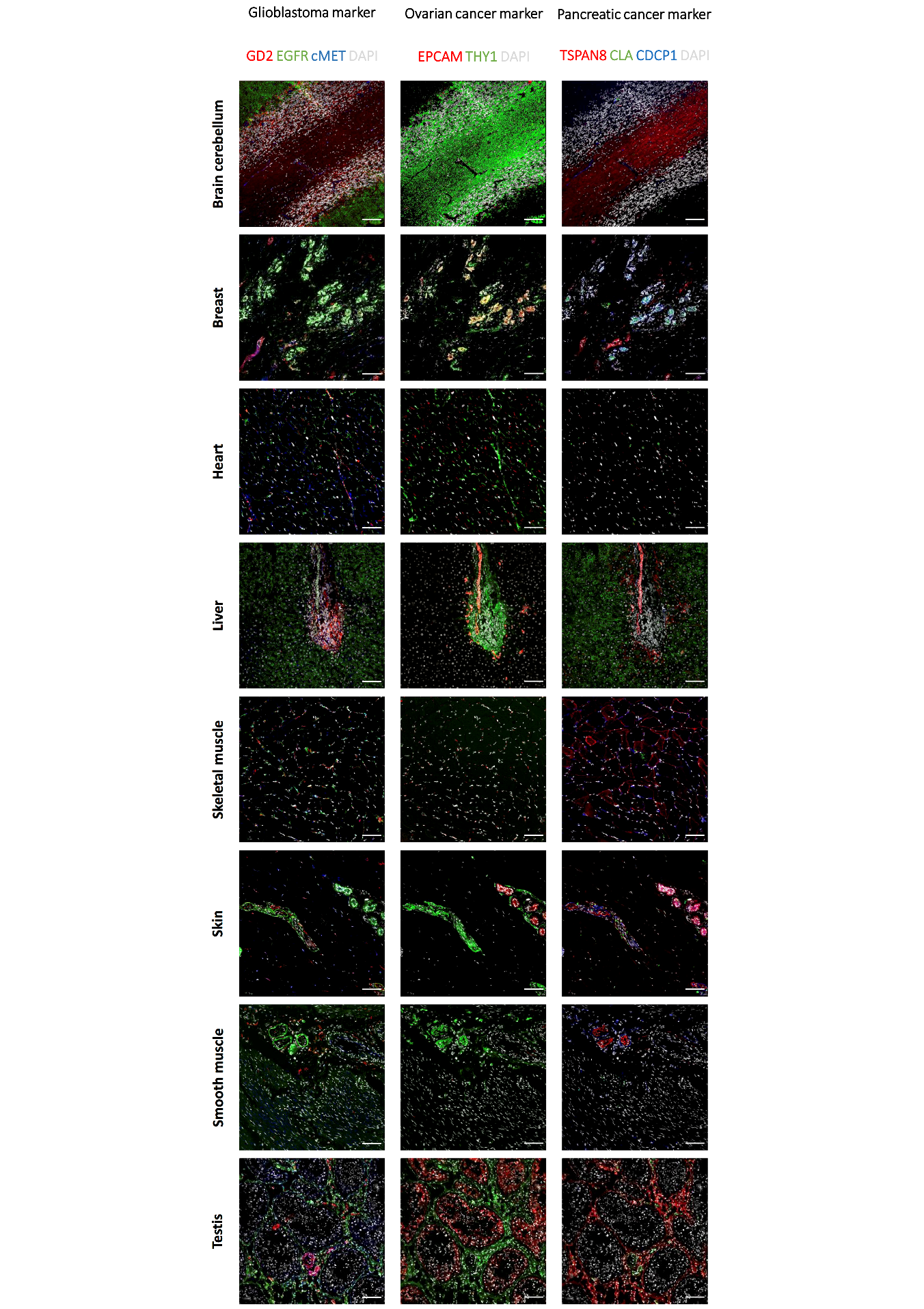


**Supplementary Fig. S9. Ultrahigh-content imaging validates expression of target candidates on healthy human tissues to predict safety and toxicity of target candidates.**

Fresh-frozen human tissues were sliced and fixed with acetone. The subsequent screening was performed on the MACSima Imaging Platform by sequential staining with antibodies. Healthy human tissues, i.e. brain cerebellum, breast, heart, liver, skeletal muscle, skin, smooth muscle, and testis, were analyzed for the expression of glioblastoma target candidates (left panel), GD2 is shown in red, EGFR in green, cMET in blue, and DAPI in white; ovarian cancer target candidates (middle panel), EPCAM is shown in red, THY1 in green, and DAPI in white; pancreatic cancer target candidates (right panel), TSPAN8 is shown in red, CLA in green, CDCP1 in blue, and DAPI in white. Scale bar represents 100 µm.


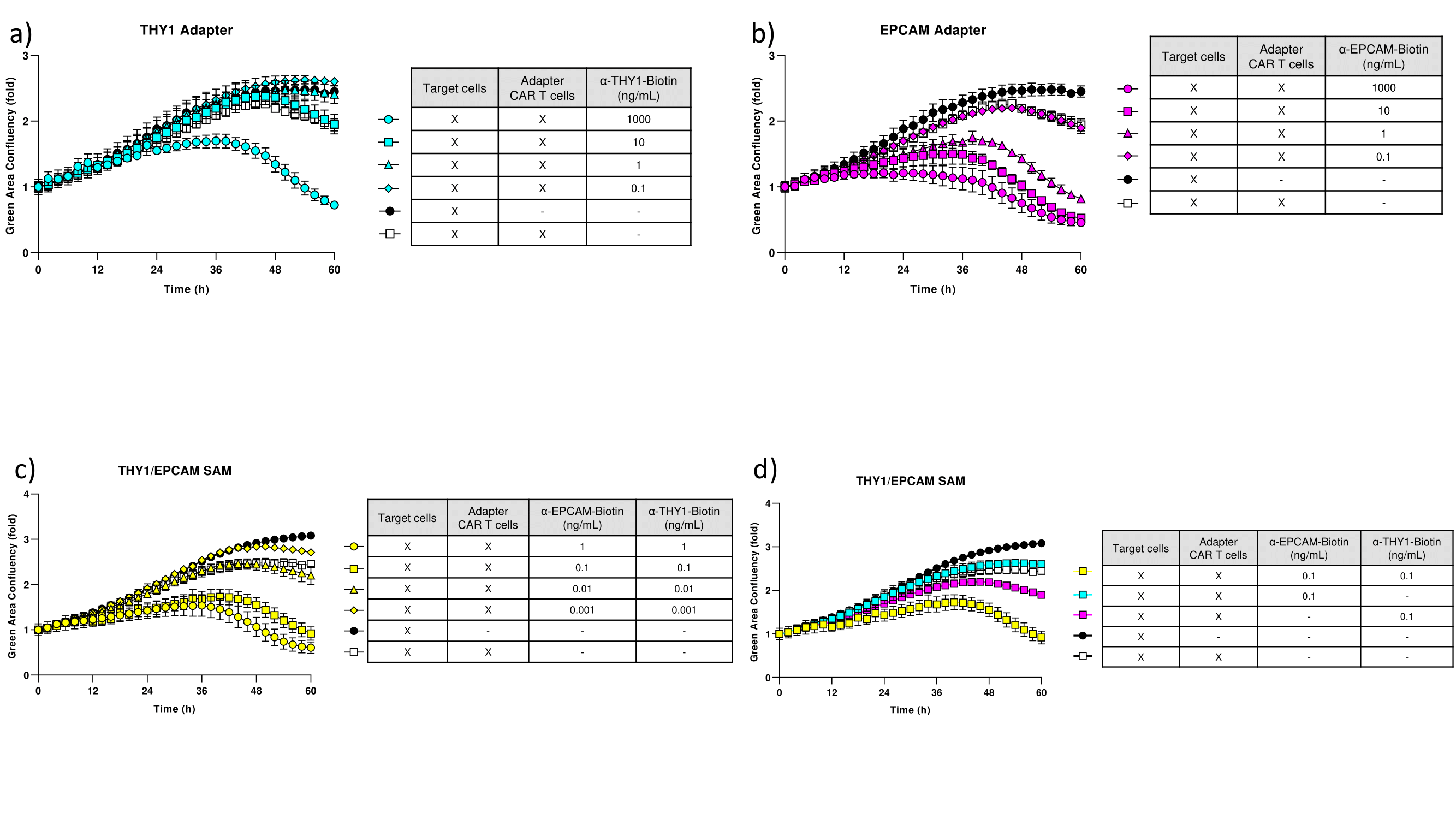


**Supplementary Fig. S10. Adapter CAR T cells targeting THY1 and EPCAM are cytolytically active against ovarian cancer cell line co-expressing THY1 and EPCAM.**

Primary human T cells were isolated and transduced with a CAR construct against biotin. Anti-biotin CAR T cells were co-cultured with THY1-, EPCAM-, and GFP-expressing target cells for 72 h in the presence of varying doses of biotinylated THY1 (a), biotinylated EPCAM (b), or biotinylated THY1 and EPCAM (c) antibodies, respectively. Comparison of suboptimal single adapter doses with the combinatorial use of suboptimal adapter doses (d) as illustrated in Figure 7. GFP-fluorescence was measured over time. CAR T cell-mediated lysis of target cells results in decreased GFP-fluorescence. Each data point represents the mean of a technical replicate +/- SEM. Representative graph from three experiments shown.

**Table S1: List of anti-human antibodies.**

| **Figure** | **Gene Name** | **CD nomenclature** | **Clone** | **Conjugate** | **Order Number** | **Company** | **Gene Identifier** |
| --- | --- | --- | --- | --- | --- | --- | --- |
| 1c/S1d | CD4; CD4MUT | CD4 | REAL103 | PE | 130-117-725 | Miltenyi Biotec | 920 |
| 1c/S1d | B4; CD19; CVID3 | CD19 | REAL106 | FITC | 130-112-073 | Miltenyi Biotec | 930 |
| 1c/S1d | CD28; TP44 | CD28 | REAL105 | APC | 130-112-096 | Miltenyi Biotec | 940 |
| 1c/S1d | CD3E; IMD18; T3E; TCRE | CD3 | REAL104 | PE | 130-116-560 | Miltenyi Biotec | 916 |
| 1c/S1d | B220; CD45; CD45R; GP180; L-CA; LCA; LY5; PTPRC; T200 | CD45RA | REAL164 | FITC | 130-112-079 | Miltenyi Biotec | 5788 |
| 1c/S1d | CD11C; ITGAX; SLEB6 | CD11c | REAL351 | APC | 130-117-721 | Miltenyi Biotec | 3687 |
| S1c | CD8; CD8A; CD8B; CD8B1; LEU2; LY3; LYT3; MAL; P32; P37 | CD8 | REAL100 | FITC | 130-112-070 | Miltenyi Biotec | 925 |
| 2a | CD4; CD4MUT | CD4 | REA623 | APC | 130-113-222 | Miltenyi Biotec | 920 |
| 2a | CD4; CD4MUT | CD4 | REA623 | Biotin | 130-113-224 | Miltenyi Biotec | 920 |
| 2b/2c/2d | CD3E; IMD18; T3E; TCRE | CD3 | REA613 | APC | 130-113-135 | Miltenyi Biotec | 916 |
| 2b/2c/2d | CD3E; IMD18; T3E; TCRE | CD3 | REA613 | biotin | 130-113-137 | Miltenyi Biotec | 916 |
| 2b/2c/2d | B220; CD45; CD45R; GP180; L-CA; LCA; LY5; PTPRC; T200 | CD45 | REA747 | VioBlue | 130-110-637 | Miltenyi Biotec | 5788 |
| S3 | B4; CD19; CVID3 | CD19 | LT19 | APC | 130-113-727 | Miltenyi Biotec | 930 |
| S3 | CD56; MSK39; NCAM; NCAM1 | CD56 | REA196 | APC | 130-113-310 | Miltenyi Biotec | 4684 |
| 4a | AI836096; ALXDRD; GFAP | X | REA335 | PE | 130-118-489 | Miltenyi Biotec | 2670 |
| 4a | D630048A14RIK; KI-67; KI67; KIA; MIB-; MIB-1; MKI67; PPP1R105 | X | REA183 | FITC | 130-117-803 | Miltenyi Biotec | 4288 |
| 4a | O4 | X | REA576 | PE | 130-117-823 | Miltenyi Biotec |  |
| 4a | AI504299; B430115D02RIK; EA6; EAAT1; GLAST; GLAST-1; GLAST1; GLU-T; GLUT-1; GMT1; MGLUT1; SLC1A3 | X | ACSA-1 | PE | 130-118-483 | Miltenyi Biotec | 6507 |
| 4a / 5 | DIAR5; EGP-2; EGP314; EGP40; EPCAM; ESA; HNPCC8; KS1/4; KSA; M4S1; MIC18; MK-1; TACSTD1; TROP1 | CD326 | HEA-125 | PE | 130-113-826 | Miltenyi Biotec | 4072 |
| 4a / 5 | CD31; CD31/ENDOCAM; ENDOCAM; GPIIA'; PECA1; PECAM-1; PECAM1 | CD31 | AC128 | PE | 130-119-142 | Miltenyi Biotec | 5175 |
| 4a / 5 | CD34 | CD34 | AC136 | PE | 130-113-741 | Miltenyi Biotec | 947 |
| 4a / 5 | END; ENG; HHT1; ORW1 | CD105 | 43A4E1 | PE | 130-117-808 | Miltenyi Biotec | 2022 |
| 4a / 5 | VIM | X | REA409 | PE | 130-123-997 | Miltenyi Biotec | 7431 |
| 4a | CD11B; CD11B/CD18; CR3; CR3A; F730045J24RIK; ITGAM; LY-40; MAC-1; MAC-1A; MAC1; MAC1A; MO1A; SLEB6 | CD11b | REA592 | PE | 130-113-806 | Miltenyi Biotec | 3684 |
| 4a / 5 | CD45 | CD45 | 5B1 | PE | 130-113-680 | Miltenyi Biotec | 5788 |
| 4a | CD68 | CD68 | Y1/82A | PE | 130-118-486 | Miltenyi Biotec | 968 |
| 4a | PDGFRα | CD140a | Alpha-R1 | PE | #556002 | BD Pharmingen | 5156 |
| 4a | BBL; BCC7; BFY; BHY; LFS1; P44; P53; TP53; TRP53 | X | REA609 | PE | 130-123-646 | Miltenyi Biotec | 7157 |
| 4a | Synaptophysin, SYP, MRX96, MRXsynaptophysin | X | EP10 | PE | ABIN2484428 | Antibodies online | 6855 |
| 4a | Nestin, NES, Nbla00170 | X | 25/Nestin | PE | #561230 | BD Pharmingen | 10763 |
| 4a | CD44; CDW44; CSPG8; ECMR-III; HCELL; HUTCH-I; IN; LHR; MC56; MDU2; MDU3; MIC4; PGP1 | CD44 | REA690 | PE | 130-113-904 | Miltenyi Biotec | 960 |
| 4a | CD49f (integrinα6) | CD49f | REA518 | PE | 130-119-807 | Miltenyi Biotec | 3655 |
| 4a / 5 | CD29 | CD29 | TS2/16 | PE | 130-101-275 | Miltenyi Biotec | 3688 |
| 4a / 5 | AGGRUS; GP36; GP38; GP40; HT1A-1; OTS8; PA2.26; PDPN; T1A; T1A-2; T1A2; TI1A | X | REA446 | PE | 130-117-799 | Miltenyi Biotec | 10630 |
| 4a / 5 | EGFR; ERBB; ERBB1; HER1; MENA; NISBD2; PIG61 | X | REA688 | PE | 130-110-586 | Miltenyi Biotec | 1956 |
| 4a | NKX2-2, NKX2.2, NKX2B, NK2 homeobox 2 | X | NX2/294 | PE | NBP2-33058PE | Novus Biologicals | 4821 |
| 4a / 5 | MET, MET proto-oncogene, receptor tyrosine kinase, AUTS9, HGFR, RCCP2, c-Met, DFNB97, OSFD | X | 1G7NB | PE | NBP2-44306PE | Novus Biologicals | 4233 |
| 4a | Anti-GABRA1, ECA4, EIEE19, EJM, EJM5, gamma-aminobutyric acid type A receptor alpha1 subunit, gamma-aminobutyric acid type A receptor subunit alpha1 | X | S95-35 | PE | orb182082 | biorbyt | 2554 |
| 4a | SLC12A5, KCC2, Chloride potassium symporter 5, EIEE34, EIG14, hKCC2, solute carrier family 12 member 5 | X | S1-12 | PE | ABIN2484122 | Antibodies-online | 57468 |
| 4a | Anti-S100A4, 18A2, 42A, CAPL, FSP1, MTS1, P9KA, PEL98, S100 calcium binding protein A4 | X | 1C4 | PE | NBP2-36431PE | Novus Biologicals | 6275 |
| 4a | 10Q23DEL; 2310035O07RIK; A130070J02RIK; AI463227; B430203M17RIK; BZS; CWS1; DEC; GLM2; MHAM; MMAC; MMAC1; MMAC11; PTEN; PTENBETA; TEP1 | X | REA270 | PE | 130-103-715 | Miltenyi Biotec | 5728 |
| 4a / 5 | GD2 | X | 14.2GA | PE | #562100 | BD Pharmingen |  |
| 4a | 4F2; 4F2HC; 4F2LC; 4T2HC; CD98; CD98HC; D16S469E; E16; LAT1; MDU1; MPE16; NACAE; SLC3A2; SLC7A5 | CD98 | REA387 | PE | 130-120-137 | Miltenyi Biotec | 6520; 8140 |
| 4a | LRP1, A2MR, APOER, APR, CD91, IGFBP3R, LRP, LRP1A, TGFBR5, low density lipoprotein receptor-related protein 1, LDL receptor related protein 1, KPA, IGFBP3R1, IGFBP-3R | CD91 | A2MR-a2 | PE | ABIN119414 | Antibodies-online | 4035 |
| 4a | FZD3, Fz-3 | X | 169310 | PE | FAB1001P | Novus Biologicals | 7976 |
| 4a / 5 | CD90; CDW90; THY1 | CD90 | DG3 | PE | 130-117-537 | Miltenyi Biotec | 7070 |
| 4a | CD301A; CLEC10A; M-ASGP-BP-1; MGL; MGL1 | CD81 | REA513 | PE | 130-118-481 | Miltenyi Biotec | 975 |
| 4a | 16.3A5; 1F5; CD59; EJ16; EJ30; EL32; G344; HRF-20; HRF20; MAC-IP; MACIF; MEM43; MIC11; MIN1; MIN2; MIN3; MIRL; MSK21; P18-20 | CD59 | REA496 | PE | 130-120-048 | Miltenyi Biotec | 966 |
| 4a / 5 | CD47; IAP; MER6; OA3 | CD47 | REA220 | PE | 130-123-980 | Miltenyi Biotec | 961 |
| 4a | CD151; GP27; MER2; PETA-3; RAPH; SFA1; TSPAN24 | CD151 | REA265 | PE | 130-103-727 | Miltenyi Biotec | 977 |
| 4a / 5 | CD146; MCAM; MUC18 | CD146 | 541-10B2 | PE | 130-123-950 | Miltenyi Biotec | 4162 |
| 4a / 5 | CD271; GP80-LNGFR; LNGFR; NGFR; P75; P75(NTR); P75NGFR; P75NTR; RNNGFRR; TNFRSF16 | CD271 | REA648 | PE | 130-118-851 | Miltenyi Biotec | 4804 |
| 4a | AC133; CD133; CORD12; MCDR2; MSTP061; PROM1; PROML1; RP41; STGD4 | CD133/2 | 293C3 | PE | 130-113-748 | Miltenyi Biotec | 8842 |
| 4a | CD159A; KLRC1; NKG2; NKG2A | CD159a | REA110 | PE | 130-114-092 | Miltenyi Biotec | 3821 |
| 4a | CD213A2; CT19; IL-13R; IL13BP; IL13RA2 | CD213a2 | REA308 | PE | 130-104-553 | Miltenyi Biotec | 3598 |
| 4a | CCRL1; CMKBRL1; CMKDR1; CX3CR1; GPR13; GPRV28; V28 | X | REA385 | PE | 130-122-956 | Miltenyi Biotec | 1524 |
| 4a | CA9; CAIX; MN | X | REA658 | PE | 130-123-340 | Miltenyi Biotec | 768 |
| 4a | CD72; CD72B; LYB2 | CD72 | REA231 | PE | 130-101-336 | Miltenyi Biotec | 971 |
| 4a | CAML1; CD171; HSAS; HSAS1; L1CAM; MASA; MIC5; N-CAM-L1; N-CAML1; NCAM-L1; S10; SPG1 | CD171 | REA163 | PE | 130-100-692 | Miltenyi Biotec | 3897 |
| 4a | ADGRE2; CD312; EMR2; VBU | CD312 | REA302 | PE | 130-119-810 | Miltenyi Biotec | 30817 |
| 4a | CD263; DCR1; DCR1-TNFR; LIT; TNFRSF10C; TRAIL-R3; TRAILR3; TRID | CD263 | DJR3 | PE | 130-124-904 | Miltenyi Biotec | 8794 |
| 4a | CD240CE; CD240D; DIIIC; RH; RH30; RH30A; RH4; RHC; RHCE; RHCE(152N); RHCEDVA(TT); RHD; RHDCW; RHDEL; RHE; RHII; RHIVB(J); RHIXB; RHK562-II; RHPI; RHPII; RHVI; RHVIII; RHXIII | CD240DCE | REA327 | PE | 130-126-544 | Miltenyi Biotec | 6007; 6006 |
| 4a | CD56; MSK39; NCAM; NCAM1 | CD56 | REA196 | PE | 130-113-874 | Miltenyi Biotec | 4684 |
| 4a | CD30; D1S166E; KI-1; TNFRSF8 | CD30 | Ki-2 | PE | 130-120-783 | Miltenyi Biotec | 943 |
| 4a / 5 | CD276; 4Ig-B7-H3; B7-H3; B7H3; B7RP-2 | CD276 | FM276 | PE | 130-120-781 | Miltenyi Biotec | 80381 |
| 4a | C3ORF13; IL-1RACP; IL1R3; IL1RAP | X | REA558 | PE | 130-108-756 | Miltenyi Biotec | 3556 |
| 4a | CCR1; CD191; CKR-1; CKR1; CMKBR1; HM145; MIP1AR; SCYAR1 | CD191 | REA158 | PE | 130-100-368 | Miltenyi Biotec | 1230 |
| 4a | CD103; HUMINAE; ITGAE | CD103 | Ber-ACT 8 | PE | 130-103-709 | Miltenyi Biotec | 3682 |
| 4a | CD85K; ILT-3; ILT3; LILRB4; LIR-5; LIR5 | CD85k | REA141 | PE | 130-099-809 | Miltenyi Biotec | 11006 |
| 4a | CD66B; CD67; CEACAM8; CGM6; NCA-95 | CD66b | REA306 | PE | 130-122-966 | Miltenyi Biotec | 1088 |
| 4a | CD42A; GP9; GPIX | CD42a | REA209 | PE | 130-123-979 | Miltenyi Biotec | 2815 |
| 4a | ALPS1B; APT1LG1; APTL; CD178; CD95-L; CD95L; FASL; FASLG; TNFSF6; TNLG1A | CD178 | NOK-1 | PE | 130-118-491 | Miltenyi Biotec | 356 |
| 4a | BDPLT1; BDPLT3; BSS; CD42B; CD42B-ALPHA; DBPLT3; GP1B; GP1BA; GPIBA; GPIBALPHA; VWDP | CD42b | REA185 | PE | 130-123-959 | Miltenyi Biotec | 2811 |
| 4a | 4-1BB; CD137; CDW137; ILA; TNFRSF9 | CD137 | 4B4-1 | PE | 130-119-970 | Miltenyi Biotec | 3604 |
| 4a | BR; CD49B; GPIA; HPA-5; ITGA2; VLA-2; VLAA2 | CD49b | REA188 | PE | 130-123-976 | Miltenyi Biotec | 3673 |
| 4a | ABC20; ABCB1; CD243; CLCS; GP170; MDR1; P-GP; PGY1 | CD243 | REA495 | PE | 130-124-449 | Miltenyi Biotec | 5243 |
| 4a | CCRL2, ACKR5, CKRX, CRAM, CRAM-A, CRAM-B, HCR, C-C motif chemokine receptor like 2 | X | 152254 | PE | FAB23501P | R&D Systems | 9034 |
| 4a / 5 | CALJA; CD73; E5NT; EN; ENT; NT; NT5; NT5E; NTE | CD73 | AD2 | PE | 130-120-152 | Miltenyi Biotec | 4907 |
| 4a | BDPLT16; BDPLT2; CD61; GP3A; GPIIIA; GT; ITGB3 | CD61 | Y2/51 | PE | 130-124-903 | Miltenyi Biotec | 3690 |
| 4a | CCR10; GPR2 | X | REA326 | PE | 130-120-547 | Miltenyi Biotec | 2826 |
| 4a | BTCC-1; CD9; DRAP-27; MIC3; MRP-1; TSPAN-29; TSPAN29 | CD9 | SN4 C3-3A2 | PE | 130-123-986 | Miltenyi Biotec | 928 |
| 4a | CD51; ITGAV; MSK8; VNRA; VTNR | CD51 | REA181 | PE | 130-100-606 | Miltenyi Biotec | 3685 |
| 4a | CD170; CD33L2; OB-BP2; OBBP2; SIGLEC-5; SIGLEC5 | CD170 | 1A5 | PE | 130-101-785 | Miltenyi Biotec | 8778 |
| 4a | B7-2; B7.2; B70; CD28LG2; CD86; LAB72 | CD86 | FM95 | PE | 130-114-098 | Miltenyi Biotec | 942 |
| 4a | 5F7; BSG; CD147; EMMPRIN; EMPRIN; OK; TCSF | X | REA476 | PE | 130-126-551 | Miltenyi Biotec | 682 |
| 4a | BK65A6.2; SUSD2 | X | W5C5 | PE | 130-117-794 | Miltenyi Biotec | 56241 |
| 4a | CD354; TREM-1; TREM1 | X | REA213 | PE | 130-128-124 | Miltenyi Biotec | 54210 |
| 4a | ATPDASE; CD39; ENTPD1; NTPDASE-1; SPG64 | CD39 | MZ18-23C8 | PE | 130-118-668 | Miltenyi Biotec | 953 |
| 4a | CD85J; ILT-2; ILT2; LILRB1; LIR-1; LIR1; MIR-7; MIR7; PIR-B; PIRB | CD85j | GHI/75 | PE | 130-125-109 | Miltenyi Biotec | 10859 |
| 4a | CD27-L; CD27L; CD27LG; CD70; TNFSF7; TNLG8A | CD70 | REA292 | PE | 130-122-973 | Miltenyi Biotec | 970 |
| 4a / 5 | CCK-4; CCK4; PTK7 | X | 188B | PE | 130-122-967 | Miltenyi Biotec | 5754 |
| 4a | BDPLT16; BDPLT2; CD41; CD41B; GP2B; GPIIB; GT; GTA; HPA3; ITGA2B; PPP1R93 | CD41b | REA336 | PE | 130-105-071 | Miltenyi Biotec | 3674 |
| 4a | 7B4; CD144; CDH5 | X | REA199 | PE | 130-118-495 | Miltenyi Biotec | 1003 |
| 4a | CD96; TACTILE | X | REA195 | PE | 130-123-977 | Miltenyi Biotec | 10225 |
| 4a | CD294; CRTH2; DL1R; DP2; GPR44; PTGDR2 | CD294 | BM16 | PE | 130-114-126 | Miltenyi Biotec | 11251 |
| 4a | CD49C; FRP-2; GAP-B3; GAPB3; ILNEB; ITGA3; MSK18; VCA-2; VL3A; VLA3A | CD49c | REA360 | PE | 130-118-672 | Miltenyi Biotec | 3675 |
| 4a | CD180; LY64; LY78; RP105 | CD180 | MHR73-11 | PE | 130-098-488 | Miltenyi Biotec | 4064 |
| 4a | BLAME; CD353; SBBI42; SLAMF8 | X | REA394 | PE | 130-106-733 | Miltenyi Biotec | 56833 |
| 4a | BMPR1B, ALK-6, ALK6, CDw293, AMDD, BDA1D, BDA2, bone morphogenetic protein receptor type 1B | X | 477914 | PE | FAB5051P | R&D Systems | 658 |
| 4a | ALTPRP; ASCR; CD230; CJD; GSS; KURU; P27-30; PRIP; PRNP; PRP; PRP27-30; PRP33-35C; PRPC | X | REA203 | PE | 130-126-442 | Miltenyi Biotec | 5621 |
| 4a | CD307B; FCRH2; FCRL2; IFGP4; IRTA4; SPAP1; SPAP1A; SPAP1B; SPAP1C | X | REA474 | PE | 130-123-336 | Miltenyi Biotec | 79368 |
| 4a | CD49A; ITGA1; VLA1 | CD49a | TS2/7 | PE | 130-101-399 | Miltenyi Biotec | 3672 |
| 4a | ACKR1; CCBP1; CD234; DARC; DARC/ACKR1; DFY; FY; GPD; GPFY; WBCQ1 | X | REA376 | PE | 130-125-868 | Miltenyi Biotec | 2532 |
| 4a | IL-21; IL-22; IL-D110; IL-TIF; IL22; ILTIF; TIFA; TIFIL-23; ZCYTO18 | X | REA466 | PE | 130-120-140 | Miltenyi Biotec | 50616 |
| 4a | BLR1; CD185; CXCR5; MDR15 | X | REA103 | FITC | 130-098-418 | Miltenyi Biotec | 643 |
| 4a / 5 | CARD2; CK-18; CK-8; CK19; CK7; CK8; CYK18; CYK8; K18; K19; K1CS; K2C7; K2C8; K7; K8; KO; KRT18; KRT19; KRT7; KRT8; SCL | X | CK3-6H5 | FITC | 130-119-141 | Miltenyi Biotec | 3855; 3856; 3875; 3880 |
| 4a | EGFRvIII | X | pc | FITC | orb187796 | biorbyt | 1956 |
| 4a | EPHA2, Epha2, AW545284, Eck, Myk2, Sek-2, Sek2, ARCC2, CTPA, CTPP1, CTRCT6, EPH receptor A2, ECK |  |  |  | FAB3035G | R&D Systems | 1969 |
| 4a | CD125; CDW125; HSIL5R3; IL5R; IL5RA | X | REA705 | PE | 130-110-602 | Miltenyi Biotec | 3568 |
| 4a | CD179A; IGI; IGVPB; VPREB; VPREB1 | X | HSL96 | PE | 130-120-136 | Miltenyi Biotec | 7441 |
| 4a | EGFR; ERBB; ERBB1; HER1; MENA; NISBD2; PIG61 | X | REA696 | PE | 130-110-599 | Miltenyi Biotec | 1956 |
| 4a | 9030024J15RIK; AI552599; EGFR; ERBB; ERBB-1; ERBB1; ERRB1; ERRP; HER1; MENA; NISBD2; PIG61; WA-2; WA2; WA5 | X | REA439 | PE | 130-107-716 | Miltenyi Biotec | 1956 |
| 4a | EGFR; ERBB; ERBB1; HER1; MENA; NISBD2; PIG61 | X | REA644 | PE | 130-110-014 | Miltenyi Biotec | 1956 |
| 4a | CD56; E-NCAM; MSK39; N-CAM; N-CAM-1; NCAM; NCAM-1; NCAM-C; NCAM1; NCAMC | X | 2-2B | PE | 130-117-394 | Miltenyi Biotec | 4684 |
| 4a | DELTA; DELTA1; DL1; DLL1 | X | MHD1-314 | PE | 130-096-966 | Miltenyi Biotec | 28514 |
| 4a | 9930111A19RIK; AOS5; AOVD1; HN1; LIN-12; MIS6; N1; NOTCH1; TAN1 | X | REA357 | PE | 130-105-925 | Miltenyi Biotec | 4851 |
| 4a | CSPG4; HMW-MAA; MCSP; MCSPG; MEL-CSPG; MSK16; NG2 | X | EP-1 | PE | 130-099-413 | Miltenyi Biotec | 1464 |
| 4a / 5 | ADGRG1; BFPP; BPPR; GPR56; TM7LN4; TM7XN1 | X | REA467 | PE | 130-125-250 | Miltenyi Biotec | 9289 |
| 4a | BIT; CD172A; MFR; MYD-1; P84; PTPNS1; SHPS1; SIRP; SIRPA | X | REA144 | PE | 130-123-971 | Miltenyi Biotec | 140885 |
| 4a | CLSS; CMS17; LRP-4; LRP10; LRP4; MEGF7; SOST2 | X | REA552 | PE | 130-126-563 | Miltenyi Biotec | 4038 |
| 4a | ALK; CD246; NBLST3 | X | REA425 | PE | 130-106-493 | Miltenyi Biotec | 238 |
| 4a | GP200; PC; PCLP; PCLP-1; PODXL | X | REA157 | PE | 130-122-965 | Miltenyi Biotec | 5420 |
| 4a | GP200; PC; PCLP; PCLP-1; PODXL | X | REA246 | PE | 130-123-334 | Miltenyi Biotec | 5420 |
| 4a | ABCD-3; C3XKINE; CX3CL1; CXC3; CXC3C; FRACTALKINE; NEUROTACTIN; NTN; NTT; SCYD1 | X | REA281 | PE | 130-108-114 | Miltenyi Biotec | 6376 |
| 4a | SAF2; SIGLEC-8L; SIGLEC8 | X | 7C9 | PE | 130-098-727 | Miltenyi Biotec | 27181 |
| 4a | CD286; TLR6 | X | REA382 | PE | 130-106-631 | Miltenyi Biotec | 10333 |
| 4a | CD85H; ILT1; LILRA2; LIR-7; LIR7 | X | REA219 | PE | 130-100-891 | Miltenyi Biotec | 11027 |
| 4a | CD282; LY105; TIL4; TLR2 | X | REA109 | PE | 130-127-922 | Miltenyi Biotec | 7097 |
| 4a | CD200; MOX1; MOX2; MRC; OX-2 | CD200 | OX-104 | PE | 130-124-277 | Miltenyi Biotec | 4345 |
| 4a / 5 | ALCAM; CD166; MEMD | CD166 | REA442 | PE | 130-118-487 | Miltenyi Biotec | 214 |
| 4a | CD18; ITGB2; LAD; LCAMB; LFA-1; MAC-1; MF17; MFI7 | CD18 | TS1/18 | PE | 130-117-791 | Miltenyi Biotec | 3689 |
| 4a | CD3E; IMD18; T3E; TCRE | CD3 | REA613 | PE | 130-113-701 | Miltenyi Biotec | 916 |
| 4a | CD4; CD4MUT | CD4 | VIT4 | PE | 130-113-776 | Miltenyi Biotec | 920 |
| 4a | CD8; CD8A; CD8B; CD8B1; LEU2; LY3; LYT3; MAL; P32; P37 | CD8 | BW135/80 | PE | 130-113-720 | Miltenyi Biotec | 925 |
| 4a | CD25; IDDM10; IL2R; IL2RA; IMD41; P55; TCGFR | CD25 | 4E3 | PE | 130-113-844 | Miltenyi Biotec | 3559 |
| 4a | CD279; HPD-1; HPD-L; HSLE1; PD-1; PD1; PDCD1; SLEB2 | X | PD1.3.1.3 | PE | 130-117-533 | Miltenyi Biotec | 5133 |
| 4a | CD14 | CD14 | TÜK4 | PE | 130-113-709 | Miltenyi Biotec | 929 |
| 4a | CD11C; ITGAX; SLEB6 | CD11c | REA618 | PE | 130-114-113 | Miltenyi Biotec | 3687 |
| 4a | B4; CD19; CVID3 | CD19 | LT19 | PE | 130-113-731 | Miltenyi Biotec | 930 |
| 4a | CD340; ERBB2; HER-2; HER-2/NEU; HER2; MLN 19; NEU; NGL; TKR1 | X | 24D2 | PE | 130-106-753 | Miltenyi Biotec | 2064 |
| 4a | CD274, B7-H, B7H1, PD-L1, PDCD1L1, PDCD1LG1, PDL1, CD274 molecule, Programmed cell death ligand 1, hPD-L1 | CD274 | 29E.2A3 | PE | 329706 | BioLegend | 29126 |
| 4a / 5 | AGM6; B29; CD79B; IGB | CD79b | REA120 | PE | 130-123-967 | Miltenyi Biotec | 974 |
| 4c | CD3E; IMD18; T3E; TCRE | CD3 | BW264/56 | PE | 130-113-125 | Miltenyi Biotec | 916 |
| 4c | CD4; CD4MUT | CD4 | vit4 | PE | 130-113-214 | Miltenyi Biotec | 920 |
| 4c | CD8; CD8A; CD8B; CD8B1; LEU2; LY3; LYT3; MAL; P32; P37 | CD8 | BW135/80 | PE | 130-113-158 | Miltenyi Biotec | 925 |
| 4c | CD11C; ITGAX; SLEB6 | CD11c | MJ4-27G12 | PE | 130-113-580 | Miltenyi Biotec | 3687 |
| 4c | CD14 | CD14 | TÜK4 | PE | 130-113-147 | Miltenyi Biotec | 929 |
| 4c | CD18; ITGB2; LAD; LCAMB; LFA-1; MAC-1; MF17; MFI7 | CD18 | TS1/18 | PE | 130-117-679 | Miltenyi Biotec | 3689 |
| 4c | B4; CD19; CVID3 | CD19 | LT19 | PE | 130-113-169 | Miltenyi Biotec | 930 |
| 4c | B1; BP35; CD20; CVID5; LEU-16; MS4A1; MS4A2; S7 | CD20 | REA780 | PE | 130-111-338 | Miltenyi Biotec | 931 |
| 4c | BLAST-2; CD23; CD23A; CLEC4J; FCE2; FCER2; IGEBF | CD23 | M-L23.4 | PE | 130-123-716 | Miltenyi Biotec | 2208 |
| 4c | CD24; CD24A | CD24 | 32D12 | PE | 130-123-719 | Miltenyi Biotec | 100133941 |
| 4c | CD27; S152; S152. LPFS2; T14; TNFRSF7; TP55 | CD27 | REA499 | PE | 130-113-640 | Miltenyi Biotec | 939 |
| 4c | CD28; TP44 | CD28 | 15e8 | PE | 130-126-172 | Miltenyi Biotec | 940 |
| 4c | CD29; FNRB; GPIIA; ITGB1; MDF2; MSK12; VLA-BETA; VLAB | CD29 | TS2/16 | PE | 130-101-273 | Miltenyi Biotec | 3688 |
| 4c | CD31; CD31/ENDOCAM; ENDOCAM; GPIIA'; PECA1; PECAM-1; PECAM1 | CD31 | AC128 | PE | 130-118-965 | Miltenyi Biotec | 5175 |
| 4c | CD34 | CD34 | AC136 | PE | 130-113-179 | Miltenyi Biotec | 947 |
| 4c | ADPRC 1; ADPRC1; CD38 | CD38 | IB6 | PE | 130-113-427 | Miltenyi Biotec | 952 |
| 4c | CD44; CDW44; CSPG8; ECMR-III; HCELL; HUTCH-I; IN; LHR; MC56; MDU2; MDU3; MIC4; PGP1 | CD44 | DB105 | PE | 130-113-335 | Miltenyi Biotec | 960 |
| 4c | B220; CD45; CD45R; GP180; L-CA; LCA; LY5; PTPRC; T200 | CD45RO | UCHL1 | PE | 130-113-550 | Miltenyi Biotec | 5788 |
| 4c | B220; CD45; CD45R; GP180; L-CA; LCA; LY5; PTPRC; T200 | CD45RA | T6D11 | PE | 130-113-356 | Miltenyi Biotec | 5788 |
| 4c | B220; CD45; CD45R; GP180; L-CA; LCA; LY5; PTPRC; T200 | CD45 | 5B1 | PE | 130-113-118 | Miltenyi Biotec | 5788 |
| 4c | AHUS2; CD46; MCP; MIC10; TLX; TRA2.10 | CD46 | REA312 | PE | 130-104-508 | Miltenyi Biotec | 4179 |
| 4c | CD47; IAP; MER6; OA3 | CD47 | REA220 | PE | 130-123-754 | Miltenyi Biotec | 961 |
| 4c | BR; CD49B; GPIA; HPA-5; ITGA2; VLA-2; VLAA2 | CD49B | REA188 | PE | 130-123-976 | Miltenyi Biotec | 3673 |
| 4c | CD49C; FRP-2; GAP-B3; GAPB3; ILNEB; ITGA3; MSK18; VCA-2; VL3A; VLA3A | CD49c | REA360 | PE | 130-118-672 | Miltenyi Biotec | 3675 |
| 4c | CD49E; FNRA; ITGA5; VLA-5; VLA5A | CD49e | NKI-SAM1 | PE | 130-097-225 | Miltenyi Biotec | 3678 |
| 4c | 5033401O05RIK; AI115430; CD49F; CD49FB; ITGA6; VLA-6 | CD49f | GoH3 | PE | 130-097-250 | Miltenyi Biotec | 3655 |
| 4c | CD51; ITGAV; MSK8; VNRA; VTNR | CD51 | REA181 | PE | 130-100-556 | Miltenyi Biotec | 3685 |
| 4c | BB2; CD54; ICAM1; P3.58 | CD54 | REA266 | PE | 130-120-711 | Miltenyi Biotec | 3383 |
| 4c | CD55; CHAPLE; CR; CROM; DAF; TC | CD55 | JS11 | PE | 130-101-764 | Miltenyi Biotec | 1604 |
| 4c | CD56; MSK39; NCAM; NCAM1 | CD56 | REA196 | PE | 130-113-312 | Miltenyi Biotec | 4684 |
| 4c | AG3; CD58; LFA-3; LFA3 | CD58 | TS2/9 | PE | 130-101-193 | Miltenyi Biotec | 965 |
| 4c | 16.3A5; 1F5; CD59; EJ16; EJ30; EL32; G344; HRF-20; HRF20; MAC-IP; MACIF; MEM43; MIC11; MIN1; MIN2; MIN3; MIRL; MSK21; P18-20 | CD59 | REA496 | PE | 130-120-048 | Miltenyi Biotec | 966 |
| 4c | BGP; BGP1; BGPI; CD66C; CD66D; CD66E; CEA; CEACAM1; CEACAM3; CEACAM5; CEACAM6; CEAL; CGM1; NCA; W264; W282 | CD66acde | REA428 | PE | 130-106-344 | Miltenyi Biotec | 634; 4680; 1084; 1048 |
| 4c | CD66C; CEACAM6; CEAL; NCA | CD66c | REA414 | PE | 130-123-270 | Miltenyi Biotec | 4680 |
| 4c | CD68; GP110; LAMP4; SCARD1 | CD68 | Y1/82A | PE | 130-118-486 | Miltenyi Biotec | 968 |
| 4c | CD71; IMD46; P90; T9; TFR; TFR1; TFRC; TR; TRFR | CD71 | AC102 | PE | 130-099-219 | Miltenyi Biotec | 7037 |
| 4c | CALJA; CD73; E5NT; EN; ENT; NT; NT5; NT5E; NTE | CD73 | AD2 | PE | 130-120-066 | Miltenyi Biotec | 4907 |
| 4c | B7; B7-1; B7.1; BB1; CD28LG; CD28LG1; CD80; LAB7 | CD80 | 2D10 | PE | 130-117-683 | Miltenyi Biotec | 941 |
| 4c | B7-2; B7.2; B70; CD28LG2; CD86; LAB72 | CD86 | FM95 | PE | 130-113-572 | Miltenyi Biotec | 942 |
| 4c | CD90; CDW90; THY1 | CD90 | DG3 | PE | 130-117-388 | Miltenyi Biotec | 7070 |
| 4c | CD94; KLRD1 | CD94 | REA113 | PE | 130-098-974 | Miltenyi Biotec | 3824 |
| 4c | ALPS1A; APO-1; APT1; CD951; FAS; FASTM; TNFRSF6 | CD95 | DX2 | PE | 130-123-706 | Miltenyi Biotec | 355 |
| 4c | CD104; GP150; ITGB4 | CD104 | REA236 | PE | 130-123-756 | Miltenyi Biotec | 3691 |
| 4c | END; ENG; HHT1; ORW1 | CD105 | 43A4E1 | PE | 130-117-808 | Miltenyi Biotec | 2022 |
| 4c | CD107A; LAMP1; LAMPA; LGP120 | CD107a | H4A3 | PE | 130-119-872 | Miltenyi Biotec | 3916 |
| 4c | C-KIT; CD117; KIT; PBT; SCFR | CD117 | A3C6E2 | PE | 130-113-544 | Miltenyi Biotec | 3815 |
| 4c | AC133; CD133; CORD12; MCDR2; MSTP061; PROM1; PROML1; RP41; STGD4 | CD133 | AC133 | PE | 130-113-108 | Miltenyi Biotec | 8842 |
| 4c | ACT35; CD134; IMD16; OX40; TNFRSF4; TXGP1L | CD134 | ACT35 | PE | 130-116-655 | Miltenyi Biotec | 7293 |
| 4c | CD138; SDC; SDC1; SYND1; SYNDECAN | CD138 | 44F9 | PE | 130-119-840 | Miltenyi Biotec | 6382 |
| 4c | AHUS6; BDCA3; CD141; THBD; THPH12; THRM; TM | CD141 | AD5-14H12 | PE | 130-113-318 | Miltenyi Biotec | 7056 |
| 4c | CD142; F3; TF; TFA | CD142 | HTF-1 | PE | 130-098-742 | Miltenyi Biotec | 2152 |
| 4c | CD146; MCAM; MUC18 | CD146 | 541-10B2 | PE | 130-123-711 | Miltenyi Biotec | 4162 |
| 4c | 5F7; BSG; CD147; EMMPRIN; EMPRIN; OK; TCSF | CD147 | REA282 | PE | 130-123-764 | Miltenyi Biotec | 682 |
| 4c | ALPS5; CD; CD152; CELIAC3; CTLA-4; CTLA4; GRD4; GSE; IDDM12 | CD152 | BNI3 | PE | 369603 | BioLegend | 1493 |
| 4c | CD155; HVED; NECL-5; NECL5; PVR; PVS; TAGE4 | CD155 | PV404.19 | PE | 130-105-846 | Miltenyi Biotec | 5817 |
| 4c | AD10; AD18; ADAM10; CD156C; CDW156; HST18717; KUZ; MADM; RAK | CD156c | REA309 | PE | 130-104-407 | Miltenyi Biotec | 102 |
| 4c | SELPLG, CD162, CLA, PSGL-1, PSGL1 | CD162 | REA319 | PE | 130-104-707 | Miltenyi Biotec | 6404 |
| 4c | CD163; M130; MM130; SCARI1 | CD163 | GHI/61.1 | PE | 130-123-249 | Miltenyi Biotec | 9332 |
| 4c | ALCAM; CD166; MEMD | CD166 | REA442 | PE | 130-118-349 | Miltenyi Biotec | 214 |
| 4c | CAML1; CD171; HSAS; HSAS1; L1CAM; MASA; MIC5; N-CAM-L1; N-CAML1; NCAM-L1; S10; SPG1 | CD171 | REA163 | PE | 130-100-692 | Miltenyi Biotec | 3897 |
| 4c | ALPS1B; APT1LG1; APTL; CD178; CD95-L; CD95L; FASL; FASLG; TNFSF6; TNLG1A | CD178 | NOK-1 | PE | 130-118-353 | Miltenyi Biotec | 356 |
| 4c | CD182; CD183; CKR-L2; CMKAR3; CXCR3; GPR9; IP10-R; MIG-R; MIGR | CD183 | REA232 | PE | 130-120-452 | Miltenyi Biotec | 2833 |
| 4c | CD184; CXCR4; D2S201E; FB22; HM89; HSY3RR; LAP-3; LAP3; LCR1; LESTR; NPY3R; NPYR; NPYRL; NPYY3R; WHIM; WHIMS | CD184 | 12G5 | PE | 130-117-690 | Miltenyi Biotec | 7852 |
| 4c | CC-CKR-5; CCCKR5; CCR-5; CCR5; CD195; CKR-5; CKR5; CMKBR5; IDDM22 | CD195 | REA245 | PE | 130-117-356 | Miltenyi Biotec | 1234 |
| 4c | MSR1; SRA; SR-A; CD204; SR-AI; phSR1; phSR2; SCARA1; SR-AII; SR-AIII | CD204 | REA460 | PE | 130-123-266 | Miltenyi Biotec | 4481 |
| 4c | BA541I19.1; CD206; CLEC13D; CLEC13DL; HMR; MMRL1; MRC1 | CD206 | DCN228 | PE | 130-124-233 | Miltenyi Biotec | 4360 |
| 4c | CD223; LAG3 | CD223 | REA351 | PE | 130-120-470 | Miltenyi Biotec | 3902 |
| 4c | ADMCKD; ADMCKD1; CA 15-3; CD227; EMA; H23AG; KL-6; MAM6; MCD; MCKD; MCKD1; MUC-1; MUC-1/SEC; MUC-1/X/ZD; MUC1; PEM; PEMT; PUM | CD227 | REA448 | PE | 130-120-142 | Miltenyi Biotec | 4582 |
| 4c | AU; BCAM; CD239; LU; MSK19 | CD239 | REA276 | PE | 130-103-842 | Miltenyi Biotec | 4059 |
| 4c | CD240CE; CD240D; DIIIC; RH; RH30; RH30A; RH4; RHC; RHCE; RHCE(152N); RHCEDVA(TT); RHD; RHDCW; RHDEL; RHE; RHII; RHIVB(J); RHIXB; RHK562-II; RHPI; RHPII; RHVI; RHVIII; RHXIII | CD240DCE | REA327 | PE | 130-126-544 | Miltenyi Biotec | 6007; 6006 |
| 4c | PDCD1LG2, B7DC, Btdc, CD273, PD-L2, PDCD1L2, PDL2, bA574F11.2, programmed cell death 1 ligand 2 | CD273 | MIH18 | PE | 345505 | BioLegend | 80380 |
| 4c | CD274, B7-H, B7H1, PD-L1, PDCD1L1, PDCD1LG1, PDL1, CD274 molecule, Programmed cell death ligand 1, hPD-L1 | CD274 | 29E.2A3 | PE | 329705 | BioLegend | 29126 |
| 4c | CD276; 4Ig-B7-H3; B7-H3; B7H3; B7RP-2 | CD276 | FM276 | PE | 130-120-712 | Miltenyi Biotec | 80381 |
| 4c | AILIM; CCLP; CD278; CRP-1; CVID1; H4; ICOS; LY115 | CD278 | REA192 | PE | 130-120-155 | Miltenyi Biotec | 29851 |
| 4c | CD279; HPD-1; HPD-L; HSLE1; PD-1; PD1; PDCD1; SLEB2 | CD279 | PD1.3.1.3 | PE | 130-117-384 | Miltenyi Biotec | 5133 |
| 4c | ATP1B3; ATPB-3; CD298 | CD298 | REA217 | PE | 130-126-421 | Miltenyi Biotec | 483 |
| 4c | CD309; FLK1; KDR; VEGFR; VEGFR2 | CD309 | ES8-20E6 | PE | 130-120-480 | Miltenyi Biotec | 3791 |
| 4c | BST2; CD317; TETHERIN | CD317 | REA202 | PE | 130-101-707 | Miltenyi Biotec | 684 |
| 4c | CD318; CDCP1; SIMA135; TRASK | CD318 | REA194 | PE | 130-101-215 | Miltenyi Biotec | 64866 |
| 4c | DIAR5; EGP-2; EGP314; EGP40; EPCAM; ESA; HNPCC8; KS1/4; KSA; M4S1; MIC18; MK-1; TACSTD1; TROP1 | CD326 | HEA-125 | PE | 130-113-264 | Miltenyi Biotec | 4072 |
| 4c | AITR; CD357; GITR; GITR-D; TNFRSF18 | CD357 | DT5D3 | PE | 130-121-331 | Miltenyi Biotec | 8784 |
| 4c | NF-A3; OCT-3; OCT-3/4; OCT-4; OCT3; OCT3/4; OCT4; OTF-3; OTF-4; OTF3; OTF3-RS7; OTF3G; OTF4; POU5F1 | X | REA338 | PE | 130-123-771 | Miltenyi Biotec | 5460 |
| 4c | CO-029; TM4SF3; TSPAN8 | X | REA443 | PE | 130-117-540 | Miltenyi Biotec | 7103 |
| 4c | SSEA1 | X | REA321 | PE | 130-117-689 | Miltenyi Biotec |  |
| 4c | SSEA4 | X | REA101 | PE | 130-122-914 | Miltenyi Biotec |  |
| 4c | DR4; DRB1; DRB4; HLA-DPB1; HLA-DR1B; HLA-DR3B; HLA-DR4B; HLA-DRA; HLA-DRA1; HLA-DRB; HLA-DRB1; HLA-DRB3; HLA-DRB4; HLA-DRB5; SS1 | X | AC122 | PE | 130-113-402 | Miltenyi Biotec | 3123; 3122; 3127; 3126; 3125 |
| 4c | D630048A14RIK; KI-67; KI67; KIA; MIB-; MIB-1; MKI67; PPP1R105 | X | REA183 | PE | 130-120-417 | Miltenyi Biotec | 4288 |
| 4c | VIM | X | REA409 | PE | 130-123-774 | Miltenyi Biotec | 7431 |
| 4c | AGGRUS; GP36; GP38; GP40; HT1A-1; OTS8; PA2.26; PDPN; T1A; T1A-2; T1A2; TI1A | X | REA446 | PE | 130-117-687 | Miltenyi Biotec | 10630 |
| 4c | IGHD | X | IgD26 | PE | 130-094-539 | Miltenyi Biotec | 3495 |
| 4c | IGA1; IGHA1; IGHA2 | X | IS11-8E10 | PE | 130-113-476 | Miltenyi Biotec | 3493 |
| 4c | IGG3; IGHG1; IGHG2; IGHG3; IGHG4 | X | IS11-3B2.2.3 | PE | 130-119-878 | Miltenyi Biotec | 3500; 3501; 3502; 3503 |
| 4c | HLA-A; HLAA | X | REA142 | PE | 130-099-536 | Miltenyi Biotec | 3105 |
| 4c | AS; B-4901; D6S204; HLA-A; HLA-B; HLA-C; HLA-JY3; HLAA; HLAB; HLAC; HLC-C; MHC; PSORS1 | X | REA230 | PE | 130-120-055 | Miltenyi Biotec | 3105; 3106; 3107 |
| 4c | CARD2; CK-18; CK-8; CK19; CK8; CYK18; CYK8; K18; K19; K1CS; K2C8; K8; KO; KRT18; KRT19; KRT8 | X | REA801 | FITC | 130-112-189 | Miltenyi Biotec | 3856; 3875; 3880 |
| 4c | Rat IgM – isotype control antibodies | X | ES26-13D3.4 | PE | 130-102-672 | Miltenyi Biotec |  |
| 4c | Mouse IgG1 – isotype control antibodies | X | IS5-21F5 | PE | 130-113-200 | Miltenyi Biotec |  |
| 4c | REA Control antibodies | X | REA293 | PE | 130-104-613 | Miltenyi Biotec |  |
| 4c | CELIAC1; D6S205; D6S205E; DQ-A1; DQB3; DVB; DVBETA; DX-ALPHA; HLA-DQA; HLA-DQA1; HLA-DQA2; HLA-DQB; HLA-DQB1; HLA-DQB2; HLA-DQB3; HLA-DVB; HLA-DXA; HLA-DXB; HLADQA2; IDDM1 | X | REA303 | PE | 130-123-765 | Miltenyi Biotec | 3117; 3118; 3119; 3120; 3121 |
| 4c | CLA; SELPLG; PSGL-1 | X | HECA-452 | PE | 130-091-635 | Miltenyi Biotec | 6404 |
| 4b/5/S4 | anti-Biotin | X | Bio3-18E7 | PE | 130-113-853 | Miltenyi Biotec |  |
| 4b/5/S4 | anti-HA | X | GG8-1F3.3.1 | PE | 130-120-786 | Miltenyi Biotec |  |
| 4b/5/S4 | MET, MET proto-oncogene, receptor tyrosine kinase, AUTS9, HGFR, RCCP2, c-Met, DFNB97, OSFD | X | 1G7NB | PE | NBP2-44306PE | Novus Biologicals | 4233 |
| 4b/5/S4 | EGFR; ERBB; ERBB1; HER1; MENA; NISBD2; PIG61 | X | REA688 | PE | 130-110-586 | Miltenyi Biotec | 1956 |
| 4b/5/S4 | SSEA4 | X | REA101 | PE | 130-122-958 | Miltenyi Biotec |  |
| 4b/5/S4 | VIM | X | REA409 | PE | 130-123-997 | Miltenyi Biotec | 7431 |
| 4b/5/S4 | CCK-4; CCK4; PTK7; PTK7CCK5 | X | 188B | PE | 130-122-967 | Miltenyi Biotec | 5754 |
| 4b/5/S4 | TM4SF1; H-L6; L6; M3S1; TAAL6 | X | REA851 | PE | 130-112-904 | Miltenyi Biotec | 4071 |
| 4b/5/S4 | CD318; CDCP1; SIMA135; TRASK | CD318 | REA194 | PE | 130-101-251 | Miltenyi Biotec | 64866 |
| 4b/5/S4 | CALLA; CD10; CMT2T; MME; NEP; SCA43; SFE | CD10 | 97C5 | PE | 130-123-951 | Miltenyi Biotec | 4311 |
| 4b/5/S4 | CLA; SELPLG; PSGL-1 | X | HECA-452 | PE | 130-123-946 | Miltenyi Biotec | 6404 |
| 4b/5/S4 | AGGRUS; GP36; GP38; GP40; HT1A-1; OTS8; PA2.26; PDPN; T1A; T1A-2; T1A2; TI1A | X | REA446 | PE | 130-117-687 | Miltenyi Biotec | 10630 |
| 4b/5/S4 | FOLR1, FBP, FOLR, Folate receptor 1, folate receptor 1 (adult), folate receptor alpha, FRalpha | X | LK26 | PE | 908304 | BioLegend | 2348 |
| 4b/5/S4 | CO-029; TM4SF3; TSPAN8 | X | REA443 | PE | 130-117-540 | Miltenyi Biotec | 7103 |
| 4b/5/S4 | CD146; MCAM; MUC18 | CD146 | 541-10B2 | PE | 130-123-711 | Miltenyi Biotec | 4162 |
| 4b/5/S4 | CD31; CD31/ENDOCAM; ENDOCAM; GPIIA'; PECA1; PECAM-1; PECAM1 | CD31 | AC128 | PE | 130-119-142 | Miltenyi Biotec | 5175 |
| 4b/5/S4 | END; ENG; HHT1; ORW1 | CD105 | 43A4E1 | PE | 130-117-808 | Miltenyi Biotec | 2022 |
| 4b/5/S4 | CD29; FNRB; GPIIA; ITGB1; MDF2; MSK12; VLA-BETA; VLAB | CD29 | TS2/16 | PE | 130-101-275 | Miltenyi Biotec | 3688 |
| 4b/5/S4 | CD271; GP80-LNGFR; LNGFR; NGFR; P75; P75(NTR); P75NGFR; P75NTR; RNNGFRR; TNFRSF16 | CD271 | REA648 | PE | 130-118-851 | Miltenyi Biotec | 4804 |
| 4b/5/S4 | CD276; 4Ig-B7-H3; B7-H3; B7H3; B7RP-2 | CD276 | FM276 | PE | 130-120-781 | Miltenyi Biotec | 80381 |
| 4b/5/S4 | ADGRG1; BFPP; BPPR; GPR56; TM7LN4; TM7XN1 | X | REA467 | PE | 130-125-250 | Miltenyi Biotec | 9289 |
| 4b/5/S4 | CD90; CDW90; THY1 | CD91 | DG3 | PE | 130-117-537 | Miltenyi Biotec | 7070 |
| 4b/5/S4 | CD107A; LAMP1; LAMPA; LGP120 | CD107a | H4A3 | PE | 130-119-958 | Miltenyi Biotec | 3916 |
| 4b/5/S4 | REA Control antibodies | X | REA293 | PE | 130-104-613 | Miltenyi Biotec |  |
| 4b/5/S4 | CD34 | CD34 | AC136 | PE | 130-113-741 | Miltenyi Biotec | 947 |
| 4b/5/S4 | GD2 | X | 14.2GA | PE | #562100 | BD Pharmingen |  |
| 4b/5/S4 | CD66C; CEACAM6; CEAL; NCA | CD66c | REA414 | PE | 130-123-331 | Miltenyi Biotec | 4680 |
| 4b/5/S4 | ALCAM; CD166; MEMD | CD167 | REA442 | PE | 130-118-487 | Miltenyi Biotec | 214 |
| 4b/5/S4 | CARD2; CK-18; CK-8; CK19; CK8; CYK18; CYK8; K18; K19; K1CS; K2C8; K8; KO; KRT18; KRT19; KRT8 | X | REA801 | PE | 130-112-346 | Miltenyi Biotec | 3856; 3875; 3880 |
| 4b/5/S4 | B220; CD45; CD45R; GP180; L-CA; LCA; LY5; PTPRC; T200 | CD46 | 5B1 | PE | 130-113-680 | Miltenyi Biotec | 5788 |
| 4b/5/S4 | CD235A; GPA; GPERIK; GPSAT; GYPA; HGPMIV; HGPMIXI; HGPSTA(C); MN; MNS; PAS-2 | CD235a | REA175 | PE | 130-120-613 | Miltenyi Biotec | 2993 |
| 4b/5/S4 | AGM6; B29; CD79B; IGB | CD79b | REA120 | PE | 130-123-737 | Miltenyi Biotec | 974 |
| 4b/5/S4 | CD47; IAP; MER6; OA3 | CD47 | REA220 | PE | 130-123-980 | Miltenyi Biotec | 961 |
| 4b/5/S4 | anti-c-myc | X | SH1-26E7.1.6 | FITC | 130-116-485 | Miltenyi Biotec |  |
| 4b/5/S4 | DIAR5; EGP-2; EGP314; EGP40; EPCAM; ESA; HNPCC8; KS1/4; KSA; M4S1; MIC18; MK-1; TACSTD1; TROP1 | CD327 | HEA-125 | PE | 130-113-264 | Miltenyi Biotec | 4072 |
| 4b/5/S4 | anti-biotin | X | Bio3-18E7 | PE | 130-113-853 | Miltenyi Biotec |  |
| 4b/5/S4 | anti-HA | X | GG8-1F3.3.1 | PE | 130-123-553 | Miltenyi Biotec |  |
| 4b/5/S4 | MET, MET proto-oncogene, receptor tyrosine kinase, AUTS9, HGFR, RCCP2, c-Met, DFNB97, OSFD | X | 1G7NB | PE | NBP2-44306PE | Novus Biologicals | 4233 |
| 4b/5/S4 | EGFR; ERBB; ERBB1; HER1; MENA; NISBD2; PIG61 | EGFR | REA688 | PE | 130-110-586 | Miltenyi Biotec | 1956 |
| 4b/5/S4 | CALJA; CD73; E5NT; EN; ENT; NT; NT5; NT5E; NTE | CD73 | AD2 | PE | 130-120-152 | Miltenyi Biotec | 4907 |
| S6 | PTPRC; B220; CD45; CD45R; GP180; L-CA; LCA; LY5; T200 | CD45 | REAL258 | APC | 130-119-589 | Miltenyi Biotec | 5788 |
| S6 | PTPRC; B220; CD45; CD45R; GP180; L-CA; LCA; LY5; T200 | CD45RA | REAL164 | FITC | 130-112-079 | Miltenyi Biotec | 5788 |
| S6 | KRT7; KRT8; KRT18; KRT19; CK7; K2C7; K7; SCL; CARD2; CK-8; CK8; CYK8; K2C8; K8; KO; CK-18; CYK18; K18; CK19; K19; K1CS | Cytokeratin | REAL648 | PE | 130-123-087 | Miltenyi Biotec | 3855; 3856; 3875; 3880 |
| S6 | HLA-A; HLA-B; HLA-C; HLAA; AS; B-4901; HLAB; D6S204; HLA-JY3; HLAC; HLC-C; MHC; PSORS1 | HLA-ABC | REAL286 | APC | 130-121-328 | Miltenyi Biotec | 3105; 3106; 3107 |
| S6 | HLA-DRB1; HLA-DRA; HLA-DRB5; HLA-DRB4; HLA-DRB3; Cd74; DRB1; HLA-DR1B; HLA-DRB; SS1; HLA-DRA1; DR4; DRB4; HLA-DR4B; HLA-DPB1; HLA-DR3B; CLIP; DHLAG; HLADG; Ia-GAMMA; Ii | HLA-DR | REAL127 | FITC | 130-121-323 | Miltenyi Biotec | 3123; 3122; 3127; 3126; 3125 |
| S6 | HLA-B; HLA-C; AS; B-4901; HLAB; D6S204; HLA-JY3; HLAC; HLC-C; MHC; PSORS1 | HLA-BC | REAL535 | PE | 130-123-366 | Miltenyi Biotec | 3106; 3107 |
| S6 | VIM | Vimentin | REAL1008 | APC | 130-127-018 | Miltenyi Biotec | 7431 |
| S6 | CD8A; CD8B; CD8; Leu2; MAL; p32; CD8B1; LEU2; LY3; LYT3; P37 | CD8 | REA734 | FITC | 130-110-677 | Miltenyi Biotec | 925; 926 |
| S6 | IGHM; AGM1; MU; VH | IgM | REAL689 | PE | 130-125-786 | Miltenyi Biotec | 3507 |
| S6 | CD3D; CD3E; CD3G; CD3-DELTA; IMD19; T3D; IMD18; T3E; TCRE; CD3-GAMMA; IMD17; T3G | CD3 | REA1151 | APC | 130-120-269 | Miltenyi Biotec | 915; 916; 917 |
| S6 | PTPRC; LCA; LY5; B220; CD45; L-CA; T200; CD45R; GP180 | CD45R (B220) | REA755 | FITC | 130-110-845 | Miltenyi Biotec | 5788 |
| S6 | NCAM1; CD56; MSK39; NCAM | CD56 | AF12-7H3 | PE | 130-113-307 | Miltenyi Biotec | 4684 |
| S6 | PTPRC; B220; CD45; CD45R; GP180; L-CA; LCA; LY5; T200 | CD45RO | REA611 | FITC | 130-113-558 | Miltenyi Biotec | 5788 |
| S6 | THY1; CD90; CDw90 | CD90 | DG3 | PE | 130-117-388 | Miltenyi Biotec | 7070 |
| S6 | CD4; CD4mut | CD4 | REA623 | FITC | 130-114-531 | Miltenyi Biotec | 920 |
| S6 | PTPRC; B220; CD45; CD45R; GP180; L-CA; LCA; LY5; T200 | CD45RB | REA119 | PE | 130-100-399 | Miltenyi Biotec | 5788 |
| S6 | CD8A; CD162; CLA; PSGL-1; PSGL1 | CD8a | REA1024 | FITC | 130-117-200 | Miltenyi Biotec | 925 |
| S6 | IGHD | IgD | REA740 | PE | 130-110-643 | Miltenyi Biotec | 3495 |
| S6 | HLA-DQA1; HLA-DQA2; HLA-DQB1; HLA-DQB2; HLA-DQB3; CELIAC1; DQ-A1; HLA-DQA; DX-ALPHA; HLA-DXA; HLADQA2; HLA-DQB; IDDM1; HLA-DQB1; HLA-DXB; D6S205; D6S205E; DQB3; DVB; DVbeta; HLA-DVB | HLA-DQ | REA303 | FITC | 130-124-222 | Miltenyi Biotec | 3117; 3118; 3119; 3120; 3121 |
| S6 | HLA-DRB1; HLA-DRA; HLA-DRB5; HLA-DRB4; HLA-DRB3; HLA-DPB1; HLA-DPA1; HLA-DQA1; HLA-DQA2; HLA-DQB1; HLA-DQB2; HLA-DQB3; DRB1; HLA-DR1B; HLA-DRB; SS1; HLA-DRA1; DR4; DRB4; HLA-DR4B; HLA-DPB1; HLA-DR3B; DPB1; HLA-DP; HLA-DP1B; HLA-DPB; DP(W3); DP(W4); HLA-DP1A; HLADP; HLASB; PLT1; CELIAC1; DQ-A1; HLA-DQA; DX-ALPHA; HLA-DXA; HLADQA2; HLA-DQB; IDDM1; HLA-DQB1; HLA-DXB; D6S205; D6S205E; DQB3; DVB; DVbeta; HLA-DVB | HLA-DR, DP, DQ | REA332 | PE | 130-120-715 | Miltenyi Biotec | 3123; 3122; 3127; 3126; 3125; 3115; 3113; 3117; 3118; 3119; 3120; 3121 |
| S6 | MRC1; CD206; CLEC13D; CLEC13DL; MMRL1; bA541I19.1; hMR; MRC1 | CD206 | REAL518 | APC | 130-122-168 | Miltenyi Biotec | 4360 |
| S6 | CD19; B4; CVID3 | CD19 | REAL106 | FITC | 130-112-073 | Miltenyi Biotec | 930 |
| S6 | FCER2; BLAST-2; CD23; CD23A; CLEC4J; FCE2; IGEBF | CD23 | REA1222 | PE | 130-124-105 | Miltenyi Biotec | 2208 |
| S6 | EPCAM; DIAR5; EGP-2; EGP314; EGP40; ESA; HNPCC8; KS1/4; KSA; M4S1; MIC18; MK-1; TACSTD1; TROP1 | CD326 (EpCAM) | REAL539 | APC | 130-122-276 | Miltenyi Biotec | 4072 |
| S6 | ENG; END; HHT1; ORW1 | CD105 | REAL218 | FITC | 130-115-182 | Miltenyi Biotec | 2022 |
| S6 | CD163; M130; MM130; SCARI1 | CD163 | REAL406 | PE | 130-121-316 | Miltenyi Biotec | 9332 |
| S6 | TOMM22; 1C9-2; MST065; MSTP065; TOM22 | TOM22 | REAL439 | APC | 130-122-277 | Miltenyi Biotec | 56993 |
| S6 | CD44; CDW44; CSPG8; ECMR-III; HCELL; HUTCH-I; IN; LHR; MC56; MDU2; MDU3; MIC4; Pgp1 | CD44 | REAL259 | FITC | 130-115-867 | Miltenyi Biotec | 960 |
| S6 | KRT5; KRT6A; KRT6B; CK5; DDD; DDD1; EBS2; K5; KRT5A; CK-6C; CK-6E; CK6A; CK6C; CK6D; K6A; K6C; K6D; KRT6C; KRT6D; PC3; CK-6B; CK6B; K6B; KRTL1; PC2; PC4 | Cytokeratin 5/6 | REAL538 | PE | 130-122-272 | Miltenyi Biotec | 3852;3853;3854 |
| S6 | SLC3A2; SLC7A5; 4F2; 4F2HC; 4T2HC; CD98; CD98HC; MDU1; NACAE; 4F2LC; D16S469E; E16; LAT1; MPE16 | CD98 | REAL355 | APC | 130-127-515 | Miltenyi Biotec | 6520; 8140 |
| S6 | ITGB4; CD104; GP150 | CD104 (Integrin β4) | REAL151 | FITC | 130-115-869 | Miltenyi Biotec | 3691 |
| S6 | COL4A1; BSVD; BSVD1; RATOR | Collagen IV | REAL567 | PE | 130-122-866 | Miltenyi Biotec | 1282 |
| S6 | IGHA1; IgA1 | IgA | REAL533 | APC | 130-123-373 | Miltenyi Biotec | 3493 |
| S6 | CD5; LEU1; T1 | CD5 | REAL382 | FITC | 130-118-369 | Miltenyi Biotec | 921 |
| S6 | CNN1; HEL-S-14; SMCC; Sm-Calp | Calponin | REAL635 | PE | 130-123-082 | Miltenyi Biotec | 1264 |
| S6 | LAMP1; CD107a; LAMPA; LGP120 | CD107a (LAMP-1) | REAL565 | APC | 130-124-360 | Miltenyi Biotec | 3916 |
| S6 | ITGAX; CD11C; SLEB6 | CD11c | REAL235 | FITC | 130-121-319 | Miltenyi Biotec | 3687 |
| S6 | CD38; ADPRC 1; ADPRC1 | CD38 | REAL633 | PE | 130-123-346 | Miltenyi Biotec | 952 |
| S6 | CD55; CHAPLE; CR; CROM; DAF; TC | CD55 (DAF) | REAL510 | APC | 130-124-551 | Miltenyi Biotec | 1604 |
| S6 | CD34 | CD34 | REAL487 | FITC | 130-122-172 | Miltenyi Biotec | 947 |
| S6 | TFRC; CD71; IMD46; T9; TFR; TFR1; TR; TRFR; p90 | CD71 | REAL444 | PE | 130-124-358 | Miltenyi Biotec | 7037 |
| S6 | NGFR; CD271; Gp80-LNGFR; TNFRSF16; p75(NTR); p75NTR | CD271 (LNGFR) | REAL709 | APC | 130-125-057 | Miltenyi Biotec | 4804 |
| S6 | GYPA; CD235a; GPA; GPErik; GPSAT; HGpMiV; HGpMiXI; HGpSta(C); MN; MNS; PAS-2 | CD235a (Glycophorin A) | REAL490 | FITC | 130-122-173 | Miltenyi Biotec | 2993 |
| S6 | CD40; Bp50; CDW40; TNFRSF5; p50 | CD40 | REAL659 | PE | 130-125-031 | Miltenyi Biotec | 958 |
| S6 | KRT20; K20; CD20; CK20; CK-20; KRT21 | Cytokeratin 20 | REAL753 | APC | 130-125-685 | Miltenyi Biotec | 54474 |
| S6 | MAPK9; JNK-55; JNK2; JNK2A; JNK2ALPHA; JNK2B; JNK2BETA; PRKM9; SAPK; SAPK1a; p54a; p54aSAPK | JNK2 (MAPK9) | REAL581 | FITC | 130-122-860 | Miltenyi Biotec | 5601 |
| S6 | ENTPD1; ATPDase; CD39; NTPDase-1; SPG64 | CD39 | REAL785 | PE | 130-125-078 | Miltenyi Biotec | 953 |
| S6 | HAVCR2; CD366; HAVcr-2; KIM-3; TIM3; TIMD-3; TIMD3; Tim-3 | TIM-3 | REAL818 | APC | 130-125-688 | Miltenyi Biotec | 84868 |
| S6 | CD68; GP110; LAMP4; SCARD1 | CD68 | REAL566 | FITC | 130-123-361 | Miltenyi Biotec | 968 |
| S6 | KRT19; K19; CK19; K1CS | Cytokeratin 19 | REAL822 | PE | 130-125-272 | Miltenyi Biotec | 3880 |
| S6 | WT1; AWT1; EWS-WT1; GUD; NPHS4; WAGR; WIT-2; WT33 | WT1 | REAL748 | APC | 130-125-782 | Miltenyi Biotec | 7490 |
| S6 | ACTA2; ACTSA | Actin (Smooth Muscle) | REAL650 | FITC | 130-123-363 | Miltenyi Biotec | 59 |
| S6 | PRF1; HPLH2; P1; PFP | Perforin | REAL787 | PE | 130-125-274 | Miltenyi Biotec | 5551 |
| S6 | ANPEP; APN; CD13; GP150; LAP1; P150; PEPN | CD13 | REAL312 | APC | 130-116-551 | Miltenyi Biotec | 290 |
| S6 | PDPN; AGGRUS; GP36; GP40; Gp38; HT1A-1; OTS8; PA2.26; T1A; T1A-2; T1A2; TI1A | Podoplanin | REAL468 | FITC | 130-125-009 | Miltenyi Biotec | 10630 |
| S6 | PROM1; AC133; CD133; CORD12; MCDR2; MSTP061; PROML1; RP41; STGD4 | CD133/1 | REAL803 | PE | 130-125-788 | Miltenyi Biotec | 8842 |
| S6 | CD53; MOX44; TSPAN25 | CD53 | REAL144 | APC | 130-112-093 | Miltenyi Biotec | 963 |
| S6 | CD99; HBA71; MIC2; MIC2X; MIC2Y; MSK5X | CD99 | REAL687 | FITC | 130-125-010 | Miltenyi Biotec | 4267 |
| S6 | CD47; IAP; MER6; OA3 | CD47 | REAL250 | PE | 130-125-964 | Miltenyi Biotec | 961 |
| S6 | ICAM1; BB2; CD54; P3.58 | CD54 (ICAM-1) | REAL146 | APC | 130-112-099 | Miltenyi Biotec | 3383 |
| S6 | CK14; EBS3; EBS4; K14; KRT14; NFJ | Cytokeratin 14 | REAL831 | FITC | 130-125-683 | Miltenyi Biotec | 3861 |
| S6 | B2M; IMD43 | β2 Microglobulin | REAL845 | PE | 130-125-963 | Miltenyi Biotec | 567 |
| S6 | CD28; Tp44 | CD28 | REAL105 | APC | 130-112-096 | Miltenyi Biotec | 940 |
| S6 | BCIE; BIE; CK1; CK10; CK5; DDD; DDD1; EBS2; EHK; EHK1; EPPK; K10; K1A; K5A; KPP; KRT1; KRT10; KRT5; NEPPK | Cytokeratin HMW | REAL645 | FITC | 130-125-783 | Miltenyi Biotec | 3848;3852;3858;3861 |
| S6 | MUC16; CA125 | CA-125 | REAL909 | PE | 130-126-212 | Miltenyi Biotec | 94025 |
| S6 | CD36; BDPLT10; CHDS7; FAT; GP3B; GP4; GPIV; PASIV; SCARB3 | CD36 | REA760 | FITC | 130-110-739 | Miltenyi Biotec | 948 |
| S6 | IL3RA; CD123; IL3RY; IL3RX; hIL-3Ra; IL3RA | CD123 | REAL270 | PE | 130-121-315 | Miltenyi Biotec | 3563 |
| S6 |  | CD15 | VIMC6 | FITC | 130-113-484 | Miltenyi Biotec |  |
| S6 | BTN3A1; BT3.1; BTF5; BTN3.1; CD277 | CD277 | BT3.1 | PE | 130-117-693 | Miltenyi Biotec | 11119 |
| S6 | ITGB2; CD18; LAD; LCAMB; LFA-1; MAC-1; MF17; MFI7 | CD18 | TS1/18 | FITC | 130-120-248 | Miltenyi Biotec | 3689 |
| S6 | DPP4; ADABP; ADCP2; CD26; DPPIV; TP103 | CD26 | FR10-11G9 | PE | 130-126-362 | Miltenyi Biotec | 1803 |
| S6 | C3AR1; AZ3B; C3AR; HNFAG09 | C3a Receptor | hC3aRZ8 | FITC | 130-108-073 | Miltenyi Biotec | 719 |
| S6 | NCAM1; CD56; MSK39; NCAM | PSA-NCAM | 2-2B | PE | 130-117-394 | Miltenyi Biotec | 4684 |
| S6 | CD14 | CD14 | REA599 | FITC | 130-110-518 | Miltenyi Biotec | 929 |
| S6 | CD164; DFNA66; MGC-24; MGC-24v; MUC-24; endolyn | CD164 | 67D2 | PE | 130-126-518 | Miltenyi Biotec | 8763 |
| S6 | KLRC1; CD159A; NKG2; NKG2A | CD159a (NKG2A) | REA110 | FITC | 130-113-565 | Miltenyi Biotec | 3821 |
| S6 | TCL1A; TCL1B; TCL1; SYN-1; TML1 | TCL1 | REA289 | PE | 130-104-095 | Miltenyi Biotec | 8115; 9623 |
| S6 | CD27; S152; S152. LPFS2; T14; TNFRSF7; Tp55 | CD27 | REA499 | FITC | 130-113-639 | Miltenyi Biotec | 939 |
| S6 | ITGA5; CD49e; FNRA; VLA-5; VLA5A | CD49e | REA686 | PE | 130-110-532 | Miltenyi Biotec | 3678 |
| S6 | MS4A1; B1; Bp35; CD20; CVID5; LEU-16; MS4A2; S7 | CD20 | REA780 | FITC | 130-111-337 | Miltenyi Biotec | 931 |
| S6 | CD1C; BDCA1; CD1; CD1A; R7 | CD1c (BDCA-1) | REA694 | PE | 130-110-536 | Miltenyi Biotec | 911 |
| S6 | F11R; CD321; JAM; JAM1; JAMA; JCAM; KAT; PAM-1 | CD321 (JAM1) | REA605 | FITC | 130-126-531 | Miltenyi Biotec | 50848 |
| S6 | ITGB3; BDPLT16; BDPLT2; CD61; GP3A; GPIIIa; GT | CD61 | REA761 | PE | 130-110-749 | Miltenyi Biotec | 3690 |
| S6 | BSG; 5F7; CD147; EMMPRIN; EMPRIN; OK; TCSF | CD147 | REA282 | FITC | 130-124-221 | Miltenyi Biotec | 682 |
| S6 | KLRB1; CD161; CLEC5B; NKR; NKR-P1; NKR-P1A; NKRP1A; hNKR-P1A | CD161 | REA631 | PE | 130-113-596 | Miltenyi Biotec | 3820 |
| S6 | RELA; NFKB3; p65 | NF-κB p65 pS529 | REA348 | FITC | 130-123-548 | Miltenyi Biotec | 5970 |
| S6 | MCAM; CD146; MUC18 | CD146 | REA773 | PE | 130-111-322 | Miltenyi Biotec | 4162 |
| S6 | CTNNB1; CTNNB; EVR7; MRD19; armadillo | β-Catenin | REA480 | FITC | 130-123-546 | Miltenyi Biotec | 1499 |
| S6 | KIT; C-Kit; CD117; PBT; SCFR | CD117 | REA787 | PE | 130-111-592 | Miltenyi Biotec | 3815 |
| S6 | CD22; SIGLEC-2; SIGLEC2 | CD22 | REA340 | FITC | 130-124-223 | Miltenyi Biotec | 933 |
| S6 | SUSD2; BK65A6.2 | SUSD2 | REA795 | PE | 130-111-641 | Miltenyi Biotec | 56241 |
| S6 | STAT5A; MGF; STAT5 | STAT5a | REA549 | FITC | 130-126-526 | Miltenyi Biotec | 6776 |
| S6 | BCAM; AU; CD239; LU; MSK19 | CD239 (BCAM) | REA276 | PE | 130-126-498 | Miltenyi Biotec | 4059 |
| S6 | FCAR; CD89; CTB-61M7.2; FcalphaRI | CD89 | REA234 | FITC | 130-101-385 | Miltenyi Biotec | 2204 |
| S6 | RB1; OSRC; PPP1R130; RB; p105-Rb; pRb; pp110 | Rb pS780 | REA433 | PE | 130-106-558 | Miltenyi Biotec | 5925 |
| S6 | HLA-B; AS; B-4901; HLAB | HLA-B12 | REA138 | PE | 130-099-863 | Miltenyi Biotec | 3106 |
| S6 | THBD; AHUS6; BDCA3; CD141; THPH12; THRM; TM | CD141 (BDCA-3) | REA674 | FITC | 130-113-661 | Miltenyi Biotec | 7056 |
| S6 | PROM1; AC133; CD133; CORD12; MCDR2; MSTP061; PROML1; RP41; STGD4 | CD133/2 | REA820 | PE | 130-112-195 | Miltenyi Biotec | 8842 |
| S6 | CD24; CD24A | CD24 | REA832 | FITC | 130-112-655 | Miltenyi Biotec | 100133941 |
| S6 | TP53; BCC7; LFS1; P53; TRP53 | p53 pS15 | REA825 | PE | 130-112-620 | Miltenyi Biotec | 7157 |
| S6 | SPN; CD43; GALGP; GPL115; LSN | CD43 | REA833 | FITC | 130-114-592 | Miltenyi Biotec | 6693 |
| S6 | PTK7; CCK-4; CCK4 | PTK7 (CCK-4) | REA836 | PE | 130-112-677 | Miltenyi Biotec | 5754 |
| S6 | FAS; ALPS1A; APO-1; APT1; CD951; FASTM; TNFRSF6; FAS | CD95 (FAS) | REA738 | FITC | 130-113-003 | Miltenyi Biotec | 355 |
| S6 | STAT4; SLEB11 | STAT4 pY693 | REA855 | PE | 130-114-492 | Miltenyi Biotec | 6775 |
| S6 | BCL2; Bcl-2; PPP1R50 | Bcl-2 | REA872 | FITC | 130-114-230 | Miltenyi Biotec | 596 |
| S6 | TACSTD2; EGP-1; EGP1; GA733-1; GA7331; GP50; M1S1; TROP2 | TROP2 | REA916 | PE | 130-115-055 | Miltenyi Biotec | 4070 |
| S6 | CEACAM1; BGP; BGP1; BGPI; CEACAM5; CD66e; CEA; CEACAM6; CD66c; CEAL; NCA | CD66ace | REA889 | FITC | 130-114-480 | Miltenyi Biotec | 634;1048;4680 |
| S6 | PMEL; D12S53E; ME20; ME20-M; ME20M; P1; P10017; SI; SIL; SILV; gp100; PMEL | Melanocyte PMEL | REA927 | PE | 130-115-473 | Miltenyi Biotec | 6490 |
| S6 | EZH2; ENX-1; ENX1b; KMT6; KMT6A; WVS; WVS2; EZH2 | EZH2 | REA907 | FITC | 130-115-218 | Miltenyi Biotec | 2146 |
| S6 | CD101; EWI-101; IGSF2; V7 | CD101 | REA954 | PE | 130-115-830 | Miltenyi Biotec | 9398 |
| S6 | CKAP4; CLIMP-63; ERGIC-63; p63 | Plasma Cell | REA908 | FITC | 130-115-223 | Miltenyi Biotec | 10970 |
| S6 | BST1; CD157 | CD157 (BST-1) | REA465 | PE | 130-116-491 | Miltenyi Biotec | 683 |
| S6 | TRBV17; TCRBV17S1; TCRBV26S1P | TCR Vβ17 | REA915 | FITC | 130-115-247 | Miltenyi Biotec | 28570 |
| S6 | PECAM1; CD31; CD31/EndoCAM; GPIIA'; PECA1; PECAM-1; endoCAM | CD31 | REA1028 | PE | 130-117-225 | Miltenyi Biotec | 5175 |
| S6 | S100A8; 60B8AG; CAGA; CFAG; CGLA; CP-10; L1Ag; MA387; MIF; MRP8; NIF; P8 | S100A8 | REA917 | FITC | 130-115-253 | Miltenyi Biotec | 6279 |
| S6 | NANOG | Nanog | REA314 | PE | 130-117-377 | Miltenyi Biotec | 79923 |
| S6 | KRT10; BCIE; BIE; CK10; EHK; K10; KPP | Cytokeratin 10 | REA933 | FITC | 130-115-403 | Miltenyi Biotec | 3858 |
| S6 | TSPAN8; CO-029; TM4SF3 | TSPAN8 | REA443 | PE | 130-117-391 | Miltenyi Biotec | 7103 |
| S6 | KRT7; CK7; K2C7; K7; SCL | Cytokeratin 7 | REA935 | FITC | 130-115-446 | Miltenyi Biotec | 3855 |
| S6 |  | SSEA-1 | REA321 | PE | 130-117-689 | Miltenyi Biotec |  |
| S6 | SDC1; CD138; SDC; SYND1; syndecan | CD138 | REA929 | FITC | 130-115-478 | Miltenyi Biotec | 6382 |
| S6 | NTRK1; MTC; TRK; TRK1; TRKA; Trk-A; p140-TrkA | TrkA | REA430 | PE | 130-117-705 | Miltenyi Biotec | 4914 |
| S6 | SAA1; PIG4; SAA; SAA2; TP53I4 | Amyloid A | REA931 | FITC | 130-115-481 | Miltenyi Biotec | 6288 |
| S6 | ITGB1; CD29; FNRB; GPIIA; MDF2; MSK12; VLA-BETA; VLAB | CD29 | REA1060 | PE | 130-118-121 | Miltenyi Biotec | 3688 |
| S6 | FCGR1A; CD64; CD64A; FCRI; IGFR1 | CD64 | REA978 | FITC | 130-116-195 | Miltenyi Biotec | 2209 |
| S6 | GFAP; ALXDRD | GFAP | REA335 | PE | 130-118-351 | Miltenyi Biotec | 2670 |
| S6 | KIR2DS1; KIR2DS2; KIR2DS3; KIR2DS4; KIR2DS5; KIR2DL1; KIR2DL2; KIR2DL3; KIR2DL4; KIR2DL5A; KIR2DL5B; CD158H; CD158a; p50.1; 183ActI; CD158J; CD158b; KIR-2DS2; NKAT-5; NKAT5; cl-49; NKAT7; CD158I; KIR-2DS4; KIR1D; KIR412; KKA3; NKAT-8; NKAT8; CD158G; NKAT9; CD158A; KIR-K64; KIR221; NKAT; NKAT-1; NKAT1; p58.1; CD158B1; NKAT-6; NKAT6; p58.2; CD158B2; GL183; KIR-023GB; KIR-K7b; KIR-K7c; KIR2DL; KIR2DS5; KIRCL23; NKAT2; NKAT2A; NKAT2B; p58; CD158D; G9P; KIR-103AS; KIR-2DL4; KIR103; KIR103AS; CD158F; KIR2DL5; KIR2DL5.1; KIR2DL5.3; KIR2DL5.2; KIR2DLX | KIR2D | REA1042 | FITC | 130-117-477 | Miltenyi Biotec | 3806; 100132285; 3808; 3809; 3810; 3802; 3803; 3804; 3805; 57292; 553128 |
| S6 | CD74; DHLAG; HLADG; II; Ia-GAMMA | CD74 | REA1103 | PE | 130-119-026 | Miltenyi Biotec | 972 |
| S6 | MKI67; KIA; MIB-; MIB-1; PPP1R105 | Ki-67 | REA183 | FITC | 130-117-691 | Miltenyi Biotec | 4288 |
| S6 | ITGA4; CD49D; IA4 | CD49d | REA545 | PE | 130-118-548 | Miltenyi Biotec | 3676 |
| S6 | CD63; LAMP-3; ME491; MLA1; OMA81H; TSPAN30 | CD63 | REA1055 | FITC | 130-118-076 | Miltenyi Biotec | 967 |
| S6 |  | CLA | REA1101 | PE | 130-119-043 | Miltenyi Biotec |  |
| S6 | CD200; MOX1; MOX2; MRC; OX-2 | CD200 | REA1067 | FITC | 130-118-128 | Miltenyi Biotec | 4345 |
| S6 | AAT4; FAA4; MYH11; SMHC; SMMHC | Myosin Smooth Muscle | REA1107 | PE | 130-119-314 | Miltenyi Biotec | 4629 |
| S6 | MS4A1; B1; Bp35; CD20; CVID5; LEU-16; MS4A2; S7 | CD20 Cytoplasmic | REA1087 | FITC | 130-118-292 | Miltenyi Biotec | 931 |
| S6 | CALD1; CDM; HCAD; LCAD; H-CAD; L-CAD; NAG22 | Caldesmon | REA1120 | PE | 130-119-344 | Miltenyi Biotec | 800 |
| S6 |  | Hepatocyte | REA1088 | FITC | 130-118-304 | Miltenyi Biotec |  |
| S6 |  | CD65 | REA1128 | PE | 130-119-518 | Miltenyi Biotec |  |
| S6 | CD276; 4Ig-B7-H3; B7-H3; B7H3; B7RP-2 | CD276 | REA1094 | FITC | 130-118-569 | Miltenyi Biotec | 80381 |
| S6 | CR1; C3BR; C4BR; CD35; KN | CD35 | REA1133 | PE | 130-119-510 | Miltenyi Biotec | 1378 |
| S6 | MART-1; MART1; MLANA | MART-1 | REA1093 | FITC | 130-118-608 | Miltenyi Biotec | 2315 |
| S6 | TP53; BCC7; LFS1; P53; TRP53 | p53 | REA1132 | PE | 130-119-502 | Miltenyi Biotec | 7157 |
| S6 | CD6; TP120 | CD6 | REA1075 | FITC | 130-118-619 | Miltenyi Biotec | 923 |
| S6 | CD79A; IGA; MB-1 | CD79a | REA1142 | PE | 130-119-722 | Miltenyi Biotec | 973 |
| S6 | CD9; BTCC-1; DRAP-27; MIC3; MRP-1; TSPAN-29; TSPAN29 | CD9 | REA1071 | FITC | 130-118-806 | Miltenyi Biotec | 928 |
| S6 | ITGA6; CD49fB; VLA-6; ITGA6 | CD49f | REA518 | PE | 130-119-767 | Miltenyi Biotec | 3655 |
| S6 | LAMP2; CD107b; LAMP-2; LAMPB; LGP-96; LGP110 | CD107b | REA1073 | FITC | 130-118-817 | Miltenyi Biotec | 3920 |
| S6 | PDCD1; CD279; PD-1; PD1; SLEB2; hPD-1; hPD-l; hSLE1 | CD279 (PD1) | REA1165 | PE | 130-120-382 | Miltenyi Biotec | 5133 |
| S6 | KIR2DL1; CD158A; KIR-K64; KIR221; NKAT; NKAT-1; NKAT1; p58.1 | CD158a (KIR2DL1) | REA284 | FITC | 130-118-961 | Miltenyi Biotec | 3802 |
| S6 | CXCR3; CD182; CD183; CKR-L2; CMKAR3; GPR9; IP10-R; Mig-R; MigR | CD183 (CXCR3) | REA232 | PE | 130-120-452 | Miltenyi Biotec | 2833 |
| S6 | TNFRSF13C; BAFF-R; BAFFR; BROMIX; CD268; CVID4; prolixin | CD268 | REA1115 | FITC | 130-119-296 | Miltenyi Biotec | 115650 |
| S6 | CCR6; BN-1; C-C CKR-6; CC-CKR-6; CCR-6; CD196; CKR-L3; CKRL3; CMKBR6; DCR2; DRY6; GPR29; GPRCY4; STRL22 | CD196 (CCR6) | REA190 | PE | 130-120-458 | Miltenyi Biotec | 1235 |
| S6 | ITGA1; CD49a; VLA1 | CD49a | REA1106 | FITC | 130-119-305 | Miltenyi Biotec | 3672 |
| S6 | SIGLEC1; CD169; SIGLEC-1; SN; dJ1009E24.1 | CD169 (Siglec-1) | REA1176 | PE | 130-121-106 | Miltenyi Biotec | 6614 |
| S6 | ANXA1; ANX1; LPC1 | Annexin I | REA1122 | FITC | 130-119-352 | Miltenyi Biotec | 301 |
| S6 | CPHD6; MCOPS5; OTX2 | OTX2 | REA1178 | PE | 130-121-187 | Miltenyi Biotec | 5015 |
| S6 | SYN1; SYN1AB; SYNI | Synapsin-1 | REA1125 | FITC | 130-119-358 | Miltenyi Biotec | 6853 |
| S6 | CD48; BCM1; BLAST; BLAST1; MEM-102; SLAMF2; hCD48; mCD48 | CD48 | REA426 | PE | 130-121-346 | Miltenyi Biotec | 962 |
| S6 | CMT1F; CMT2E; CMTDIG; NEFL; NF-L; NF68; NFL; PPP1R110 | Neurofilament | REA1127 | FITC | 130-119-495 | Miltenyi Biotec | 4747 |
| S6 | MSR1; SRA; SR-A; CD204; SR-AI; phSR1; phSR2; SCARA1; SR-AII; SR-AIII | CD204 | REA460 | PE | 130-123-266 | Miltenyi Biotec | 4481 |
| S6 | FOX-3; FOX3; HRNBP3; NEUN; RBFOX3 | NeuN | REA1131 | FITC | 130-119-492 | Miltenyi Biotec | 146713 |
| S6 | CD52; CDW52; EDDM5 | CD52 | REA164 | PE | 130-123-743 | Miltenyi Biotec | 1043 |
| S6 | CDCD3; CMD1F; CSM1; CSM2; DES; LGMD1D; LGMD1E; LGMD2R | Desmin | REA1134 | FITC | 130-119-489 | Miltenyi Biotec | 1674 |
| S6 | ITGA2; BR; CD49B; GPIa; HPA-5; VLA-2; VLAA2 | CD49b | REA188 | PE | 130-123-749 | Miltenyi Biotec | 3673 |
| S6 | BCIE; BIE; CK10; CK13; EHK; K10; K13; KPP; KRT10; KRT13; WSN2 | Cytokeratin 10/13 | REA1138 | FITC | 130-119-542 | Miltenyi Biotec | 3858;3860 |
| S6 | STAT3; ADMIO; ADMIO1; APRF; HIES | STAT3 pS727 | REA324 | PE | 130-123-768 | Miltenyi Biotec | 6774 |
| S6 | MRX96; MRXSYP; SYP | Synaptophysin | REA1143 | FITC | 130-119-724 | Miltenyi Biotec | 6855 |
| S6 | CEACAM1; CEACAM8; CEACAM6; CEACAM5; BGP; BGP1; BGPI; CD66b; CD67; CGM6; NCA-95; CD66c; CEAL; NCA; CD66e; CEA | CD66abce | REA1230 | PE | 130-124-503 | Miltenyi Biotec | 634; 1088; 4680; 1048 |
| S6 | SLC4A1; AE1; BND3; CD233; CHC; DI; EMPB3; EPB3; FR; RTA1A; SAO; SPH4; SW; WD; WD1; WR | CD233 | REA368 | FITC | 130-119-780 | Miltenyi Biotec | 6521 |
| S6 | ERBB2; CD340; HER-2; HER-2/neu; HER2; MLN 19; NEU; NGL; TKR1 | ErbB-2 (CD340) | REA1232 | PE | 130-124-466 | Miltenyi Biotec | 2064 |
| S6 | GATA3; HDR; HDRS | GATA3 | REA174 | FITC | 130-120-061 | Miltenyi Biotec | 2625 |
| S6 | ABCB1; ABC20; CD243; CLCS; GP170; MDR1; P-GP; PGY1 | CD243 (ABCB1) | REA495 | PE | 130-124-440 | Miltenyi Biotec | 5243 |
| S6 | PLP1; GPM6C; HLD1; MMPL; PLP; PLP/DM20; PMD; SPG2 | PLP | REA1155 | FITC | 130-120-273 | Miltenyi Biotec | 5354 |
| S6 | ADGRE5; CD97; TM7LN1 | CD97 | REA1242 | PE | 130-124-969 | Miltenyi Biotec | 976 |
| S6 | CEACAM6; CD66c; CEAL; NCA | CD66c | REA414 | FITC | 130-120-240 | Miltenyi Biotec | 4680 |
| S6 | CD7; GP40; LEU-9; TP41; Tp40 | CD7 | REA1244 | PE | 130-124-931 | Miltenyi Biotec | 924 |
| S6 | MPO | MPO | REA491 | FITC | 130-120-241 | Miltenyi Biotec | 4353 |
| S6 | CEACAM1; CEACAM6; CEACAM3; CEACAM5; BGP; BGP1; BGPI; CD66c; CEAL; NCA; CD66D; CEA; CGM1; W264; W282; CD66e | CD66acde | REA428 | PE | 130-125-211 | Miltenyi Biotec | 634; 4680; 1084; 1048 |
| S6 | CEACAM5; CD66e; CEA | CD66 (CEA) | REA1158 | FITC | 130-120-344 | Miltenyi Biotec | 1048 |
| S6 | TNFRSF4; ACT35; CD134; IMD16; OX40; TXGP1L | CD134 (OX40) | REA621 | PE | 130-126-024 | Miltenyi Biotec | 7293 |
| S6 | ITGA2B; BDPLT16; BDPLT2; CD41; CD41B; GP2B; GPIIb; GT; GTA; HPA3; PPP1R93 | CD41a | REA386 | FITC | 130-120-719 | Miltenyi Biotec | 3674 |
| S6 | SYK; p72-Syk | Syk | REA111 | PE | 130-126-369 | Miltenyi Biotec | 6850 |
| S6 | ATP1B3; ATPB-3; CD298 | CD298 | REA217 | FITC | 130-123-263 | Miltenyi Biotec | 483 |
| S6 | NCR1; CD335; LY94; NK-p46; NKP46 | CD335 (NKp46) | REA808 | PE | 130-112-121 | Miltenyi Biotec | 9437 |
| S6 | HLA-B; AS; B-4901; HLAB | HLA Class I Bw6 | REA143 | FITC | 130-123-264 | Miltenyi Biotec | 3106 |
| S6 | NT5E; CALJA; CD73; E5NT; NT; NT5; NTE; eN; eNT | CD73 | REA804 | PE | 130-111-908 | Miltenyi Biotec | 4907 |
| S6 | TRAV7; TRA; TCRAV7S1; IMD7; TCRA; TCRD@; TRAC; TRA | TCR Vα7.2 | REA179 | FITC | 130-123-685 | Miltenyi Biotec | 28686; 6955 |
| S6 |  | CD57 | REA769 | PE | 130-111-810 | Miltenyi Biotec |  |
| S6 | LRP4; CLSS; CMS17; LRP-4; LRP10; MEGF7; SOST2 | LRP-4 | REA552 | APC | 130-109-137 | Miltenyi Biotec | 4038 |
| S6 | GP9; CD42a; GPIX | CD42a | REA209 | FITC | 130-123-689 | Miltenyi Biotec | 2815 |
| S6 | PLXNB2; MM1; Nbla00445; PLEXB2; dJ402G11.3 | Plexin-B2 | REA626 | PE | 130-126-532 | Miltenyi Biotec | 23654 |
| S6 | BST2; CD317; TETHERIN | CD317 (BST2) | REA202 | FITC | 130-126-375 | Miltenyi Biotec | 684 |
| S6 | PRNP; ASCR; AltPrP; CD230; CJD; GSS; KURU; PRIP; PrP; PrP27-30; PrP33-35C; PrPc; p27-30 | CD230 (PrP) | REA203 | PE | 130-126-374 | Miltenyi Biotec | 5621 |
| S6 | CD82; 4F9; C33; GR15; IA4; KAI1; R2; SAR2; ST6; TSPAN27 | CD82 | REA221 | FITC | 130-126-376 | Miltenyi Biotec | 3732 |
| S6 | INSR; CD220; HHF5 | CD220 | REA260 | PE | 130-126-493 | Miltenyi Biotec | 3643 |
| S6 | CD177; HNA-2a; HNA2A; NB1; NB1 GP; PRV-1; PRV1 | CD177 | REA258 | FITC | 130-126-380 | Miltenyi Biotec | 57126 |
| S6 | STAT1; CANDF7; IMD31A; IMD31B; IMD31C; ISGF-3; STAT91 | STAT1 N-terminus | REA272 | PE | 130-126-494 | Miltenyi Biotec | 6772 |
| S6 | BSG; 5F7; CD147; EMMPRIN; EMPRIN; OK; TCSF | TRA-1-85 (CD147) | REA476 | APC | 130-107-103 | Miltenyi Biotec | 682 |
| S6 | SELPLG, CD162, CLA, PSGL-1, PSGL1 | CD162 | REA319 | FITC | 130-126-504 | Miltenyi Biotec | 6404 |
| S6 | CCL18; AMAC-1; AMAC1; CKb7; DC-CK1; DCCK1; MIP-4; PARC; SCYA18 | CCL18 (MIP-4) | REA487 | PE | 130-126-516 | Miltenyi Biotec | 6362 |
| S6 | ITGA2B; BDPLT16; BDPLT2; CD41; CD41B; GP2B; GPIIb; GT; GTA; HPA3; PPP1R93 | CD41b | REA336 | FITC | 130-126-507 | Miltenyi Biotec | 3674 |
| S6 | PLXND1; PLEXD1 | Plexin-D1 | REA542 | PE | 130-126-520 | Miltenyi Biotec | 23129 |
| S6 | FCGR3B; CD16; CD16A; CD16b; FCG3; FCGR3; FCGR3A; FCR-10; FCRIII; FCRIIIb | CD16b | REA589 | FITC | 130-126-529 | Miltenyi Biotec | 2215 |
| S6 | TRBV13; TRB; TCRBV13S1; TCRBV23S1A2T; TCRB@; TRB | TCR Vβ13.6 | REA554 | PE | 130-126-523 | Miltenyi Biotec | 28574; 6957 |
| S6 | CX3CR1; CCRL1; CMKBRL1; CMKDR1; GPR13; GPRV28; V28 | CX3CR1 | REA385 | APC | 130-119-579 | Miltenyi Biotec | 1524 |
| S6 | ITGAL; CD11A; LFA-1; LFA1A | CD11a | REA378 | FITC | 130-124-886 | Miltenyi Biotec | 3683 |
| S6 | TRBV1; TRB; TCRBV1S1P; TCRBV27S1P; TCRB@; TRB | TCR Vβ1 | REA662 | PE | 130-126-534 | Miltenyi Biotec | 28621; 6957 |
| S6 | B-ALPHA-1; K-ALPHA-1; LIS3; TUBA1A; TUBA1B; TUBA3 | α Tubulin | REA1136 | FITC | 130-119-539 | Miltenyi Biotec | 7846;10376 |
| S6 | SLC1A3; EA6; EAAT1; GLAST; GLAST1 | GLAST (ACSA-1) | ACSA-1 | PE | 130-118-344 | Miltenyi Biotec | 6507 |
| S6 | MME; CALLA; CD10; CMT2T; NEP; SCA43; SFE | CD10 | REA877 | FITC | 130-114-501 | Miltenyi Biotec | 4311 |
| S6 | PAX6; AN; AN2; ASGD5; D11S812E; FVH1; MGDA; WAGR | PAX-6 | REA507 | PE | 130-123-250 | Miltenyi Biotec | 5080 |
| S6 | SEMA4D; C9orf164; CD100; COLL4; M-sema-G; SEMAJ; coll-4 | CD100 | REA316 | FITC | 130-126-503 | Miltenyi Biotec | 10507 |
| S6 | SOX2; ANOP3; MCOPS3 | Sox2 | REA320 | PE | 130-121-053 | Miltenyi Biotec | 6657 |
| S6 | VCAM1; CD106; INCAM-100 | CD106 (VCAM-1) | REA269 | FITC | 130-124-697 | Miltenyi Biotec | 7412 |
| S6 | CCR10; GPR2 | CCR10 | REA326 | PE | 130-120-407 | Miltenyi Biotec | 2826 |
| S6 | F3; CD142; TF; TFA | CD142 | REA949 | FITC | 130-115-683 | Miltenyi Biotec | 2152 |
| S6 | IL5RA; CD125; CDw125; HSIL5R3; IL5R | CD125 (IL-5Rα) | REA705 | PE | 130-110-544 | Miltenyi Biotec | 3568 |
| S6 | CDH5; 7B4; CD144 | CD144 (VE-Cadherin) | REA199 | FITC | 130-123-688 | Miltenyi Biotec | 1003 |
| S6 | TNFRSF9; 4-1BB; CD137; CDw137; ILA | CD137 | REA765 | PE | 130-110-763 | Miltenyi Biotec | 3604 |
| S6 | PVR; CD155; HVED; NECL5; Necl-5; PVS; TAGE4 | CD155 | REA1081 | FITC | 130-118-997 | Miltenyi Biotec | 5817 |
| S6 | ACE; ACE1; CD143; DCP; DCP1 | CD143 (ACE) | REA522 | PE | 130-107-951 | Miltenyi Biotec | 1636 |
| S6 | ADAM10; AD10; AD18; CD156c; CDw156; HsT18717; MADM; RAK; kuz | CD156c (ADAM10) | REA309 | FITC | 130-104-406 | Miltenyi Biotec | 102 |
| S6 | CD151; GP27; MER2; PETA-3; RAPH; SFA1; TSPAN24 | CD151 | REA265 | PE | 130-103-662 | Miltenyi Biotec | 977 |
| S6 | SIGLEC5; CD170; CD33L2; OB-BP2; OBBP2; SIGLEC-5 | CD170 (Siglec-5) | REA1077 | FITC | 130-119-017 | Miltenyi Biotec | 8778 |
| S6 | FCGR3A; FCGR3B; CD16; CD16A; FCG3; FCGR3; FCGRIII; FCR-10; FCRIII; FCRIIIA; IGFR3; IMD20; CD16b; FCGR3A; FCRIIIb | CD16 | REA423 | PE | 130-113-393 | Miltenyi Biotec | 2214; 2215 |
| S6 | CD2; LFA-2; SRBC; T11 | CD2 | REA972 | FITC | 130-116-148 | Miltenyi Biotec | 914 |
| S6 | ALCAM; CD166; MEMD | CD166 | REA442 | PE | 130-118-349 | Miltenyi Biotec | 214 |
| S6 | PROCR; CCCA; CCD41; EPCR | CD201 (EPCR) | REA337 | FITC | 130-105-255 | Miltenyi Biotec | 10544 |
| S6 | L1CAM; CAML1; CD171; HSAS; HSAS1; MASA; MIC5; N-CAM-L1; N-CAML1; NCAM-L1; S10; SPG1 | CD171 (L1CAM) | REA163 | PE | 130-100-691 | Miltenyi Biotec | 3897 |
| S6 | CD207; CLEC4K | CD207 (Langerin) | REA770 | FITC | 130-112-210 | Miltenyi Biotec | 50489 |
| S6 | SIRPG; CD172g; SIRP-B2; SIRPB2; SIRPgamma; bA77C3.1 | CD172g (SIRPγ) | REA479 | PE | 130-107-097 | Miltenyi Biotec | 55423 |
| S6 | CD209; CDSIGN; CLEC4L; DC-SIGN; DC-SIGN1 | CD209 (DC-SIGN) | REA617 | FITC | 130-119-784 | Miltenyi Biotec | 30835 |
| S6 | CD180; LY64; Ly78; RP105 | CD180 (RP105) | REA956 | PE | 130-115-848 | Miltenyi Biotec | 4064 |
| S6 | CR2; C3DR; CD21; CR; CVID7; SLEB9 | CD21 | HB5 | FITC | 130-101-754 | Miltenyi Biotec | 1380 |
| S6 | CD1D; CD1A; R3; R3G1 | CD1d | 51.1 | PE | 130-099-982 | Miltenyi Biotec | 912 |
| S6 | IGF2R; CD222; CI-M6PR; CIMPR; M6P-R; M6P/IGF2R; MPR 300; MPR1; MPR300; MPRI | CD222 | REA187 | FITC | 130-100-865 | Miltenyi Biotec | 3482 |
| S6 | LAG3; CD223 | CD223 | REA351 | PE | 130-120-470 | Miltenyi Biotec | 3902 |
| S6 | CD244; 2B4; NAIL; NKR2B4; Nmrk; SLAMF4 | CD244 (2B4) | REA112 | FITC | 130-099-039 | Miltenyi Biotec | 51744 |
| S6 | LY9; CD229; SLAMF3; hly9; mLY9 | CD229 (Ly-9) | REA963 | PE | 130-116-003 | Miltenyi Biotec | 4063 |
| S6 | PDCD1LG2; B7DC; Btdc; CD273; PD-L2; PDCD1L2; PDL2; bA574F11.2 | CD273 (PD-L2) | REA985 | FITC | 130-116-562 | Miltenyi Biotec | 80380 |
| S6 | ACKR1; CCBP1; CD234; DARC; DARC/ACKR1; Dfy; FY; GPD; GpFy; WBCQ1 | CD234 (DARC) | REA376 | PE | 130-125-845 | Miltenyi Biotec | 2532 |
| S6 | ICOS; AILIM; CD278; CVID1 | CD278 (ICOS) | REA192 | FITC | 130-100-732 | Miltenyi Biotec | 29851 |
| S6 | IL2RA; CD25; IDDM10; IL2R; IMD41; TCGFR; p55 | CD25 | REA945 | PE | 130-115-534 | Miltenyi Biotec | 3559 |
| S6 | TLR3; CD283; IIAE2 | CD283 (TLR3) | TLR 3.7 | FITC | 130-100-000 | Miltenyi Biotec | 7098 |
| S6 | LEPR; CD295; LEP-RD; OB-R; OBR; LEPR | CD295 (LEPR) | REA361 | PE | 130-125-203 | Miltenyi Biotec | 3953 |
| S6 | NRP1; BDCA4; CD304; NP1; NRP; VEGF165R | CD304 (BDCA-4) | REA774 | FITC | 130-111-892 | Miltenyi Biotec | 8829 |
| S6 | CLEC10A; CD301; CLECSF13; CLECSF14; HML; HML2; MGL | CD301 (CLEC10A) | REA586 | PE | 130-109-582 | Miltenyi Biotec | 10462 |
| S6 | ADGRE2; CD312; EMR2; VBU | CD312 (EMR2) | REA302 | FITC | 130-104-582 | Miltenyi Biotec | 30817 |
| S6 | CD302; BIMLEC; CLEC13A; DCL-1; DCL1 | CD302 (CLEC13A) | REA509 | PE | 130-107-796 | Miltenyi Biotec | 9936 |
| S6 | CDCP1; CD318; SIMA135; TRASK | CD318 (CDCP1) | REA194 | FITC | 130-101-212 | Miltenyi Biotec | 64866 |
| S6 | LAIR1; CD305; LAIR-1 | CD305 (LAIR-1) | REA447 | PE | 130-126-091 | Miltenyi Biotec | 3903 |
| S6 | SIGLEC7; AIRM1; CD328; CDw328; D-siglec; QA79; SIGLEC-7; SIGLEC19P; SIGLECP2; p75; p75/AIRM1 | CD328 (Siglec-7) | REA214 | FITC | 130-100-998 | Miltenyi Biotec | 27036 |
| S6 | FCRL1; CD307a; FCRH1; IFGP1; IRTA5 | CD307a (FcRL1) | REA440 | PE | 130-106-448 | Miltenyi Biotec | 115350 |
| S6 | SLAMF6; CD352; KALI; KALIb; Ly108; NTB-A; NTBA; SF2000 | CD352 (NTB-A) | REA339 | FITC | 130-125-981 | Miltenyi Biotec | 114836 |
| S6 | FCRL2; CD307b; FCRH2; IFGP4; IRTA4; SPAP1; SPAP1A; SPAP1B; SPAP1C | CD307b (FcRL2) | REA474 | PE | 130-123-295 | Miltenyi Biotec | 79368 |
| S6 | CD37; GP52-40; TSPAN26 | CD37 | REA366 | FITC | 130-105-243 | Miltenyi Biotec | 951 |
| S6 | KDR; CD309; FLK1; VEGFR; VEGFR2 | CD309 (VEGFR-2) | REA1046 | PE | 130-117-910 | Miltenyi Biotec | 3791 |
| S6 | ITGA2B; ITGB3; BDPLT16; BDPLT2; CD41; CD41B; GP2B; GPIIb; GT; GTA; HPA3; PPP1R93; CD61; GP3A; GPIIIa | CD41/CD61 | REA607 | FITC | 130-124-887 | Miltenyi Biotec | 3674; 3690 |
| S6 | SLAMF7; 19A; CD319; CRACC; CS1 | CD319 (CRACC) | REA150 | PE | 130-119-779 | Miltenyi Biotec | 57823 |
| S6 | CD46; AHUS2; MCP; MIC10; TLX; TRA2.10 | CD46 | REA312 | FITC | 130-104-507 | Miltenyi Biotec | 4179 |
| S6 | FCGR2A; FCGR2B; FCGR2C; CD32; CD32A; CDw32; FCG2; FCGR21; FcGR; IGFR2; FCGR2A; CD32B; FCGR2; CD32C; CDW32; FCRIIC | CD32 | REA997 | PE | 130-116-599 | Miltenyi Biotec | 2212; 2213; 9103 |
| S6 | ITGA3; CD49C; FRP-2; GAP-B3; GAPB3; ILNEB; MSK18; VCA-2; VL3A; VLA3a | CD49c | REA360 | FITC | 130-105-364 | Miltenyi Biotec | 3675 |
| S6 | SIGLEC9; CD329; CDw329; FOAP-9; OBBP-LIKE; siglec-9 | CD329 (Siglec-9) | REA492 | PE | 130-107-604 | Miltenyi Biotec | 27180 |
| S6 | ITGAV; CD51; MSK8; VNRA; VTNR | CD51 | REA181 | FITC | 130-100-704 | Miltenyi Biotec | 3685 |
| S6 | SLAMF8; BLAME; CD353; SBBI42 | CD353 (SLAMF8) | REA394 | PE | 130-106-675 | Miltenyi Biotec | 56833 |
| S6 | CEACAM8; CD66b; CD67; CGM6; NCA-95 | CD66b | REA306 | FITC | 130-123-694 | Miltenyi Biotec | 1088 |
| S6 | TNFRSF18; AITR; CD357; GITR; GITR-D | CD357 (GITR) | REA1007 | PE | 130-116-840 | Miltenyi Biotec | 8784 |
| S6 | CD81; CVID6; S5.7; TAPA1; TSPAN28 | CD81 | REA513 | FITC | 130-123-697 | Miltenyi Biotec | 975 |
| S6 | CLEC12A; CD371; CLL-1; CLL1; DCAL-2; MICL | CD371 (CLEC12A) | REA431 | PE | 130-122-920 | Miltenyi Biotec | 160364 |
| S6 | LILRB1; CD85J; ILT-2; ILT2; LIR-1; LIR1; MIR-7; MIR7; PIR-B; PIRB | CD85j (ILT2) | REA998 | FITC | 130-116-614 | Miltenyi Biotec | 10859 |
| S6 | ICAM3; CD50; CDW50; ICAM-R | CD50 (ICAM-3) | REAL396 | PE | 130-119-597 | Miltenyi Biotec | 3385 |
| S6 | KLRD1; CD94 | CD94 | REA113 | FITC | 130-123-678 | Miltenyi Biotec | 3824 |
| S6 | ITGAV; ITGB3; CD51; MSK8; VNRA; VTNR; BDPLT16; BDPLT2; CD61; GP3A; GPIIIa; GT | CD51/CD61 | REA1099 | PE | 130-119-052 | Miltenyi Biotec | 3685; 3690 |
| S6 | CD74; DHLAG; HLADG; II; Ia-GAMMA | CLIP | REA296 | FITC | 130-104-401 | Miltenyi Biotec | 972 |
| S6 | CD58; LFA-3; LFA3; ag3 | CD58 (LFA-3) | REA1098 | PE | 130-118-612 | Miltenyi Biotec | 965 |
| S6 | FCER1A; FCE1A; FcERI | FcεRIα | REA758 | FITC | 130-110-726 | Miltenyi Biotec | 2205 |
| S6 | CD59; 16.3A5; 1F5; EJ16; EJ30; EL32; G344; HRF-20; HRF20; MAC-IP; MACIF; MEM43; MIC11; MIN1; MIN2; MIN3; MIRL; MSK21; p18-20 | CD59 | REA496 | PE | 130-120-048 | Miltenyi Biotec | 966 |
| S6 |  | Fibroblast | REA165 | FITC | 130-100-134 | Miltenyi Biotec |  |
| S6 | SELL; CD62L; LAM1; LECAM1; LEU8; LNHR; LSEL; LYAM1; PLNHR; TQ1 | CD62L | REA615 | PE | 130-113-625 | Miltenyi Biotec | 6402 |
| S6 | LGALS3; CBP35; GAL3; GALBP; GALIG; L31; LGALS2; MAC2 | Galectin-3 | REA1072 | FITC | 130-118-647 | Miltenyi Biotec | 3958 |
| S6 | SELP; CD62; CD62P; GMP140; GRMP; LECAM3; PADGEM; PSEL | CD62P | REA389 | PE | 130-117-710 | Miltenyi Biotec | 6403 |
| S6 | H2AFX; H2A.X; H2A/X; H2AX | H2AX pS139 | REA502 | FITC | 130-118-339 | Miltenyi Biotec | 3014 |
| S6 | CD69; AIM; BL-AC/P26; CLEC2C; EA1; GP32/28; MLR-3 | CD69 | FN50 | PE | 130-113-524 | Miltenyi Biotec | 969 |
| S6 | IKZF2; ANF1A2; HELIOS; ZNF1A2; ZNFN1A2 | Helios | REA829 | FITC | 130-112-629 | Miltenyi Biotec | 22807 |
| S6 | CD79B; AGM6; B29; IGB | CD79b | REA120 | PE | 130-123-737 | Miltenyi Biotec | 974 |
| S6 | HIST1H3A; HIST1H3B; HIST1H3D; HIST1H3E; HIST1H3C; HIST1H3F; HIST1H3I; HIST1H3G; HIST1H3H; H3/A; H3FA; H3/l; H3FL; H3/b; H3FB; H3.1; H3/d; H3FD; H3/c; H3FC; H3/i; H3FI; H3.f; H3/f; H3FF; H3/j; H3FJ; H3/h; H3FH; H3/k; H3F1K; H3FK | Histone H3 pS28 | REA379 | FITC | 130-125-210 | Miltenyi Biotec | 8350; 8351; 8352; 8353; 8354; 8355; 8356; 8357; 8358; 8968 |
| S6 | C5AR1; C5A; C5AR; C5R1; CD88 | CD88 (C5AR) | REA1213 | PE | 130-123-379 | Miltenyi Biotec | 728 |
| S6 | HLA-A; HLAA | HLA-A2 | REA517 | FITC | 130-118-969 | Miltenyi Biotec | 3105 |
| S6 | CD8B; CD8B1; LEU2; LY3; LYT3; P37 | CD8b | REAL398 | PE | 130-119-598 | Miltenyi Biotec | 926 |
| S6 | HLA-A; HLAA | HLA-A2, A28 | REA142 | FITC | 130-099-601 | Miltenyi Biotec | 3105 |
| S6 | CLEC7A; BGR; CANDF4; CD369; CLECSF12; DECTIN1; SCARE2 | Dectin-1 | REA515 | PE | 130-121-993 | Miltenyi Biotec | 64581 |
| S6 | HLA-DMA; HLA-DMB; D6S222E; DMA; HLADM; RING6; D6S221E; RING7 | HLA-DM | REA406 | FITC | 130-124-224 | Miltenyi Biotec | 3108; 3109 |
| S6 | ADGRG1; BFPP; BPPR; GPR56; TM7LN4; TM7XN1 | GPR56 | REA467 | PE | 130-125-212 | Miltenyi Biotec | 9289 |
| S6 | IGKC; HCAK1D; Km; IGKC | Ig κ Light Chain | IS11-24D5 | FITC | 130-123-669 | Miltenyi Biotec | 3514 |
| S6 | IGHA2 | IgA2 | REA995 | PE | 130-117-763 | Miltenyi Biotec | 3494 |
| S6 | IGLC1; IGLC2; IGLC3; IGLC6; IGLC7; IGLC; C7 | Ig λ Light Chain | IS7-24C7 | FITC | 130-093-040 | Miltenyi Biotec | 3537; 3538; 3539; 3542; 28834 |
| S6 | ITGA9; ITGB1; ALPHA-RLC; ITGA4L; RLC; CD29; FNRB; GPIIA; MDF2; MSK12; VLA-BETA; VLAB | Integrin α9β1 | REA483 | PE | 130-107-128 | Miltenyi Biotec | 3680; 3688 |
| S6 | IGHG1; IGHG2; IGHG3; IGHG4; IgG3 | IgG | IS11-3B2.2.3 | FITC | 130-118-340 | Miltenyi Biotec | 3500; 3501; 3502; 3503 |
| S6 | KLRG1; 2F1; CLEC15A; MAFA; MAFA-2F1; MAFA-L; MAFA-LIKE | KLRG1 | REA261 | PE | 130-120-426 | Miltenyi Biotec | 10219 |
| S6 | ABCC1; ABC29; ABCC; GS-X; MRP; MRP1 | MRP1 | REA481 | FITC | 130-107-466 | Miltenyi Biotec | 4363 |
| S6 | POU2F2; OCT2; OTF2; Oct-2 | Oct-2 | REA251 | PE | 130-103-633 | Miltenyi Biotec | 5452 |
| S6 | ALP; ALPI; ALPP; IAP; PALP; PLAP; PLAP-1 | PLAP | REA1089 | FITC | 130-118-301 | Miltenyi Biotec | 250 |
| S6 | CTNND1; BCDS2; CAS; CTNND; P120CAS; P120CTN; p120; p120(CAS); p120(CTN) | p120 Catenin pT310 | REA153 | PE | 130-104-296 | Miltenyi Biotec | 1500 |
| S6 | TRBV2; TRB; TCRBV22S1A2N1T; TCRBV2S1; TCRB@; TRB | TCR Vβ2 | REA654 | FITC | 130-110-060 | Miltenyi Biotec | 28620; 6957 |
| S6 | RPS6; S6 | S6 pS235/pS236 | REA454 | PE | 130-124-239 | Miltenyi Biotec | 6194 |
| S6 | TRA; TRB; IMD7; TCRA; TCRD@; TRAC; TRA; TCRB@; TRB | TCRα/β | REA652 | FITC | 130-113-538 | Miltenyi Biotec | 6955; 6957 |
| S6 | SIGLEC5; SIGLEC14; CD170; CD33L2; OB-BP2; OBBP2; SIGLEC-5 | Siglec-5/Siglec-14 | REA393 | PE | 130-106-018 | Miltenyi Biotec | 8778; 100049587 |
| S6 | TNF; DIF-alpha; TNFA; TNFSF2; TNLG1F; TNF | TNF-α | cA2 | FITC | 130-120-490 | Miltenyi Biotec | 7124 |
| S6 | SELPLG; CD162; CLA; PSGL-1; PSGL1 | Slan (M-DC8) | REA1050 | PE | 130-117-918 | Miltenyi Biotec | 6404 |
| S6 | BMI1; FLVI2/BMI1; PCGF4; RNF51; flvi-2/bmi-1 | BMI-1 | REA438 | FITC | 130-124-226 | Miltenyi Biotec | 648 |
| S6 | TRBV5-3; TRB; TCRBV5S3; TCRBV5S5P; TRBV53; TCRB@; TRB | TCR Vβ5.3 | REA670 | PE | 130-110-139 | Miltenyi Biotec | 28612; 6957 |
| S6 | ITGAM; CD11B; CR3A; MAC-1; MAC1A; MO1A; SLEB6 | CD11b | REA713 | FITC | 130-110-552 | Miltenyi Biotec | 3684 |
| S6 | TGFBR2; AAT3; FAA3; LDS1B; LDS2; LDS2B; MFS2; RIIC; TAAD2; TBR-ii; TBRII; TGFR-2; TGFbeta-RII | TGF-β RII | REA903 | PE | 130-115-024 | Miltenyi Biotec | 7048 |
| S6 | PTPRJ; CD148; DEP1; HPTPeta; R-PTP-ETA; SCC1 | CD148 | REA204 | FITC | 130-101-221 | Miltenyi Biotec | 5795 |
| S6 | BDNF; ANON2; BULN2 | BDNF | REA358 | PE | 130-105-120 | Miltenyi Biotec | 627 |
| S6 | SIRPA; BIT; CD172A; MFR; MYD-1; P84; PTPNS1; SHPS1; SIRP | CD172a (SIRPα) | REA144 | FITC | 130-099-896 | Miltenyi Biotec | 140885 |
| S6 | CA9; CAIX; MN | Carbonic Anhydrase 9 | REA658 | PE | 130-123-299 | Miltenyi Biotec | 768 |
| S6 | TNFSF14; CD258; HVEML; LIGHT; LTg | CD258 (LIGHT) | REA244 | FITC | 130-103-658 | Miltenyi Biotec | 8740 |
| S6 | NECTIN1; CD111; CLPED1; ED4; HIgR; HV1S; HVEC; OFC7; PRR; PRR1; PVRL1; PVRR; PVRR1; SK-12; nectin-1 | CD111 | R1.302 | PE | 130-103-833 | Miltenyi Biotec | 5818 |
| S6 | IFNA1; IFNA2; IFNA17; IFNA8; IFNA13; IFNA21; IFNA4; IFNA10; IFNA14; IFNA5; IFNA16; IFNA6; IFNA7; IFL; IFN; IFN-ALPHA; IFN-alphaD3; IFNA@; IFNA1; IFN-alphaA; IFNAB; INFA2; IFNA2; IFN-alphaI; IFNA; INFA; LEIF2C1; IFN-alphaB; LeIF F; leIF-F; IFN-alpha4a; INFA4; IFN-alphaC; IFN-alphaH; LEIF2H; IFN-alpha-5; IFN-alphaG; INA5; INFA5; leIF G; IFN-alpha-16; IFN-alphaO; IFN-alphaK; IFN-alphaJ; IFNA-J | IFN-α | REA1013 | FITC | 130-116-872 | Miltenyi Biotec | 3439; 3440; 3451; 3445; 3447; 3452; 3441; 3446; 3448; 3442; 3449; 3443; 3444 |
| S6 | CSF3R; CD114; GCSFR; SCN7 | CD114 | REA1082 | PE | 130-119-006 | Miltenyi Biotec | 1441 |
| S6 | IFNG; IFG; IFI | IFN-γ | REA600 | FITC | 130-113-497 | Miltenyi Biotec | 3458 |
| S6 | TNFRSF1B; CD120b; TBPII; TNF-R-II; TNF-R75; TNFBR; TNFR1B; TNFR2; TNFR80; p75; p75TNFR | CD120b (TNF-RII) | REA520 | PE | 130-123-778 | Miltenyi Biotec | 7133 |
| S6 | CD38; ADPRC 1; ADPRC1 | CD38 | REA671 | PE | 130-117-717 | Miltenyi Biotec | 952 |
| S6 | PTPRC; B220; CD45; CD45R; GP180; L-CA; LCA; LY5; T200 | CD45RA | REA562 | FITC | 130-113-365 | Miltenyi Biotec | 5788 |
| S6 | CD3E; IMD18; T3E; TCRE | CD3 | REA613 | PE | 130-113-139 | Miltenyi Biotec | 916 |

**Table S2: List of anti-mouse antibodies.**

| **Figure** | **Gene Name** | **CD nomenclature** | **Clone** | **Conjugate** | **Order Number** | **Company** | **Gene Identifier (NCBI)** |
| --- | --- | --- | --- | --- | --- | --- | --- |
| S1a | CD4; L3T4; LY-4 | CD4 | GK1.5 | FITC | 130-120-819 | Miltenyi Biotec | 12504 |
| S1a | B220; CD45; CD45R; L-CA; LOC; LY-5; LYT-4; PTPRC; T200 | CD45R | RA3-6B2 | FITC | 130-118-462 | Miltenyi Biotec | 19264 |
| 3a | Cd2 | CD2 | REA959 | FITC | 130-115-958 | Miltenyi Biotec | 12481 |
| 3a | Cd4 | CD4 | REA604 | PE | 130-116-509 | Miltenyi Biotec | 12504 |
| 3a | Cd5 | CD5 | REA421 | PE | 130-120-305 | Miltenyi Biotec | 12507 |
| 3a | Cd8a | CD8a | REA601 | PE | 130-123-781 | Miltenyi Biotec | 12525 |
| 3a | Cd8b1 | CD8b | REA793 | FITC | 130-111-710 | Miltenyi Biotec | 12526 |
| 3a | Itgal | CD11a/CD18 | REA880 | FITC | 130-114-422 | Miltenyi Biotec | 16408 |
| 3a | Itgam | CD11b | REA592 | PE | 130-113-806 | Miltenyi Biotec | 16409 |
| 3a | Itgax | CD11c | REA754 | FITC | 130-110-837 | Miltenyi Biotec | 16411 |
| 3a | Cd14 | CD14 | REA934 | FITC | 130-115-557 | Miltenyi Biotec | 12475 |
| 3a | Fut4 | CD15 | REA321 | PE | 130-117-801 | Miltenyi Biotec | 14345 |
| 3a | Fcer2a | CD23 | B3B4 | PE | 130-102-611 | Miltenyi Biotec | 14127 |
| 3a | Il2ra | CD25 | REA568 | PE | 130-120-766 | Miltenyi Biotec | 16184 |
| 3a | Cd27 | CD27 | REA499 | FITC | 130-114-165 | Miltenyi Biotec | 21940 |
| 3a | Itgb1 | CD29 | HMß1-1 | FITC | 130-102-503 | Miltenyi Biotec | 16412 |
| 3a | Cd38 | CD38 | REA616 | FITC | 130-122-955 | Miltenyi Biotec | 12494 |
| 3a | Ptprc | CD45 | REA737 | FITC | 130-110-796 | Miltenyi Biotec | 19264 |
| 3a | Ptprc | CD45R (B220) | RA3-6B2 | FITC | 130-118-462 | Miltenyi Biotec | 19264 |
| 3a | Thy1 | CD90.2 | 30-H12 | PE | 130-102-960 | Miltenyi Biotec | 21838 |
| 3a | Fas | CD95 (FAS) | REA453 | PE | 130-119-657 | Miltenyi Biotec | 14103 |
| 3a | Itgb4 | CD104 | REA456 | PE | 130-106-923 | Miltenyi Biotec | 192897 |
| 3a | Vcam1 | CD106 | REA971 | PE | 130-116-323 | Miltenyi Biotec | 22329 |
| 3a | Il7r | CD127 | REA680 | PE | 130-119-795 | Miltenyi Biotec | 16197 |
| 3a | Cd151 | CD151 | REA561 | PE | 130-109-005 | Miltenyi Biotec | 12476 |
| 3a | Cd180 | CD180 (RP105) | REA957 | PE | 130-115-929 | Miltenyi Biotec | 17079 |
| 3a | Cxcr3 | CD183 (CXCR3) | REA724 | PE | 130-111-087 | Miltenyi Biotec | 12766 |
| 3a | Cxcr4 | CD184 (CXCR4) | REA107 | PE | 130-118-682 | Miltenyi Biotec | 12767 |
| 3a | Cxcr5 | CD185 (CXCR5) | REA215 | PE | 130-123-753 | Miltenyi Biotec | 12145 |
| 3a | Ccr6 | CD196 (CCR6) | REA277 | PE | 130-126-497 | Miltenyi Biotec | 12458 |
| 3a | Cd200r1,2,3,4 | CD200R | REA850 | FITC | 130-112-529 | Miltenyi Biotec | 57781 |
| 3a | Adgre1 | F4/80 | REA126 | PE | 130-116-499 | Miltenyi Biotec | 13733 |
| 3a | Ly6g | Gr-1 | REA810 | FITC | 130-112-311 | Miltenyi Biotec | 546644 |
| 3a | H2kb | H2kb | REA392 | FITC | 130-106-415 | Miltenyi Biotec | 14972 |
| 3a | Ighd | IgD | REA772 | FITC | 130-111-495 | Miltenyi Biotec | 389797 |
| 3a | Ighm | IgM | X-54 | PE | 130-095-908 | Miltenyi Biotec | 16019 |
| 3a | Mki67 | Ki67 | REA183 | FITC | 130-117-803 | Miltenyi Biotec | 17345 |
| 3a | Klrg1 | KLRG1 | 2F1 | PE | 130-102-408 | Miltenyi Biotec | 50928 |
| 3a | Ly6c | Ly-6C | REA796 | FITC | 130-111-915 | Miltenyi Biotec | 56778 |
| 3a | Ly6g | Ly-6G | REA526 | FITC | 130-120-820 | Miltenyi Biotec | 546644 |
| 3a | H2-Ab1 | MHC Class II | M5/114.15.2 | PE | 130-102-896 | Miltenyi Biotec | 14961 |
| 3a | Klrb1c | NK1.1 | PK136 | PE | 130-116-504 | Miltenyi Biotec | 17059 |
| 3a | Klrc1 | NKG2A/C/E | 20d5 | FITC | 130-105-619 | Miltenyi Biotec | 16641 |
| 3a | Pcna | PCNA | REA858 | FITC | 130-114-702 | Miltenyi Biotec | 18538 |
| 3a | Rorc | RORg | REA278 | PE | 130-123-248 | Miltenyi Biotec | 19885 |
| 3a | Ly6a | Sca-1 | REA422 | PE | 130-116-489 | Miltenyi Biotec | 110454 |
| 3a | Tcrb | TCRβ | REA318 | FITC | 130-126-505 | Miltenyi Biotec | 21577 |
| 3a | Tcrg/Tcrd | TCRγ/δ | REA633 | FITC | 130-128-067 | Miltenyi Biotec | 110067/110066 |
| 3a | Klra1 | Anti-Ly-49A/D | REA853 | PE | 130-112-938 | Miltenyi Biotec |  |
